# Scalable irradiance-adaptive electrochromic shading for photothermal regulation

**DOI:** 10.1038/s41467-026-76115-0

**Published:** 2026-07-28

**Authors:** Wenting Wu, Xueyang Wang, Alvin Wei Ming Tan, Yangyang Xin, Yixuan Jiang, Tan Hu, Hui Wang, Yawei Jiang, Pooi See Lee

**Affiliations:** 1 School of Materials Science and Engineering, Nanyang Technological University, Singapore, Singapore; 2 Singapore-HUJ Alliance for Research and Enterprise (SHARE), Smart Grippers for Soft Robotics (SGSR), Campus for Research Excellence and Technological Enterprise (CREATE), Singapore, Singapore

**Keywords:** Electronic devices, Electrochemistry, Heat, Light stress

## Abstract

Agricultural production in hot climates requires effective photothermal regulation under fluctuating solar conditions. Electrochromic devices offer a promising route for dynamic photothermal regulation, yet their deployment in real-world agricultural environments remains limited by challenges in scalability, durability, and system-level integration. Here, we show a scalable, solar-powered, irradiance-adaptive electrochromic shading system that autonomously regulates photosynthetically active radiation and suppresses ultraviolet and near-infrared radiation under fluctuating outdoor conditions without external energy input. The electrochromic devices exhibit large optical contrast and robust operational stability during tropical outdoor exposure. Field deployment in tropical agriculture reduces crop-surrounding temperature by up to 3.8 °C while maintaining optimal conditions for photosynthesis. Consequently, crops grown under electrochromic shading exhibit 23.7–64% increases in vitamin C, total sugar, and pigments, together with a 235% increase in biomass yield compared with a conventional shading system. Cultivation modeling further reveals substantial cooling-energy savings across diverse climate zones during hot seasons.

## Abstract (web summary)

A solar-powered electrochromic shading system dynamically regulates sunlight intensity for crops without external energy input. In tropical field tests, it reduces temperatures, improves crop quality, and increases biomass yield by up to 235% compared with conventional shading.

## Introduction

As climate change intensifies, global temperatures continue to rise while arable land availability declines, placing increasing pressure on food production systems worldwide^1–4^. In response, controlled-environment agriculture, particularly greenhouse-type cultivation, has emerged as a promising strategy for localized and resource-efficient food production^5,6^. However, maintaining crop productivity in outdoor or semi-covered cultivation systems remains challenging, particularly in hot climates, where excessive heat and fluctuating solar irradiance can strongly perturb crop microclimates and suppress photosynthetic performance.

In subtropical and tropical climates, active cooling technologies such as fans, pumps, and air conditioning systems are widely employed to regulate temperature in greenhouse cultivation systems^7,8^. Although effective, these approaches consume substantial electricity and water and offer little control over crop light environments. By contrast, passive cooling strategies, such as shading and reflective coverings, reduce temperature without additional water or energy consumption^7,8^. However, most passive materials rely on broadband blocking or reflection of incoming solar radiation, indiscriminately attenuating ultraviolet (UV, 280–400 nm), visible light (VIS, 400–780 nm, including photosynthetically active radiation (PAR, 400–700 nm)), and near-infrared (NIR, 780–2500 nm) wavelengths^9^. As a result, thermal regulation is often achieved at the expense of reduced PAR availability, making it difficult to simultaneously meet cooling demands and photosynthetic requirements.

To overcome these limitations, emerging agricultural photothermal control strategies have been developed in heat-intensive regions. Photoselective, photoluminescent, and photoconversion covers enable moderate shading while tailoring spectral composition to crop-specific needs^10–13^. Radiative cooling covers enhance heat dissipation through additional infrared emission^14–16^. Together, these approaches demonstrate that coordinated regulation of light and heat is a viable route for improving crop microclimates^17,18^. Nevertheless, their optical properties are typically fixed once designed and fabricated, limiting adaptability under fluctuating outdoor irradiance. Current photothermal control materials generally either block broad portions of the solar spectrum^19^ or transmit PAR within a predefined spectral window^9,20–22^, making it difficult to dynamically maintain crop-optimal photosynthetic and thermal conditions under fluctuating solar irradiance. Thus, the current state of the art in agricultural photothermal control is severely constrained by materials with static optical properties.

These limitations highlight the need for adaptive strategies capable of dynamically regulating both the intensity and spectral quality of PAR while simultaneously suppressing excess heat gain. PAR accounts for nearly 50% of total solar energy and plays a dual role in governing photosynthesis and contributing to thermal load^23,24^. Precise control of PAR is therefore critical for crop performance: excessive irradiance can induce photoinhibition and saturation, whereas insufficient irradiance limits carbon assimilation and growth^25^. Electrochromic (EC) technologies are distinct in this context because they offer reversible and on-demand tuning of solar-spectrum transmission through switching between visible-colored and bleached states, enabling adaptive photothermal regulation^26,27^. In principle, EC shading can simultaneously regulate PAR intensity, modify spectral quality, and suppress heat-inducing radiation in response to changing environmental conditions. This combination of dynamic spectral tunability and real-time environmental responsiveness differentiates EC shading from existing agricultural photothermal-control materials with static optical properties. However, despite this potential, translating EC technologies from lab demonstrations to outdoor agricultural environments remains challenging because it requires simultaneous achievement of large-area scalability, device durability, and autonomous system-level operation.

**Fig. 1:**
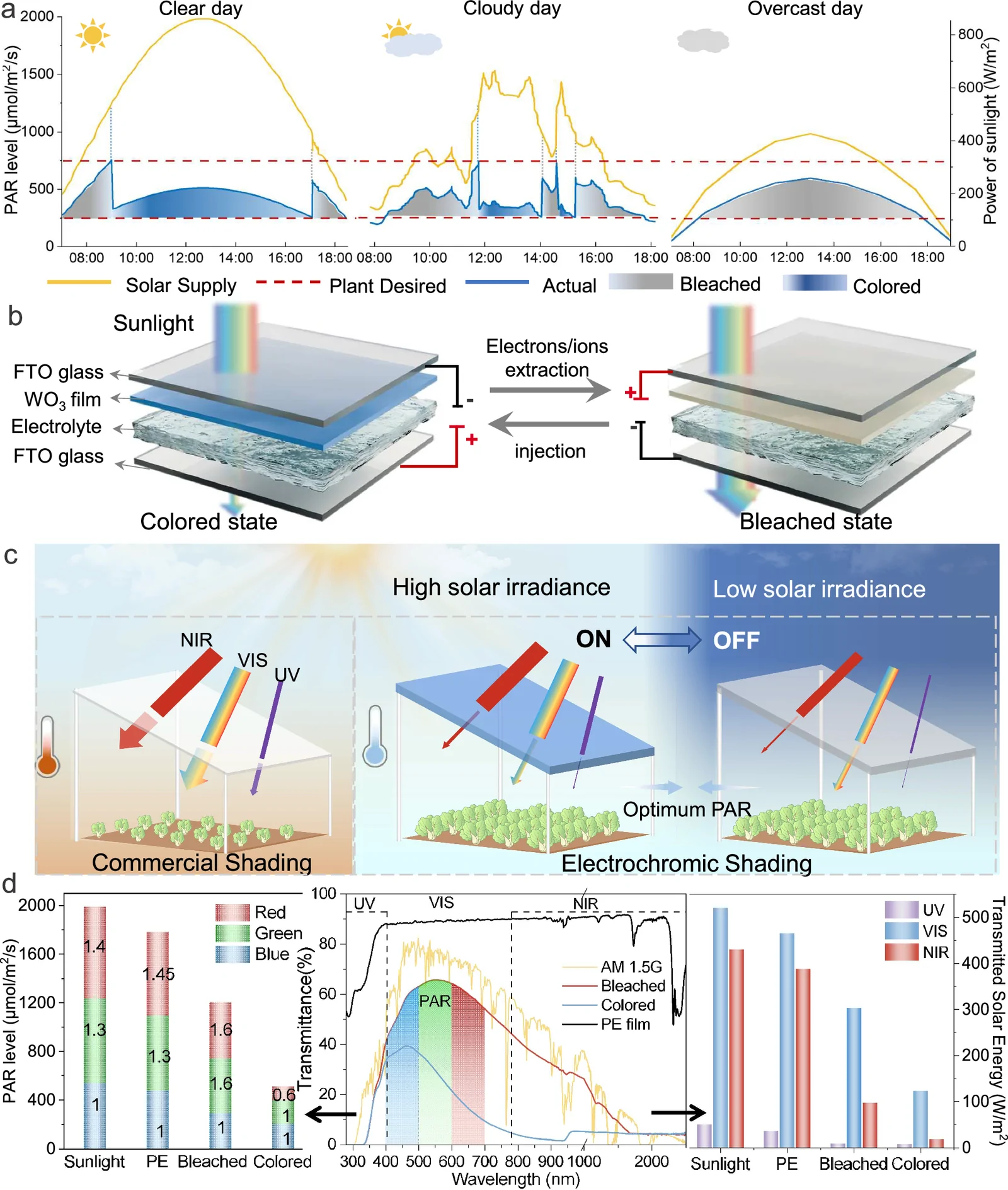
Design and characterization of EC shading. **a** The design concept of irradiance-adaptive and light-adjustable EC shading for cultivation system under different sky conditions (clear day, cloudy day, and overcast day, the PAR data are derived from refs. ^68,69^) to keep the PAR level in the plant’s desired range. **b** The device structure of EC shading under colored/bleached states upon electrons/ions extraction/injection. **c** Schematic illustration of irradiance-adaptive and light-adjustable EC shading working modes with colored/bleached states under high/low solar irradiance. The commercial shading using PE film was set as a reference. **d** Left calculated PAR photon flux levels under unshaded sunlight, commercial PE film, and the EC shading in colored/bleached states, with the corresponding red-, green-, and blue-band photo-flux contributions indicated in each bar. Center transmittance spectra (0.28–2.5 µm) of the EC shading in colored/bleached states, together with commercial PE film, overlaid with the AM 1.5 G solar spectrum; the PAR region is highlighted. Right calculated transmitted solar energy in the UV/VIS/NIR regions under unshaded sunlight, PE film, and EC shading in colored/bleached states. NIR, VIS and UV denote near-infrared, visible and ultraviolet light, respectively. AM1.5 G, air mass 1.5 global; PAR, photosynthetically active radiation; PE, polyethylene.

Here, we develop a scalable irradiance-adaptive EC shading platform for photothermal regulation in hot climates. The platform is enabled by robust large-area EC devices with large optical contrast, fast switching, optical memory exceeding 21600 s, and maintains stable electrochromic performance over a 2-month outdoor exposure test under tropical conditions. Building on this device-level robustness, we integrate the EC devices into a modular, solar-powered shading system that autonomously switches between bleached and colored states in response to real-time solar irradiance. This enables irradiance-adaptive regulation of visible (PAR) transmission under fluctuating outdoor conditions, maintaining PAR within optimal photosynthetic ranges (Fig. 1a), while suppressing excess radiative heat through passive UV suppression and substrate-dominated NIR attenuation. Additionally, state-dependent modulation of PAR quality, particularly the blue-to-red (B/R) light ratio, further supports photosynthesis under varying light conditions.

Field deployment in tropical outdoor environments confirms that EC shading lowers crop-surrounding temperature while maintaining PAR and daily light integral (DLI) within optimal ranges for leafy vegetable growth. These favorable photothermal conditions improve nutritional quality and substantially increase crop yield compared with commercial polyethylene (PE) shading and unshaded conditions. Furthermore, cultivation modeling across diverse global climate zones during hot seasons confirms the scalability and effectiveness of the EC shading system, revealing robust cooling-energy savings under heat-intensive conditions. Together, this work establishes electrochromic shading as a practical, scalable platform for adaptive photothermal regulation in real-world agricultural systems.

## Results

### Autonomous electrochromic shading for farm cultivation system

The dynamic EC shading for an outdoor farm cultivation system is constructed based on large-area 30 × 30 cm^2^ electrochromic devices (ECDs). As shown in Fig. 1b, the ECD consists of fluorine-doped tin oxide (FTO) glass as the transparent conductive substrate for electron transport; a WO_3_ film as the EC layer, and a LiClO_4_-Propylene Carbonate (PC)-based electrolyte to accommodate ions. Reversible insertion and extraction of electrons from the FTO substrate and ions from the electrolyte into and out of the WO_3_ layer enable the ECD to switch between colored and bleached states under applied bias.

For outdoor operation, the ECD state is autonomously controlled by a light-sensor-based module powered by an integrated solar panel, enabling irradiance-adaptive switching under changing outdoor conditions. This state transition provides coordinated photothermal regulation by reducing radiative heat gain through passive UV suppression and NIR attenuation, while tuning both the intensity and spectral composition of PAR (Fig. 1c, d). As shown in Fig.1d (center and right), the ECD in both colored and bleached states strongly suppresses UV transmission and maintains low NIR transmittance, while providing switchable visible light transmission. Mechanistically, UV suppression is dominated by the passive filtering of the FTO-glass substrates (Supplementary Fig. 1), whereas NIR attenuation is also substantial in both states and primarily governed by the FTO-glass baseline, with additional moderate electrochromic modulation. These effects are quantified for the complete device using EN 410-type band-integrated metrics (Supplementary Table 1): the ECD blocks 83.7%/81.8% (colored/bleached) of UV radiation (280–400 nm), and attenuates 96.3%/77.6% (colored/bleached) of NIR radiation (780–2500 nm), respectively. This spectral attenuation is further reflected in the reduced transmitted solar energy across these regions, leading to a lower radiative heat load (Supplementary Table 2).

In addition, the switchable visible states enable dynamic regulation of PAR intensity toward the crop-optimal range (Fig. 1a) and of spectral quality. As summarized in Fig. 1d (left) and Supplementary Table 3, the EC shading modulates both the total PAR photon flux and the relative red (600–700 nm), green (500–600 nm) and blue (400–500 nm) contributions. Under high solar irradiance, the EC shading switches to the colored state, increasing the relative blue light fraction (B/R = 1:0.6) compared with unshaded sunlight (B/R = 1:1.4). Such a blue-enriched spectrum is known to modulate stomatal behavior and photoprotective signaling^28,29^, and is therefore expected to support photosynthetic performance under fluctuating irradiance. Conversely, under low solar irradiance, the EC shading reverts to the bleached state with a higher relative red-light fraction (B/R = 1:1.6), which can favor efficient light harvesting and support carbon assimilation under light-limited conditions^30^, thereby benefiting plant growth. This state-dependent shift in B/R ratio is consistent with established photobiological responses of plants, where blue- and red-light fractions differentially regulate stomatal behavior and photosynthetic acclimation.

In contrast, commercial PE film transmits most NIR and VIS radiation with minimal spectral selectivity or dynamic modulation. As shown in Fig. 1d (center and right), the PE film exhibits ~90% transmittance over 400–2250 nm and allows 888.2 W/m^2^ of solar energy to enter the growing chamber under standard AM 1.5 G solar irradiation (1000 W/m^2^), providing minimal regulation of thermal load. Consistently, Fig. 1d (left) shows that the PE shading maintains a fixed B/R ratio (1:1.45), indicating limited adaptability of the light environment compared with the EC shading.

### Robust large-area ECDs for outdoor operation

**Fig. 2:**
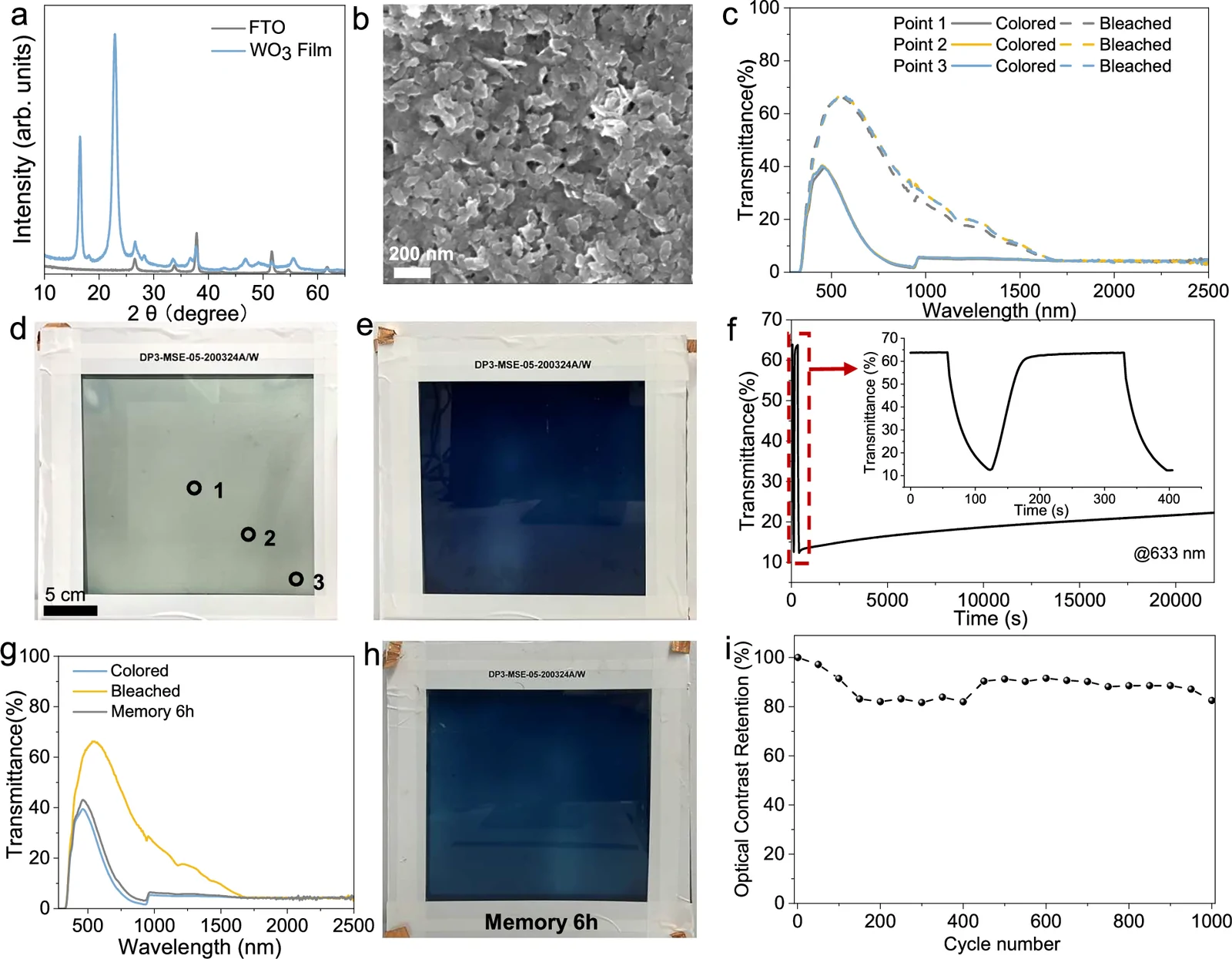
Characterization and optical performances of 30 × 30 cm^2^ ECDs. **a** The XRD patterns of inkjet-printed WO_3_ film on FTO substrate and bare FTO substrate. **b** The SEM image of the WO_3_ film. **c** Transmittance spectra of the ECD in colored/bleached states from center to side points under applied potentials of −3.5 V and 2 V. Optical photos of the ECD in the **d** bleached, and **e** colored states. **f** transmittance change at the colored state during open-circuit conditions at 633 nm and in situ transmittance response of the ECD. **g** Transmittance spectra of the ECD in colored/bleached state and colored memory state after 6 h. **h** The optical photo of the ECD in colored memory state after 6 h. **i** Cycling performance of the ECD. ECD, electrochromic device; FTO, fluorine-doped tin oxide.

We characterized the structural and optical performance of the inkjet-printed WO_3_-based large-area ECDs. X-ray diffraction (XRD) patterns of the WO_3_ film on FTO glass substrate reveal diffraction features consistent with orthorhombic crystal structure (JCPDS card No. 43-0679), in addition to peaks from the FTO substrate (Fig. 2a). It’s consistent with previous studies using the same synthesis method for tungsten oxide^31,32^. Fig. 2b displays the surface morphology of the WO_3_ film, revealing WO_3_ nanoparticles (50–200 nm) distributed across the substrate with nanopores. A cross-sectional SEM image (Supplementary Fig. 2) further confirms the WO_3_ film thickness of approximately 240 nm. Device-scale uniformity was evaluated by measuring transmittance at three locations from the center to the edge of a 30 × 30 cm^2^ ECD in both bleached and colored states (points 1-3, Fig. 2c–e). The nearly overlapping spectra at these points demonstrate uniform optical performance across the entire device area, which was reproducible across additional parallel devices (Supplementary Fig. 3). The colored/bleached states of ECD were achieved by applying −3.5 V for 70 s and 2 V for 200 s, respectively, demonstrating a large optical modulation (51% @ 633 nm). The ECD exhibits stable optical memory effect, as shown in Fig. 2f and 2g. After 6 h (21600 s) under open-circuit conditions, i.e., with the device electrically isolated and no external bias or current applied, the colored-state transmittance increases by <11% at 633 nm and the device retains a distinct blue appearance (Fig. 2h). The dynamic optical response at 633 nm is also shown in Fig. 2f, where the 30 × 30 cm^2^ ECD exhibits rapid and reversible switching behaviors by applying −3.5 V for 70 s and 2 V for 200 s, with coloring/bleaching response times of 45.6 s and 47.4 s, respectively. The optical stability of the ECD was also investigated, showing 82.5 % retention of the initial optical contrast after 1000 cycles (270000 s). A slight decrease in the optical contrast in the first 400 cycles, followed by an increase in later cycles, aligns with the activation behavior reported in previous studies^33,34^. Comparison of the initial and after-cycling transmittance–time responses (Supplementary Fig. 4) further indicates that the degradation is mainly associated with a decrease in bleached-state transmittance, whereas the colored-state transmittance changes less markedly. This behavior is consistent with limited charge reversibility and irreversible ion intercalation at the bare FTO counter side during repeated bleaching/coloration processes, as reported for bare FTO/ITO-based counter electrode configurations^35,36^. Importantly, this measurement was performed on a 30 × 30 cm^2^ large-area device, where ion-transport distance, electric-field uniformity, and mechanical stress accumulation pose substantially greater challenges than in small-sized ECDs. Cycling durability at this scale is inherently more demanding, underscoring the practical robustness and application relevance of our ECD design. Additionally, outdoor durability was assessed by continuous exposure in Singapore over 2 months (October and November 2024) (Supplementary Fig. 5). Comparison of the initial and aged devices shows that the decrease in optical contrast mainly originates from a reduced bleached-state transmittance, while the colored-state transmittance remains largely unchanged. After outdoor aging, the ECD maintains a large optical contrast of 38.5% at 633 nm (under −3.5 V for 70 s and 2 V for 200 s), corresponding to 75.6% retention of initial optical contrast. The reduced contrast mainly arises from a lowered bleached-state transmittance, consistent with device aging under prolonged sunlight exposure, while switching kinetics remain comparable (52.1 s for coloring and 41.5 s for bleaching), indicating retained electrochromic functionality after 2 months of tropical outdoor exposure.

### Optimized light regulation and passive cooling in field conditions with EC shading

**Fig. 3:**
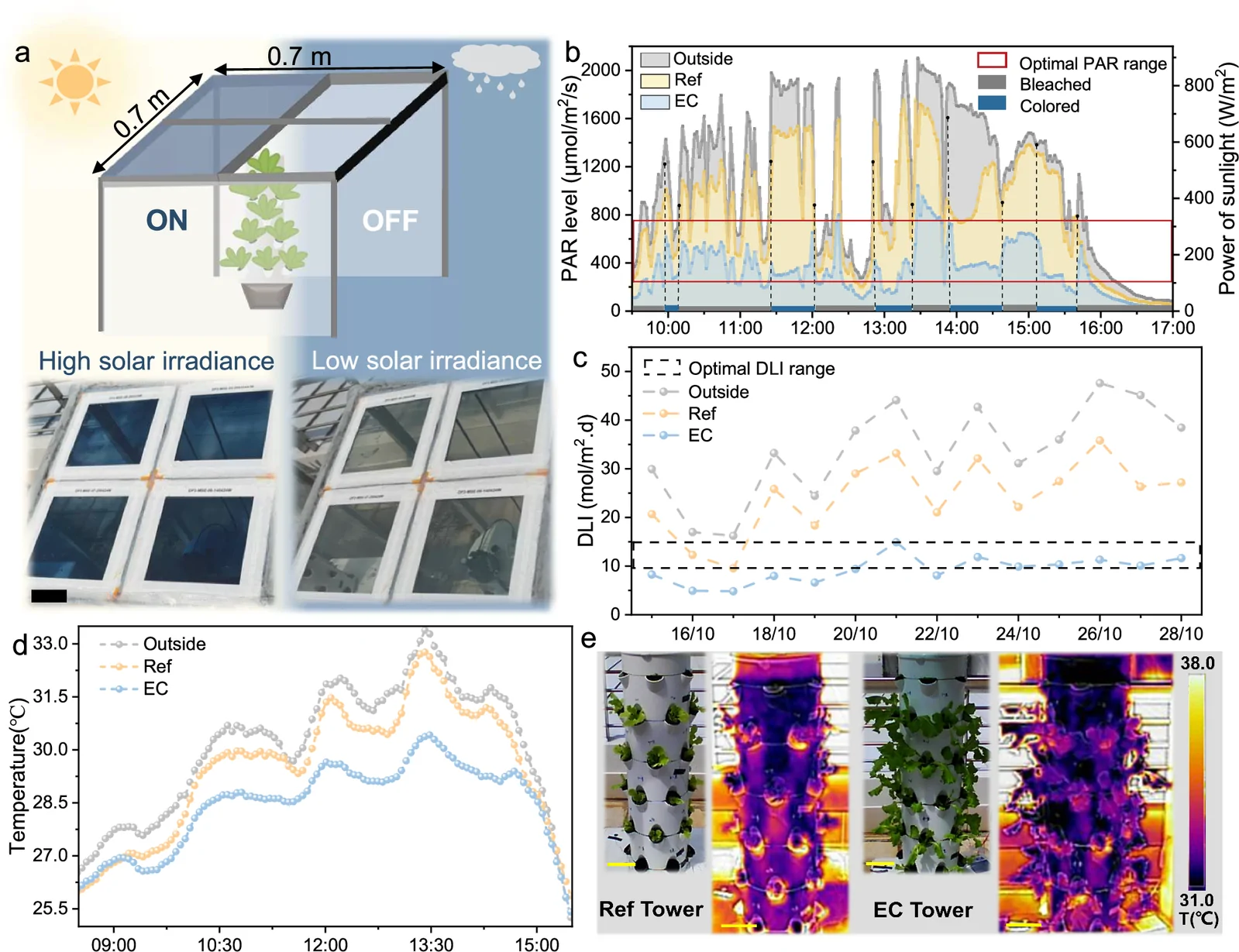
Light and temperature performances of the testbed with EC and Ref shading. **a** Photograph of the meter-scale EC and Ref testbeds with ECDs and PE film as roof shading under high and low solar irradiance conditions. Scale bar, 10 cm. **b** Temporal variation of PAR in the EC and Ref testbeds and unshaded outside environment over a representative growing day (9:00–17:00, October 24, 2024), together with the corresponding outdoor solar power. The bottom bars indicate the bleached and colored states of the EC shading. The boxed region indicates the optimal PAR range for lettuce growth (250–750 µmol/m²/s). **c** DLI values of the EC and Ref testbeds and unshaded outside environment during the plant cultivation period (October 15–28, 2024). The dashed lines indicate the recommended DLI range for lettuce growth (10–15 mol/(m²·d)). **d** Comparison of air temperature in the EC and Ref testbeds and outside environment recorded every 5 min from 7:30 to 18:00 on October 20. **e** Visible and infrared thermal images of the EC and Ref towers with growing lettuce on October 23 at 11:30 am. Scale bar, 10 cm. PAR, photosynthetically active radiation; DLI, daily light integral; ECD, electrochromic device; PE, polyethylene; EC shading, electrochromic shading; Ref shading, reference polyethylene-film shading.

Leveraging the satisfactory electrochromic performance of these scalable and parallel ECDs, we deployed them as EC shading in a meter-scale (0.7 × 0.7 m^2^, 2 × 2 array of 30 × 30 cm^2^ ECD units mounted on a supporting frame) plant-growing testbed for outdoor field experiments in Singapore. A commercial transparent PE film was used as the shading for the reference (Ref) testbed. In the EC testbed, the ECD optical state was autonomously controlled by a solar-powered sensing and control unit to maintain PAR within the optimal range (250–750 µmol/m²/s) for lettuce (*Green Span*) growth^37,38^. For the pilot system, the real-time switching was triggered by a light sensor (lux) positioned under the EC shading, with corresponding EC PAR levels and outdoor PAR threshold levels summarized in Supplementary Table 4. Specifically, the controller implements a dual-threshold strategy: the ECD switches from bleached to the colored state when the PAR intensity beneath the EC shading exceeds ~750 µmol/m²/s (corresponding to ~1240 µmol/m²/s outdoor PAR level); and reverts to the bleached state when PAR intensity beneath the EC shading falls below ~250 µmol/m²/s (corresponding to ~890 µmol/m²/s outdoor PAR) (Fig. 3a). This control strategy maintains transmitted PAR within the target window under fluctuating outdoor irradiance.

Under this control strategy, the temporal variation of PAR in the EC testbed was monitored and compared with the Ref testbed and unshaded outside environment over a representative growing day (Fig. 3b). Switching events are indicated with gray and blue bars, representing the bleached and colored states, respectively. Most switching events closely align with the intended switching thresholds based on the preset outside PAR value. Occasional delayed coloration events (e.g., at 13:50, 15:06) resulted from a built-in 30 min rest period that suppresses rapid oscillatory switching during highly variable irradiance, improving operational stability. Despite this hysteresis behavior, the control strategy effectively maintains PAR in the EC testbed largely within the optimal range for lettuce growth (250–750 µmol/m²/s). By contrast, PAR levels in the Ref testbed and the outdoor environment frequently exceeded the lettuce light-saturation threshold (750 µmol/m²/s). EC shading also maintained the daily light integral (DLI) within a more favorable range for lettuce growth, which represents the total amount of PAR received per day and is a key determinant of plant growth and productivity. On the representative day shown in Fig. 3b (24 October 2024), the DLI under EC shading was 10.0 mol/(m²·d), much closer to the recommended range of 10–15 mol/(m²·d) for lettuce growth than that in the Ref testbed (22.1 mol/(m²·d)) or the unshaded outside environment (31.1 mol/(m²·d))^39^. During the growing period (October 15–28, 2024), the DLI under EC shading reached the recommended range on several test days, while remaining overall much closer to this range than the Ref testbed and the unshaded outside environment, both of which consistently experienced excessive light exposure. Overall, EC shading maintained light conditions that were substantially more favorable than those in the Ref and outside conditions, as reflected by both DLI and PAR values (Fig. 3c and Supplementary Fig. 6).

Beyond light regulation, EC shading reduced the thermal load within the testbed. During peak solar irradiance hours, the EC testbed reached 30.3 °C, representing maximum reductions of 3.1 °C relative to the unshaded ambient condition (33.4 °C) and 2.5 °C relative to the Ref testbed (32.8°C) (Fig. 3d). Infrared thermal imaging showed an overall lower temperature distribution across the EC tower than the Ref tower under the same temperature scale (Fig. 3e). Point measurements at equivalent mid-height positions further indicated ~3 °C lower crop-surrounding air temperature in the EC tower (Supplementary Fig. 7). Over the full growing period, the EC testbed exhibited temperature reductions of 1.2–3.8 °C relative to Ref testbed and outdoor conditions (Supplementary Fig. 8). Maintaining PAR within this window is expected to mitigate excess-light stress while avoiding light limitation, thereby supporting stable photosynthetic performance under outdoor variability.

### Improved crop performance under electrochromic shading

**Fig. 4:**
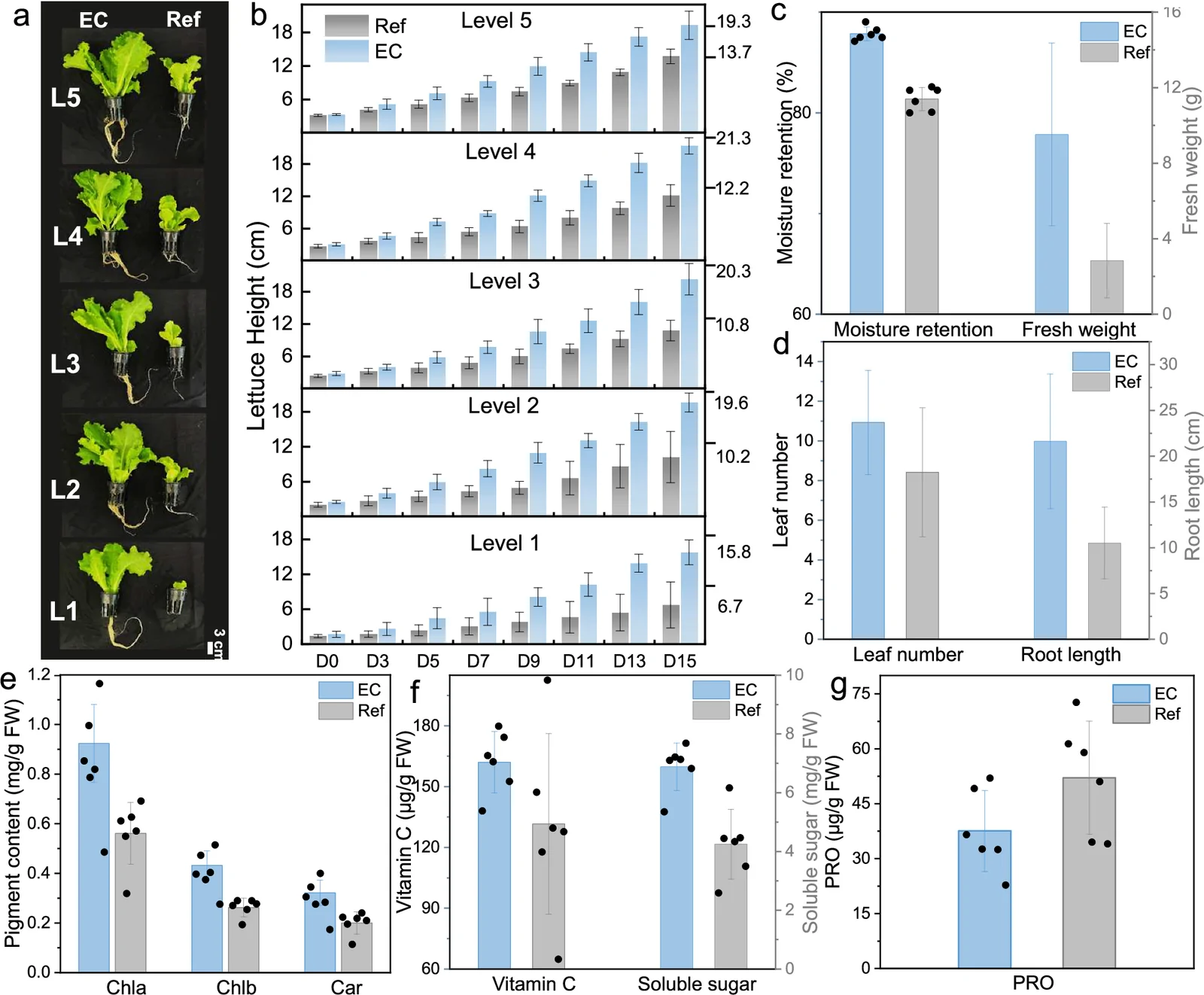
Effects of EC shading on crop cultivation. **a** Photograph of harvested lettuces in different levels of the EC and Ref towers. Scale bar, 3 cm. **b** Height variations of the lettuces in the EC and Ref towers during the growing period. Data are presented as the mean ± s.d. (*n* = 6); error bars represent standard deviations. **c** Moisture retention and fresh weight of harvested lettuces in the EC and Ref towers. Data are presented as the mean ± s.d. (*n* = 6 for moisture retention, *n* = 30 for fresh weight); error bars represent standard deviations. Individual data points are shown for moisture retention measurements. **d** Leaf number and root length of harvested lettuces in the EC and Ref towers. Data are presented as the mean ± s.d. (*n* = 30); error bars represent standard deviations. **e** Pigment contents of harvested lettuces in the EC and Ref towers. **f** Vitamin C and soluble sugar contents of harvested lettuces in the EC and Ref towers. **g** Proline contents of harvested lettuces in the EC and Ref towers. In (**e**–**g**), data are presented as the mean ± s.d. (*n* = 6); the error bars represent standard deviations. Individual data points are shown. EC shading, electrochromic shading; Ref shading, reference polyethylene-film shading; Chl a, chlorophyll a; Chl b, chlorophyll b; Car, carotenoids; PRO, proline.

Following the validation of the EC shading system’s cooling and light-regulation capabilities, its impact on crop cultivation was further evaluated by monitoring the growth performance of hydroponically grown lettuce (Green Span) in vertical towers (five levels, six plants per level) under EC versus Ref shading in Singapore (1.35°N, 103.68°E) from October 15–28, 2024. Lettuce was chosen due to its short growth cycle and sensitivity to heat and light^30,40^. Time-lapse photographs during growth (Supplementary Fig. 9) and at harvest (Fig. 4a), along with height measurements (Fig. 4b), show consistently faster development under EC shading, with an overall height increase of 79.6% relative to the Ref shading across tower levels. Reduced growth at level 1 in both towers is attributed to self-shading from upper layers, which led to insufficient light intensity and a warmer near-ground microenvironment (Supplementary Fig. 10).

Yield metrics further demonstrate the cultivation benefit of EC shading. Lettuce fresh weight (FW) increased by up to 235% under EC shading compared with Ref shading (Fig. 4c). Moreover, moisture content of the crop, a temperature-sensitive parameter^41^, was higher under EC shading (87.8%) compared to Ref shading (81.4%) (Fig. 4c), consistent with a cooler microclimate and reduced evaporative demand. The leaf number (L_N_), which is positively correlated with the phytochrome photostationary state (PSS)^42^, also increased under EC shading (11 leaves) compared to Ref shading (8.4 leaves), representing a 31.0% increase (Fig. 4d). This is attributed to reduced far-red light and lower temperatures under EC shading, which elevated PSS and encouraged leaf development^43,44^. In contrast, the excessive light intensity under Ref shading likely caused photoinhibition, resulting in fewer leaves due to tissue protection and water conservation mechanisms^45^. Additionally, moderate light-temperature conditions under EC shading supported more robust root development, as reflected in longer root length of EC-grown lettuces(Fig. 4d), consistent with enhanced photosynthesis and nutrient uptake^46,47^.

Lettuce, a representative leafy vegetable, is rich in nutrients such as soluble sugars, vitamins, and pigments, all of which are sensitive to environmental conditions. Under EC shading, the pigment contents, including chlorophyll a (Chl a), chlorophyll b (Chl b), and carotenoid (Car), were 60-64% higher than those observed under Ref shading (Fig. 4e), indicating enhanced photosynthetic capacity and improved nutritional accumulation in the leaves^48^. These values fall within the range reported in the literature^49,50^. The observed increase is attributed to the mitigation of heat and light stress under EC shading, which helps preserve photosynthetic pigment levels^51–53^, as well as the enhanced blue light ratio, which has been shown to promote chlorophyll biosynthesis^54^. Furthermore, the contents of soluble sugars and vitamin C—key indicators of nutritional quality and flavor of lettuce^55,56^—were higher under EC shading, with sugar and vitamin C levels reaching 6.88 mg/g FW and 162 μg/g FW, respectively, compared to 4.25 mg/g FW and 131 μg/g FW under Ref shading (Fig. 4f), corresponding to 61.9% and 23.7% increases, respectively. This enhancement is attributed to more favorable PAR intensity and lower temperature provided by EC shading, as excessive PAR (>800 µmol/m²/s) and elevated temperatures (~35 °C) are known to supress sugar and vitamin C accumulation^38,57,58^. Conversely, the stress marker proline, as a multifunctional amino acid, accumulates under high-temperature conditions^58,59^, was 27.9% lower under EC shading compared to Ref shading (Fig. 4g), further confirming the effective cooling capability of the EC system. Together, these results indicate that EC shading improves crop morphology, yield, and nutritional quality in outdoor cultivation by providing a more favorable light and thermal microenvironment.

### Global modeling of light regulation and cooling-energy savings with EC shading

To further evaluate the light-regulation capability and cooling potential of EC shading, a large-scale cultivation model (3.2 m high × 7 m long × 7 m wide) incorporating dynamic EC shading and commercial Ref shading was developed in OpenStudio, and simulated daylighting performance and cooling-energy demand using EnergyPlus (Supplementary Note 1; Supplementary Tables 5–7). Dynamic EC shading was implemented through an energy management system (EMS) using predefined outside incident solar irradiance thresholds on the roof surface (Supplementary Note 1 and Supplementary Tables 6): the EC shading switched to the colored state when outside solar irradiance exceeded 530 W/m^2^ and reverted to the bleached state when outside solar irradiance fell below 380 W/m^2^.

**Fig. 5:**
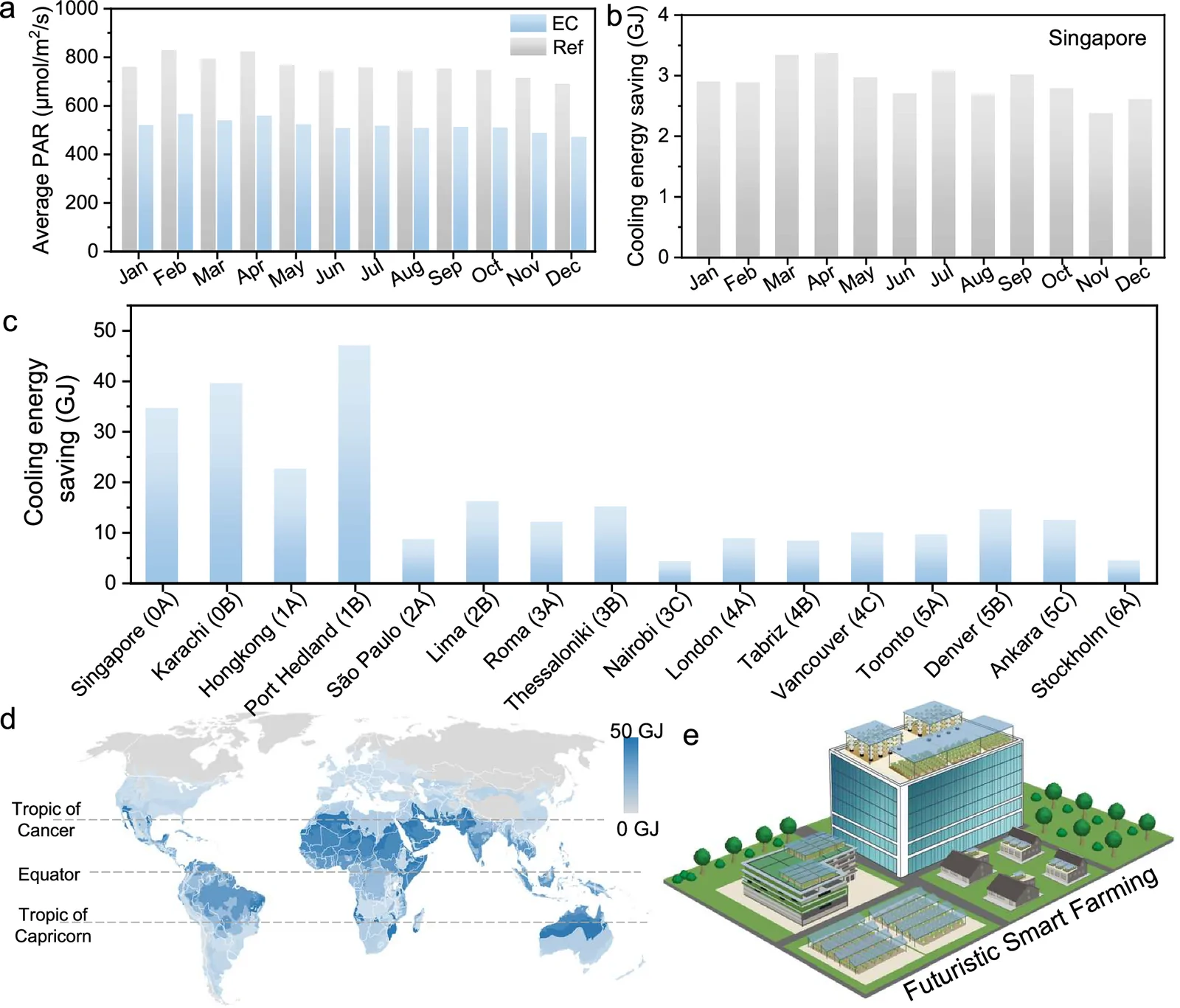
Global EC shading simulation for energy-saving agriculture. **a** The monthly average PAR levels of the cultivation model under EC/Ref shading. **b** The monthly cooling-energy savings of the cultivation model under EC shading compared with the commercial Ref shading in Singapore for the whole year. **c** The global cooling-energy savings of the cultivation model in tropical areas throughout the year and in other regions during the hot season (16 cities across 16 climate zones). **d** The global cooling-energy saving map of the cultivation model under EC shading compared with the commercial Ref shading. **e** The blueprint of a large-scale EC shading application for futuristic smart farming. PAR, photosynthetically active radiation; EC shading, electrochromic shading; Ref shading, reference polyethylene-film shading.

To quantify light regulation within the growing zone, illuminance sampling points were distributed vertically from 0.6 m to 3 m above the floor at 0.16 m intervals (*N* = 16; Supplementary Tables 7). Timestep illuminance (lux) at each point was obtained from EnergyPlus daylighting calculations under Singapore weather conditions and converted to PAR to derive the average crop-relevant light level under EC and Ref shading (Supplementary Note 1). Owing to the irradiance-adaptive switching control, EC shading maintained transmitted PAR within the optimal range for lettuce growth under representative sky conditions (selected from the full-year simulation), including overcast, clear-sky, and cloudy/variable days (Supplementary Fig. 11), whereas the Ref shading frequently resulted in excessive PAR levels. As shown in Fig. 5a, the monthly averaged PAR under EC shading was consistently regulated within the optimal range for lettuce growth (~500 µmol/m²/s), whereas the Ref shading frequently experienced excessive PAR ( ~ 800 µmol/m²/s).

Cooling performance was quantified using an IdealLoads HVAC system with a constant cooling setpoint of 24 °C, and cooling-energy was obtained by aggregating the EnergyPlus output Zone Ideal Loads Supply Air Total Cooling Energy (Supplementary Note 1). As illustrated in Fig. 5b, EC shading reduced the cooling energy demand relative to Ref shading, yielding monthly energy savings ranging from 2.36GJ to 3.35GJ in the tropical climate of Singapore. These reductions arise from the dynamic regulation of transmitted solar heat gain under EC shading, enabling coordinated photothermal modulation that optimizes the crop microclimate while reducing energy demand, thereby supporting scalable and climate-resilient controlled-environment agriculture. To assess the geographical applicability of EC shading’s cooling potential under hot climates, we extended the simulation to 16 representative global cities spanning climate zones 0 A to 6 A using location-specific weather data. For equatorial and tropical hot-climate zones (0A–1B; 0 A/0B/1 A/1B), which experience persistently intense solar irradiance and high ambient temperatures across much of the year^60^, cooling-energy savings were evaluated on a year-round basis. In these regions, EC shading yields the largest absolute savings compared with the Ref shading, reaching 46.91GJ in Port Hedland (1B), 39.34 GJ in Karachi (0B), 34.49 GJ in Singapore (0 A) and 22.44 GJ in Hong Kong (1 A) (Fig. 5c). For climate zones with distinct hot/cold seasons (2A-6A), values in Fig.5c correspond to the hot-season totals (June to August for Northern Hemisphere cities; December to February for Southern Hemisphere cities) to quantify cooling-energy saving performance. Under this seasonal evaluation, intermediate savings are obtained in warm-to-hot locations such as Lima (2B, 16.02 GJ) and Thessaloniki (3B, 14.99 GJ), whereas smaller savings occur in milder or cooler climates with lower cooling demand, including Nairobi (3 C, 4.19 GJ) and Stockholm (6 A, 4.38 GJ). Overall, the geographic variation in cooling-energy savings is consistent with differences in cooling loads driven by ambient temperature and solar heat-gain potential: locations with hotter conditions and higher irradiance exhibit larger absolute cooling-energy savings under EC shading. Cooling-energy savings remain consistently positive across all evaluated locations within the scope of the hot-season analysis.

To visualize the broader geographic implications, the cooling-energy savings associated with each representative city were extrapolated to all global locations sharing the same climate-zone classification (Fig. 5d). This zone-based extrapolation converts the discrete results in Fig. 5c into continuous geographic patterns and highlights where EC shading is expected to deliver the greatest cooling-energy benefits. Cooling-energy savings hot spots emerge across large parts of the tropics and subtropics, as well as hot arid and semi-arid regions, most notably the Middle East and North Africa, consistent with their high solar heat-gain potential and substantial cooling demand. In contrast, temperate and cold regions exhibit weaker savings, reflecting lower cooling-energy demand. Importantly, the consistent cooling-energy reductions observed across all simulated zones indicate that the irradiance-adaptive EC shading can be integrated into a wide range of greenhouse-type or farm cultivation infrastructures, supporting energy-efficient, climate-adaptive crop production. This scalability and geographic robustness highlight EC shading as a promising platform for futuristic smart farming, enabling responsive microclimate control on existing greenhouse cultivation structures (Fig. 5e).

## Discussion

Overall, we developed a high-performance ECD that functions as the core building block of a scalable, irradiance-adaptive EC shading system for outdoor or semi-covered cultivation systems under hot climates. The 30 × 30 cm^2^ ECD demonstrated a large optical contrast (51% at 633 nm), fast switching speeds, stable optical memory effect, and maintained stable electrochromic performance over a 2-month outdoor exposure test under tropical conditions. Building upon this device-level robustness, the integrated EC shading system autonomously switches between bleached and colored states in response to real-time solar irradiance, enabling coordinated photothermal regulation by dynamically tuning PAR transmission while maintaining suppressed UV transmission and substrate-dominated NIR attenuation to reduce radiative heat gain. Notably, this work moves EC technologies beyond laboratory demonstrations by establishing a scalable, field-deployable smart-shading platform for practical outdoor farm cultivation system.

Field deployment in a tropical controlled-environment agriculture confirms that EC shading simultaneously improves the light and thermal microclimate for crops. The system reduced crop-surrounding temperature by ~3 °C while maintaining PAR intensity and DLI within optimal ranges for leafy vegetable cultivation. These photothermal benefits translated directly into agronomic gains, including substantially enhanced nutritional quality (23.7–64% increases in key nutrient contents) and a 235% increase in biomass yield relative to conventional shading films. Benchmarking against emerging agricultural photo-thermal regulation approaches, such as radiative cooling films, photoselective and NIR-reflective covers, and smart glass (Supplementary Table 8), highlights a key advantage of EC shading: dynamic and autonomous co-regulation of both solar-spectrum transmission and heat load, rather than static filtering or single-function cooling.

Beyond site-specific testing, cultivation modeling demonstrates that the EC shading strategy is scalable and geographically adaptable. Simulations across 16 climate zones show consistent reductions in cooling-energy demand during hot seasons, with annual savings reaching up to 46.91 GJ in tropical climates. Together with the field results, these findings position EC shading as a broadly applicable platform for weather-adaptive crop production under hot climates, bridging materials engineering, system-level control, and agricultural microclimate design. Notably, in the present architecture, UV attenuation is primarily determined by the passive filtering of the FTO-glass substrates (Supplementary Fig. 1), while NIR transmission remains relatively low in both states due to the substrate baseline, with only limited electrochromic modulation. This results in persistently reduced NIR transmission in both bleached/colored states. However, when extending the analysis to year-round operation in climate zones with distinct heating seasons (2A–6 A), the same irradiance-triggered control can incur a heating penalty due to NIR attenuation, which reduces passive solar warming and may increase heating energy demand. As summarized in Supplementary Fig. 12, this heating penalty can substantially reduce and even negate (in cold climate zones) the net annual energy benefit. These results reinforce that the present system is primarily suited for cooling-dominated operation (hot climates and hot seasons), and motivate season-aware control and device designs as key future directions.

Building on the above scope and limitation, several directions can further advance this technology. First, future device architectures can incorporate ion-storage electrode materials with electrochemical characteristics appropriately matched to WO_3_, improving charge balance, reducing operating voltage, and further enhancing the long-term stability of large-area EC devices while remaining compatible with large-area and low-temperature fabrication. Second, extending toward truly all-season operation will require season-aware control and device designs that retain high NIR transmittance in the bleached state for passive warming in cold seasons, which will place stricter demands on device architecture and scalable NIR-transparent conductive materials. Third, tailoring electrochromic films for crop-specific spectral modulation would enable more precise control of light intensity and wavelength ratios to match diverse photosynthetic requirements, for example, by designing band-selective electrochromic responses that minimize absorption in crop-beneficial blue/red regions while tuning green transmission and overall PAR to better align spectral composition with crop physiological needs. Fourth, device and system designs could incorporate additional thermal-management functionality, such as mid-infrared radiative control, to further optimize enclosed growing environments. Finally, extended, multi-site field trials at larger scales will be essential to validate long-term reliability, quantify seasonal performance, and accelerate translation toward commercial deployment.

In summary, this work establishes a practical and scalable electrochromic shading platform for adaptive photothermal regulation in real-world agricultural systems under hot climates. By coupling robust large-area ECDs with autonomous system-level control and field-validated crop benefits, EC shading provides a viable pathway toward climate-resilient outdoor farm or greenhouse-type cultivation system.

## Methods

### Materials

Tungsten powder (W, ≥99%), hydrogen peroxide (H_2_O_2_, 30%), ethylene glycol (EG, ≥99.5%), diethylene glycol n-butyl ether (DB, ≥99%), lithium perchlorate (LiClO_4_, ≥95.0%), propylene carbonate (PC, ≥99.7%), ethylene carbonate (anhydrous, 99%), 1-Butyl-3-methylimidazolium bis(trifluoromethyl sulfonyl)imide (BMIMTFSI, ≥98%) were purchased from Sigma-Aldrich. Surfactants were purchased from BYK Additives & Instruments. Polyethylene glycol 1000 (PEG 1000) was purchased from Alfa Aesar. SINGAPORE SAFETY GLASS supplied the FTO glass (30 × 30 cm^2^).

### Fabrication of inkjet-printed 30 × 30 cm^2^ electrochromic device

The WO_3_ nanoparticles were synthesized by reacting W powder with H_2_O_2_ solution under water bath conditions^61^. The resulting pale yellow dispersion was lyophilized and then ground by mortar grinder (Fritsch Mortar Grinder Pulverisette 2). The WO_3_ inkjet printing ink was formulated by dispersing WO_3_ nanoparticles into solvents (EG, DB, and DI water) with the addition of surfactants (BYK). The specific weight percent composition of WO_3_: BYK: EG: DB: DI water = 5: 0.8: 1.7: 31.5: 61. The WO_3_ film was fabricated by printing the WO_3_ ink onto the 30 × 30 cm^2^ FTO-glass substrate using an industry-scale inkjet printer (DOCAN FR2010 Inkjet Printer). The printing resolution and number of printing layers were set as 900 dpi and 3 layers, respectively^62^. After printing, the WO_3_ electrode was dried at 60 °C overnight.

The electrolyte was prepared by dissolving LiClO_4_ salt in organic solvents (ethylene carbonate and PC), with BMIMTFSI and PEG 1000 added to enhance ionic conductivity^63,64^. The concertation of LiClO_4_ salt is 1 mol/L, the weight percent ratio of ethylene carbonate: PC: BMIMTFSI: PEG 1000 = 29.5:59.0:6.3:5.2. The 30 × 30 cm^2^ ECD was assembled by sandwiching a WO_3_ working electrode and a bare FTO-glass counter electrode with the LiClO_4_-based electrolyte, with the help of VHB tape spacer (3 M) and epoxy sealants.

### Design of the testbed with a solar panel-powered and light sensor-controlled system

A light sensor-controlled system was designed by combining a light sensor (BH1750FVI) with an Arduino UNO microcontroller to control the relay on/off under pre-defined light intensity thresholds (lux levels) by detecting the lux level under EC shading every second. This setup enables the automated coloring and bleaching of the ECD by applying the converted DC voltages (−3.5 V/2.0 V). A digital photo and circuit schematic of the system are provided in Supplementary Fig. 13, and a corresponding flowchart with operational parameters (Supplementary Fig. 15 and Supplementary Table 4) is introduced in detail in the Supplementary information.

The solar-powered system consists of a 40 W solar panel (Zhongshan Xinyi Lighting Co., Ltd.; technical specifications provided in Supplementary Fig. 15a) connected to a rechargeable battery (input 5 V, output 12 V 3 A, Zhongshan Xinyi Lighting Co., Ltd.) that consistently stores energy harvest from the panel. The stored energy powers the electronics of the light sensor-controlled system and the ECDs through a voltage conversion module. Experimental measurements showed a maximum current of 0.37 A and total energy consumption of 0.32 W per ECD operation (Supplementary Fig. 15b), confirming that the battery capacity is sufficient to support continuous system operation.

The ECDs were electrically connected via soldered wiring and mounted onto the roof of the EC testbed to serve as the shading. They were automatically triggered by the light-sensing control system and powered by the solar panel. A schematic of the testbed layout is shown in Supplementary Fig. 16. For comparison, the Ref testbed was constructed with a commercial transparent PE film as roof cover. Both EC and Ref testbeds were also covered with PE film on the south/north sides, leaving open spaces for natural ventilation.

### Characterizations of ECD and Measurements of environmental indicators

The optical properties of ECD were measured using a UV–VIS–NIR Spectrometer (Shimadzu UV-3600 spectrophotometer) and a Fourier transform infrared spectrophotometer (Frontier, PerkinElmer) with an Integrating Sphere. The potential applied to ECD was realized by an electrochemical potentiostat (PGSTAT302N, Metrohm Autolab) and a light sensor-controlled box with a rechargeable battery. The crystal structure of the WO_3_ film was characterized by X-ray diffraction (Empyrean XRD Series 2). The morphology of the WO_3_ film was measured by SEM (FEG-SEM, Zeiss Gemini Sigma300). The infrared images of the testbed were taken by the Thermal Imaging Camera (FLIR, EX Series–E4) and analyzed by FLIR Tools software. The temperature of the testbeds and outside was measured and recorded every 5 min using K-type thermocouples with a data logger (MIK-R5000C, Hangzhou Mico Sensing Technology), where the thermocouple was placed in the middle of each testbed tower, and the thermocouple for outside was hung at the same height. The photosynthetically active radiation (PAR) values of the testbeds were monitored and recorded every 2 min using PAR Photon Flux Sensors (Apogee SQ-500, Apogee Instruments, Inc.) connected with a microCache Bluetooth Micro Logger, where the light sensor (BH1750FVI) was placed at the same height on the top of each tower. The daily light integral (DLI, mol/(m²·d)) value is the integration of the PAR value over a day, which is calculated based on the following equation^65^:

1 $${{\rm{DLI}}}={\int }_{0}^{24{hrs}}{{\rm{PAR}}}\left(t\right){dt}$$

### Lettuce cultivation and measurement of physiological and biochemical indicators

Lettuce seeds (Green Span, Arianetech Pte Ltd) were germinated at 22 °C with an 18:6 h light/dark photoperiod and a constant photosynthetic photon flux density by LED lighting. Seeds were sown into sponges and irrigated with a diluted fertilizer solution (Japanese High-Quality A&B Concentrated Liquid Fertilizer, Arianetech Pte Ltd) every 2 days. Twelve days after sowing, seedlings with similar growth status (Supplementary Fig. 17) were transplanted into hydroponic vertical towers (V-gro set, V-Plus Agritech Pte Ltd) of the testbed at the rooftop garden of Materials Science and Engineering, Nanyang Technological University (1.35°N, 103.68°E). The vertical towers were connected to the water tank and pump system, allowing continuous circulation of the nutrient solution from top to bottom to water the lettuce in each holder. Both vertical towers in the EC testbed and Ref testbed were equipped with five levels, each level holding six lettuce seedlings. The specific parameters are shown in Supplementary Table 7. During the cultivation period (October 15 to 28, 2024), the height of each lettuce in the EC and Ref testbeds was recorded every 3 days. Two weeks after transplanting, all lettuces were harvested. The morphological parameters, including leaf number (L_N_), root length, and fresh weight (FW) were measured. Moisture retention was assessed using relative water content (RWC), calculated as RWC% = [(FW-DW)/(TW-DW)] × 100%, where TW is the turgid weight after soaking in DI water for 2 h, and DW is the dry weight after drying at 60 °C for 24 h. Six leaves from each testbed were randomly selected for RWC measurement.

Besides, biochemical indicators of lettuces (including photosynthetic pigments, soluble sugar, vitamin C, and proline) were analyzed using leaves samples at the time of harvest. Twelve leaves were sampled from each EC and Ref testbed, with two 1 × 1 cm segments cut from similar positions on each leaf. Four segments from different leaves were pooled to form one group, resulting in six replicate groups (0.1–0.2 g fresh weight) per testbed. Soluble sugar, vitamin C, and proline contents were quantified using spectrophotometric assay kits (Suzhou Michy Biomedical Technology Co., Ltd) following the instructions. For pigment analysis, each group was ground and extracted with 10 mL of 80% acetone in the dark until the tissue precipitate turned white. The supernatant was collected, and absorbance was measured at 470 nm, 645 nm, and 663 nm. Photosynthetic pigment concentrations (chlorophyll (Chl) a, b, carotenoid (Car)) were calculated using following equations^49^:

2 $${{\rm{Chl\; a}}}({{\rm{mg}}}/{{\rm{g}}})=[(12.72\times {A}_{663}-2.59\times {{{\rm{A}}}}_{645})\times V]/1000W$$

3 $${{\rm{Chl\; b}}}({{\rm{mg}}}/{{\rm{g}}})=[(22.88\times {A}_{645}-4.67\times {A}_{663})\times V]/1000W$$

4 $${{\rm{Car}}}({{\rm{mg}}}/{{\rm{g}}})=[(1000\times {A}_{470}-3.27\times {{\rm{Chl\; a}}}-104\times {{\rm{Chl\; b}}})/229\times V]/1000W$$

where *V* represents the volume of extraction, and *W* is the fresh weight of the tested sample.

### EnergyPlus simulation of the light regulation and cooling effect for electrochromic shading

EnergyPlus, an open-source simulation software (https://energyplus.net/), was used to model a cultivation enclosure with switchable EC roof shading and to quantify its effects on crop-relevant light exposure and cooling-energy demand. A large-scale single-zone cultivation model (3.2 m high × 7 m long × 7 m wide) was constructed in OpenStudio (Supplementary Fig. 18) for two cases: (i) a dynamic EC roof and (ii) a static commercial Ref shading. Simulations were performed using Singapore typical meteorological year weather data at a 10-min timestep (Supplementary Note 1; Supplementary Table 6). The spectral-average optical/thermal properties of the roof shading and wall materials for the EC testbed in its colored/bleached states and the Ref testbed were assigned according to Supplementary Figs. 19–21 and Supplementary Table 5.

Dynamic EC switching was implemented via the EnergyPlus Energy Management System (EMS) using outdoor incident solar irradiance on the roof surface (sensor variable: Surface Outside Face Incident Solar Radiation Rate per Area). The EC roof switched to the colored state when incident irradiance exceeded 530 W/m^2^ and reverted to the bleached state with relevant optical properties when outside incident solar irradiance dropped below 380 W/m^2^, retaining the previous state between thresholds (Supplementary Note 1; Supplementary Table 6).

To evaluate light regulation within the crop-growing region, illuminance sampling points were placed vertically from 0.6 m to 3.0 m above the floor at 0.16 m intervals (*N* = 16; Supplementary Table 7). EnergyPlus daylighting calculations were used to obtain timestep illuminance (lux) at each point, accounting for both transmitted daylight and internal inter-reflections within the enclosure. The zone-mean illuminance was calculated and converted to PAR values (Supplementary Note 1).

Cooling-energy demand was quantified using a ZoneHVAC:IdealLoadsAirSystem with a constant cooling setpoint of 24 °C. From a physical perspective, radiative energy incident on the crop canopy is ultimately partitioned into sensible heat (via convective exchange with the air) and latent heat (via evapotranspiration)^66,67^, which together represent the thermal load that must be removed to maintain the temperature setpoint. In this study, this partition is used as a mechanistic framework for interpreting the cooling demand rather than as an explicitly prescribed latent load. Cooling energy was obtained directly from the EnergyPlus output variable Zone Ideal Loads Supply Air Total Cooling Energy [J] (TimeStep) and aggregated to monthly/annual totals for comparison between EC and Ref cases (Supplementary Note 1). To account for additional effective radiative heat influence from surrounding heated surfaces (e.g., enclosure and ground), a supplementary radiative heat-input term was implemented as an EMS-controlled radiant internal gain, applied identically in both EC and Ref simulations to preserve comparability (Supplementary Note 1).

To explore the geographic applicability, the cultivation model was simulated using typical meteorological year weather data from 16 representative cities spanning climate zones 0A-6A (EnergyPlus official weather database). For equatorial and tropical regions (zones 0A–1B), cooling-energy reductions were evaluated over the full-year, whereas for climate zones 2A–6 A with distinct seasons, results were evaluated over the hot season (June to August for cities in the Northern Hemisphere; December to February for cities in the Southern Hemisphere) to quantify cooling-dominated performance. All model settings (geometry, envelope properties, EMS control, timestep, and HVAC setpoints) were kept identical across locations, with the weather file as the only varying input.

## Supplementary information

Supplementary information

Transparent Peer Review file

## Source data

Source Data

## Data Availability

All data are available in the main text and Supplementary Information. Source data are provided with this paper.
