# Supplementary material for: Scalable irradiance-adaptive electrochromic shading for photothermal regulation
Source: Nat Commun. 2026 Jul 28;17:9126. doi: 10.1038/s41467-026-76115-0 (PMC13518875; doi:10.1038/s41467-026-76115-0)
Supplement: Supplementary file 1 [file 41467_2026_76115_MOESM1_ESM.pdf]

Supporting Information

Scalable irradiance-adaptive electrochromic shading for photothermal regulation

Wenting Wu[‡,1,2], Xueyang Wang[‡,1,2], Alvin Wei Ming Tan[1,2], Yangyang Xin[1,2], Yixuan Jiang[1,2], Tan Hu[1,2], Hui Wang[1,2], Yawei Jiang[1,2], Pooi See Lee*[,1,2]

[1]School of Materials Science and Engineering, Nanyang Technological University, 50 Nanyang Avenue, Singapore 639798, Singapore

[2]Singapore-HUJ Alliance for Research and Enterprise (SHARE), Smart Grippers for Soft Robotics (SGSR), Campus for Research Excellence and Technological Enterprise (CREATE), Singapore 138602, Singapore

*Corresponding author: Pooi See Lee, E-mail: pslee@ntu.edu.sg

[‡]These authors contributed equally

The PDF includes:

1. Supplementary Note 1

Note 1. EnergyPlus simulation of the light regulation and cooling-energy consumption of the cultivation model

S1.1 Model inputs and boundary conditions

S1.2 Dynamic EC roof control in EnergyPlus (EMS)

S1.3 Output 1: Light regulation within the cultivation zone

S1.4 Output 2: Cooling-energy demand and energy-savings calculation

S1.5 Cooling-energy evaluation across global climate zones

2. Supplementary Figs. 1-21

Fig. 1. Bare FTO-glass transmittance vs wavelength and band-integrated Transmittance value

Fig. 2. Cross-section SEM image of the WO_3 film

Fig. 3. The transmittance spectra and digital photos of three parallel 30 × 30 cm^2 ECDs in colored/bleached states from center to side points and memory state after 6 h.

Fig. 4. The transmittance–time response during the initial cycles and after 1000 cycles of the 30cm*30cm ECD

Fig. 5. Comparison of the initial and aged electrochromic device after two months of outdoor exposure in Singapore

Fig. 6. The PAR values of the EC/Reference testbeds and outside environment during the growing period (October 15th - 28th, 2024).

Fig. 7. The IR (a, c) and optical image (b, d) of the Ref and EC tower with growing lettuce on October 23rd at 11:30 am.

Fig. 8. The temperature reduction and corresponding temperature points of the EC testbed compared with the Ref testbed and outside environment during the growing period (October 15th - 28th, 2024).

Fig. 9. The photographs of growing variations of lettuces in the vertical tower under EC and Ref shading throughout the cultivation cycle.

Fig. 10. The air temperature recorded by the thermocouples on the upper/lower location of the EC/Ref towers at the same height on October 25th, 2024.

Fig. 11. Simulated daily PAR profiles under representative sky conditions and corresponding EC switching states.

Fig. 12. Annual cooling-energy saving, heating-energy saving (negative values indicate heating penalty), and net annual energy saving of EC shading relative to the reference roof across climate zones 2A–6A, based on year-round EnergyPlus simulations (heating setpoint: 20 °C; cooling setpoint: 24 °C).

Fig. 13. (a) Digital photo and (b) diagram of the circuit in the light sensor-controlled system.

Fig. 14. The flowchart with the setting parameters of the circuit in the light sensor-controlled system

Fig. 15. a. The solar panel (35 × 63 cm^2) parameters provided by the supplier. **b.** The current-time curves of one ECD during switch.

Fig. 16. The Reference and Electrochromic testbed layout

Fig. 17. The lettuce seedlings with similar growth status before transplant

Fig. 18. A large-scale cultivation model (3.2 m (h) × 7 m (l) × 7 m (w)) with dynamic EC shading/commercial Ref shading

Fig. 19. The solar reflectance spectrum of ECD with colored/bleached states

Fig. 20. The solar reflectance and transmittance spectrum of PE film

Fig. 21. The infrared reflectance and transmittance spectrum of PE film

3. Supplementary Tables 1-9

Table 1. Band-integrated UV/NIR transmittance and blocking

Table 2. The transmitted solar energy irradiation (W/m^2) of ECD with colored/bleached states under different wavebands (UV 280 - 400 nm, VIS 400 - 780 nm, NIR 780 - 2500 nm)

Table 3. The PAR values ($\mu mol/m^2/s$) under different wavebands (blue 400-500 nm, green 500-600 nm and red 600-700 nm)

Table 4. Switching condition parameters of the electrochromic shading system (under-ECD illuminance/PAR/energy irradiance, and corresponding outdoor levels)

Table 5. Optical and thermal properties of the roof shading/wall materials used in the EneryPlus model

Table 6. Geometry, EC control logic, and simulation settings of the cultivation model

Table 7. Illuminance sampling and effective radiative-input parameters

Table 8. Comparison between EC shading with other light-thermal regulation technologies, including radiative cooling, photoselective cover, smart glass cover, and NIR-reflective cover

Table 9. The parameters of the testbed

4. Supplementary References

Supplementary Note

Supplementary Note 1. EnergyPlus simulation of the light regulation and cooling-energy consumption of the cultivation model

S1.1 Model inputs and boundary conditions

A single-zone cultivation model (3.2 m high × 7 m wide × 7 m long) was developed in OpenStudio/EnergyPlus to quantify the influence of dynamic EC roof shading on (i) light regulation within the crop zone and (ii) cooling-energy demand under Singapore's climate. Side walls were modelled using a conventional greenhouse film in both EC and Ref cases. Roof optical properties for the EC (colored/bleached) and Ref roofs, together with side-wall properties, are summarized in Supplementary Table 5. The cultivation region was represented as an effective crop zone (no explicit plant geometry), and no additional mechanical ventilation control was imposed. Infiltration/outdoor-air exchange was treated identically in the EC and Ref simulations; therefore, differences in cooling-energy demand arise from the roof optical properties and EC switching control rather than ventilation settings. Cooling-energy simulations employed a ZoneHVAC:IdealLoadsAirSystem with a constant indoor cooling set point of 24 °C. Simulations used a 10-minute time step, and outputs were reported at the time step resolution.

S1.2 Dynamic EC roof control in EnergyPlus (EMS)

The EC roof shading state was controlled through an Energy Management System (EMS) according to the outdoor incident solar radiation on the roof surface. The EC roof shading switched to the colored state when the incident solar radiation exceeded 530 W/m^2, switched to the bleached state when it fell below 380 W/m^2, and retained its previous state between these thresholds:

$$T_{\mathrm{EC}}(t) = \begin{cases} T_{\mathrm{colored}}, & E_{\mathrm{roof}}(t) > E_{\mathrm{col}} \\ T_{\mathrm{bleached}}, & E_{\mathrm{roof}}(t) < E_{\mathrm{ble}} \\ T_{\mathrm{EC}(t-1)}, & E_{\mathrm{ble}} \le E_{\mathrm{roof}}(t) \le E_{\mathrm{col}} \end{cases} \quad \text{(S1)}$$

where $E_{roof}(t)$ represents the incident solar radiation on the roof surface in W/m^2, E_{col} =530 and E_{ble} = 380 W/m^2, and $T_{colored}$ and $T_{bleached}$ denote the transmittance of the EC roof shading in colored and bleached states, respectively. $T_{EC}(t)$ is not limited to transmittance but also applies to reflectance-related parameters. For the Ref shading case, the roof optical performance remained constant. The same EMS logic was applied in both the daylighting and cooling-energy simulations.

S1.3 Output 1: Light regulation within the cultivation zone

To characterize light regulation within the cultivation zone, illuminance sampling points were distributed vertically from 0.6 m to 3.0 m above the floor at 0.16-m intervals (N = 16; **Supplementary Table 7**). The point height is:

$$z_i = z_1 + \Delta z\,(i-1),\ i=1,\ldots,N \tag{S2}$$

EnergyPlus timestep daylighting calculations were used to obtain illuminance $I_i(t)$ (lux) at each point, which accounts for transmitted visible daylight and internal visible reflection inside the cultivation model. The zone-mean illuminance across the cultivation zone was calculated as:

$$\bar{I}(t) = \frac{1}{N}\sum_{i=1}^{N} I_i(t) \tag{S3}$$

where $\bar{I}(t)$ is the average illuminance within the cultivation zone at time t (lux). For comparison with crop-relevant metrics, illuminance was converted to PAR using a standard conversion factor,[1] and monthly mean PAR values were reported for EC and Ref cases.

S1.4 Output 2: Cooling-energy demand and energy-savings calculation

For the cooling-energy analysis, the transmitted shortwave solar radiation entering the cultivation zone was calculated as:

$$q_{rad}(t) = E_{\mathrm{roof}}(t)A_{\mathrm{roof}}T_{\mathrm{roof}}(t) + \sum_{j=1}^{4} E_{\mathrm{wall},j}(t)A_{\mathrm{wall},j}T_{\mathrm{wall}} \tag{S4}$$

where $q_{rad}(t)$ is the transmitted shortwave solar radiation entering the cultivation model at time t (W), $E_{roof}(t)$ and $E_{wall,j}(t)$ are the incident solar radiation on the roof and wall surfaces (W/m^2), A_{roof} and $A_{wall,j}$ are the corresponding surface areas (m^2), and $T_{roof}(t)$ and T_{wall} is the solar transmittance of the roof shading and side-walls, respectively.

The transmitted shortwave input $q_{\mathrm{rad}}(t)$ in Eq. (S4) serves as the primary radiative input to the cultivation model. In addition, to account for effective radiative heat influence from surrounding surfaces (e.g., enclosure and ground), we introduced a supplementary radiative heat-input term.[2,3] This term was implemented in EnergyPlus as an EMS-controlled radiant internal gain and was applied consistently in both EC and Ref models to preserve comparability. We note that this auxiliary term is introduced as an equivalent radiant load and does not modify the native EnergyPlus surface longwave-exchange algorithm, thereby avoiding double counting with the standard longwave radiation exchange solved internally by EnergyPlus.

In the present model, the supplementary radiative contribution was approximated as a fixed fraction of the total effective heat input:

$$q_{\mathrm{use}}(t) = \frac{q_{\mathrm{rad}}(t)}{1 - f_{\mathrm{th}}} \tag{S5}$$

$$q_{\mathrm{th}}(t) = f_{\mathrm{th}} q_{\mathrm{use}}(t) \tag{S6}$$

where $q_{\mathrm{use}}(t)$ is the total effective radiative energy input after accounting for transmitted shortwave solar radiation and supplementary radiative component (W), $q_{\mathrm{th}}(t)$ is the thermal radiation component (W), and f_{th} is the thermal radiation fraction. The corresponding effective radiative-input parameter is listed in **Supplementary Table 7**.

The total absorbed indoor shortwave radiative gain was written as:

$$q_{\mathrm{sol,tot}}(t) = \sum_s q_{\mathrm{sol},s}(t) \tag{S7}$$

where $q_{\mathrm{sol},s}(t)$ is the absorbed radiative gain at interior surface s. In EnergyPlus, transmitted solar radiation is distributed over the interior surfaces according to the cultivation model geometry, solar optical properties, and solar position, and the absorbed shortwave gain at the interior surface can be represented conceptually as:

$$q_{\mathrm{sol},s}(t) = \Psi_s(t) q_{\mathrm{use}}(t) \tag{S8}$$

where $\Psi_s(t)$ is an effective surface-specific solar-distribution coefficient determined by the standard EnergyPlus solar-distribution routine. From a physical perspective, part of the effective radiative input absorbed by the crop canopy is ultimately partitioned into latent heat associated with evapotranspiration and sensible heat associated with crop-air heat exchange.[4,5] As crop

sensible heat can be one possible pathway through which absorbed radiation is transferred to the indoor air, in the present study, this latent/sensible partition was therefore considered as a mechanistic framework for interpreting how canopy-absorbed radiation contributes to the model thermal regulation rather than as additional prescribed loads, to avoid double counting with thermal gains.

At the indoor surface level, the absorbed solar gains were resolved together with longwave radiation exchange, conduction through the roof shading, and convection with the indoor air through the standard surface heat balance, which can be written as:

$$C_s \frac{dT_s(t)}{dt} = q_{\mathrm{sol},s}(t) + q_{\mathrm{LW},s}(t) + q_{\mathrm{cond},s}(t) - q_{\mathrm{conv},s}(t) \quad \text{(S9)}$$

where C_s is the thermal capacitance of interior surface s, $T_s(t)$ is the inside surface temperature, $q_{\mathrm{sol},s}(t)$ is the absorbed shortwave solar heat gain, $q_{\mathrm{LW},s}(t)$ is the net longwave radiative exchange, $q_{\mathrm{cond},s}(t)$ is the conductive heat transfer through the surface, and $q_{\mathrm{conv},s}(t)$ is the convective heat exchange between surface s and indoor air. Here, $q_{\mathrm{sol},s}(t)$ is determined from the effective radiative input together with the solar optical properties and geometry of the cultivation model. $q_{\mathrm{LW},s}(t)$ is determined by the infrared emissivity properties of the roof shading/side wall materials and the enclosure radiative exchange conditions. $q_{\mathrm{cond},s}(t)$ represents the conductive heat transfer through the roof shading and side wall materials, determined by the material thickness and conductivity. These terms were internally resolved by the standard EnergyPlus surface heat-balance routines and were not prescribed as independent input loads.

The convective term is:

$$q_{\mathrm{conv},s}(t) = h_{\mathrm{c},s}(t) A_s \big(T_s(t) - T_{\mathrm{Z}}(t)\big) \quad \text{(S10)}$$

where $h_{\mathrm{c},s}(t)$ is the inside convective heat-transfer coefficient, A_s is the surface area, and $T_{\mathrm{z}}(t)$ is the zone air temperature.

The zone air temperature was then determined from the zone air heat balance:

$$C_{\mathrm{z}} \frac{dT_{\mathrm{z}}(t)}{dt} = \sum_s q_{\mathrm{conv},s}(t) + m_{\mathrm{inf}}(t) c_p \big(T_{\mathrm{out}}(t) - T_{\mathrm{Z}}(t)\big) + m_{\mathrm{sys}}(t) c_p (T_{\mathrm{sup}}(t) - T_{\mathrm{z}}(t)) + Q_{\mathrm{int.sens}}(t) \quad \text{(S11)}$$

where C_z is the thermal capacitance of the zone air, $m_{inf}(t)$ is the infiltration/outdoor-air exchange rate, $m_{sys}(t)$ is the HVAC supply-air mass flow rate, C_p is specific heat capacity of the zone air, $T_{out}(t)$ is the outdoor air temperature, $T_{sup}(t)$ is the supply-air temperature, and $Q_{int,sens}(t)$ is the net sensible internal gain (including the EMS-controlled supplementary radiant internal gain described above).

Under the ZoneHVAC:IdealLoadsAirSystem assumption, the cooling system provides the ideal sensible and latent removal required to maintain the zone air temperature at the prescribed set point. The instantaneous ideal cooling rate can be expressed as:

$$q_{\text{cool}}(t) = m_{\text{sys}}(t)c_p(T_{\text{z}}(t) - T_{\text{sup}}(t)) \quad \text{(S12)}$$

for the case where $T_z(t)$ exceeds the cooling setpoint and cooling is required.

In the present study, cooling-energy was obtained directly from the EnergyPlus output variable "Zone Ideal Loads Supply Air Total Cooling Energy [J] (TimeStep)", reported at the 10-min timestep resolution over a full year using Singapore's typical meteorological data. Since the EnergyPlus output used here is a timestep cooling-energy quantity, the monthly cooling-energy was obtained by summing the timestep cooling-energy output within each month:

$$E_{\text{cool,m}} = \sum_{n\in m} E_{\text{cool},n} \quad \text{(S13)}$$

and the annual cooling-energy was similarly obtained by summing all timestep cooling-energy values over the full-year:

$$E_{\text{cool,year}} = \sum_{n=1}^{N_{year}} E_{\text{cool},n} \quad \text{(S14)}$$

The monthly cooling-energy saving achieved by the EC roof shading was calculated as:

$$\Delta E_{\text{m}} = E_{\text{cool,m}}^{\text{ref}} - E_{\text{cool,m}}^{\text{EC}} \quad \text{(S15)}$$

Similarly, the annual cooling-energy saving was calculated as:

$$\Delta E_{\text{year}} = E_{\text{cool,year}}^{\text{ref}} - E_{\text{cool,year}}^{\text{EC}} \quad \text{(S16)}$$

where $E_{\text{cool}}^{\text{ref}}$ and $E_{\text{cool}}^{\text{EC}}$ denote the cooling-energy demands of the reference roof shading and EC roof shading, respectively.

S1.5 Cooling-energy evaluation across global climate zones

To assess geographic applicability, the EnergyPlus cultivation model was simulated using typical meteorological year (TMY/IWEC/CWEC) weather files from 16 representative cities spanning climate zones 0A–6A, obtained from the official EnergyPlus weather database. For each city, the same model geometry, material properties, EMS switching logic, timestep (10 min), and HVAC control settings were retained, and only the weather file was changed (Supplementary Table 7). Cooling energy for each city was obtained directly from the EnergyPlus output variable "Zone Ideal Loads Supply Air Total Cooling Energy [J] (TimeStep)", reported at the timestep resolution. For equatorial and tropical regions (climate zones 0A–1B), cooling-energy totals were evaluated over the full year. For cities within climate zones 2A–6A with distinct hot/cold seasons, the analysis focused on the hot seasons (June to August for cities in the Northern Hemisphere; December to February for cities in the Southern Hemisphere) to quantify cooling-energy savings under cooling-dominated conditions, consistent with the intended operating scope of the irradiance-adaptive EC shading.

The seasonal cooling energy was computed by summing timestep cooling-energy outputs over the selected analysis period P:

$$E_{\mathrm{cool},P} = \sum_{n\in P} E_{\mathrm{cool},n} \tag{S17}$$

where Ecool,n is the timestep cooling energy output and P denotes either (i) the full-year period (for zones 0A–1B) or (ii) the June–August period (for cities in the Northern Hemisphere within zones 2A–6A) or December to February period (for cities in the Southern Hemisphere within zones 2A–6A). The corresponding cooling-energy saving was calculated as:

$$\Delta E_P = E_{\mathrm{cool},P}^{\mathrm{ref}} - E_{\mathrm{cool},P}^{\mathrm{EC}} \tag{S18}$$

Note: For climates outside the tropics, we report cooling-energy results for the hot season to quantify cooling-dominated performance; year-round heating and cooling results are provided separately to illustrate potential cold-season heating penalties (Supplementary Fig. 12).

Supplementary Figure

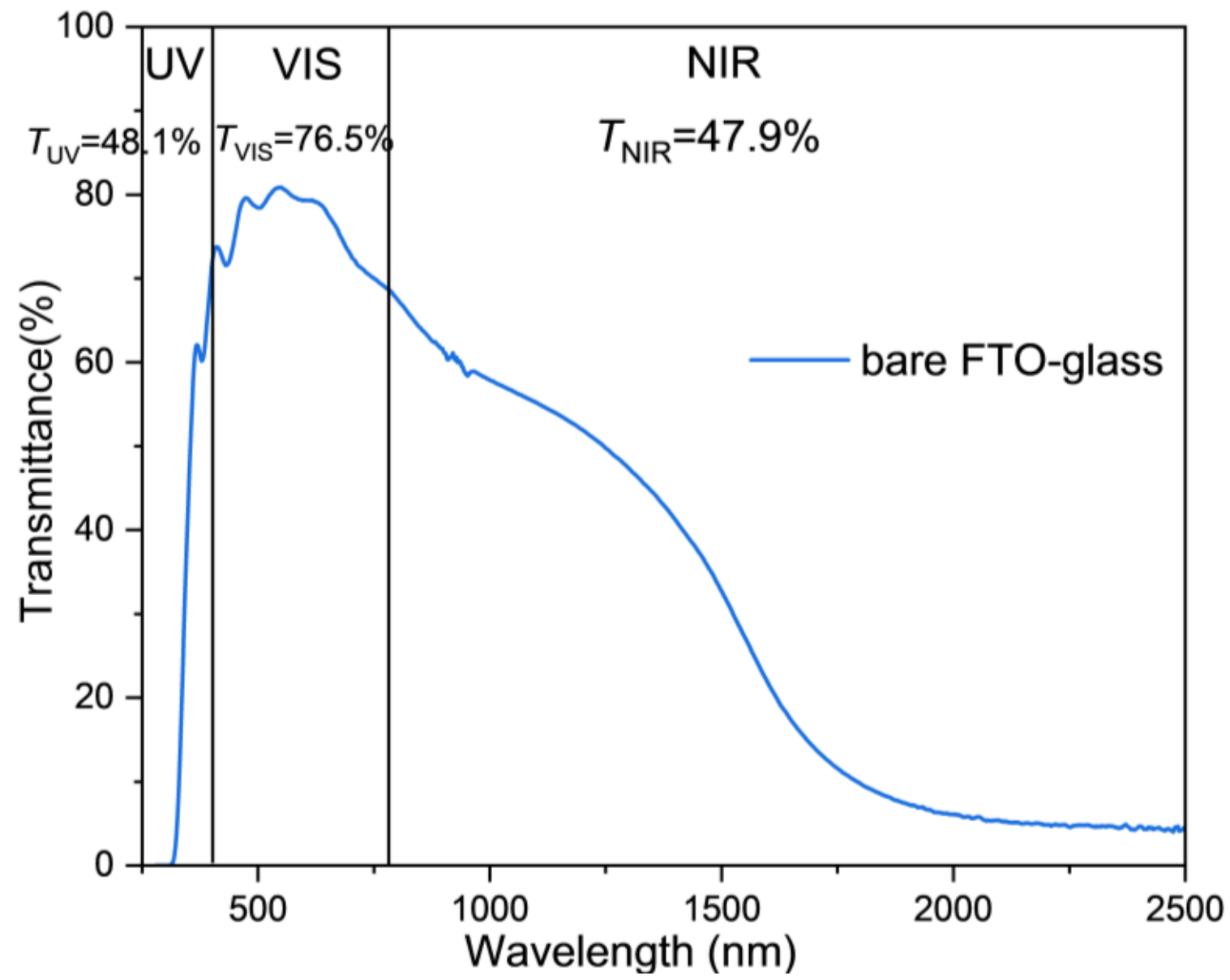

Supplementary Fig. 1. Bare FTO-glass transmittance vs wavelength and band-integrated Transmittance value

The passive filtering function of the bare FTO–glass substrate is quantified by using AM1.5G-weighted, band-integrated transmittance metrics: T_{UV}=48.1%, T_{VIS}=76.5%, T_{NIR}=47.9%. These results confirm that FTO-glass is a non-negligible passive attenuator in both UV and NIR bands.

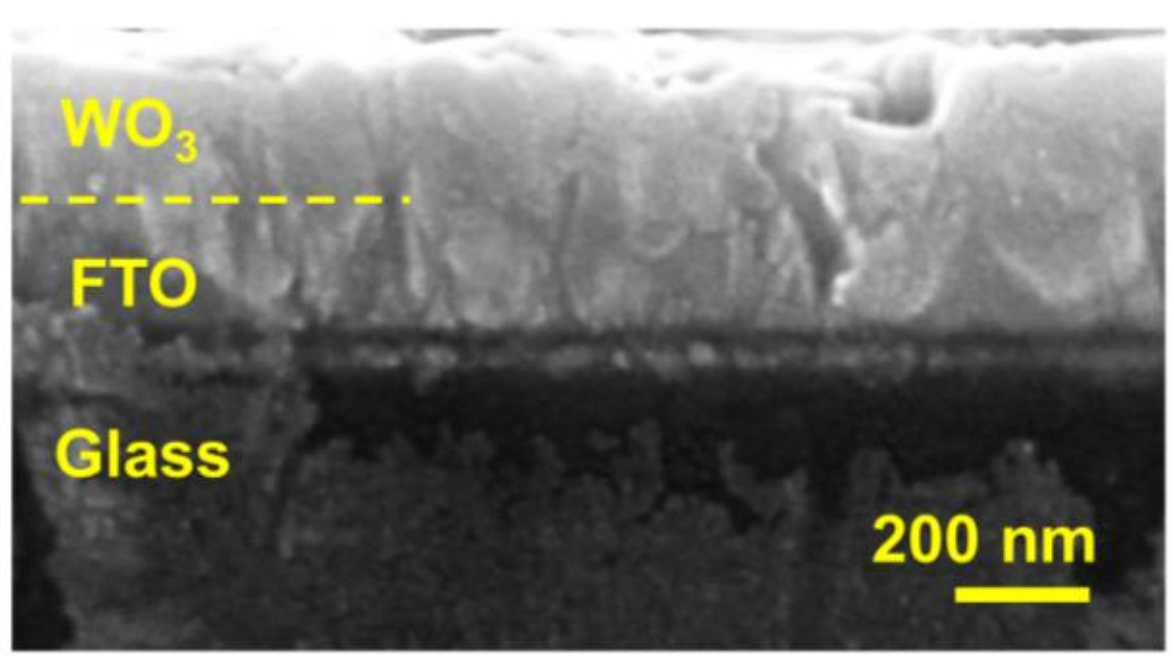

Supplementary Fig. 2. Cross-section SEM image of the WO_3 film

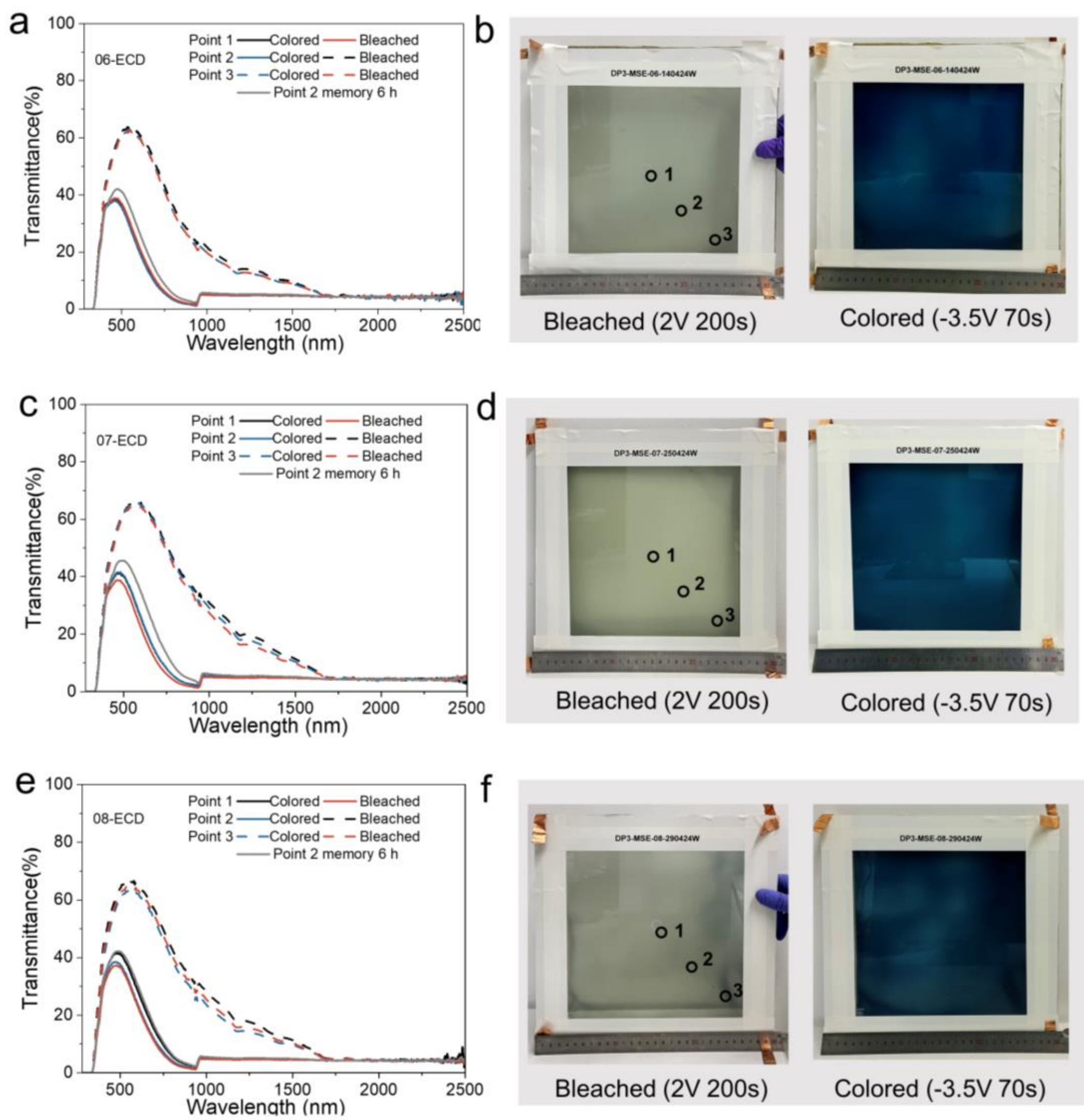

Supplementary Fig. 3. The transmittance spectra and digital photos of three parallel 30 × 30 cm^2 ECDs in colored/bleached states from center to side points and memory state after 6 h.

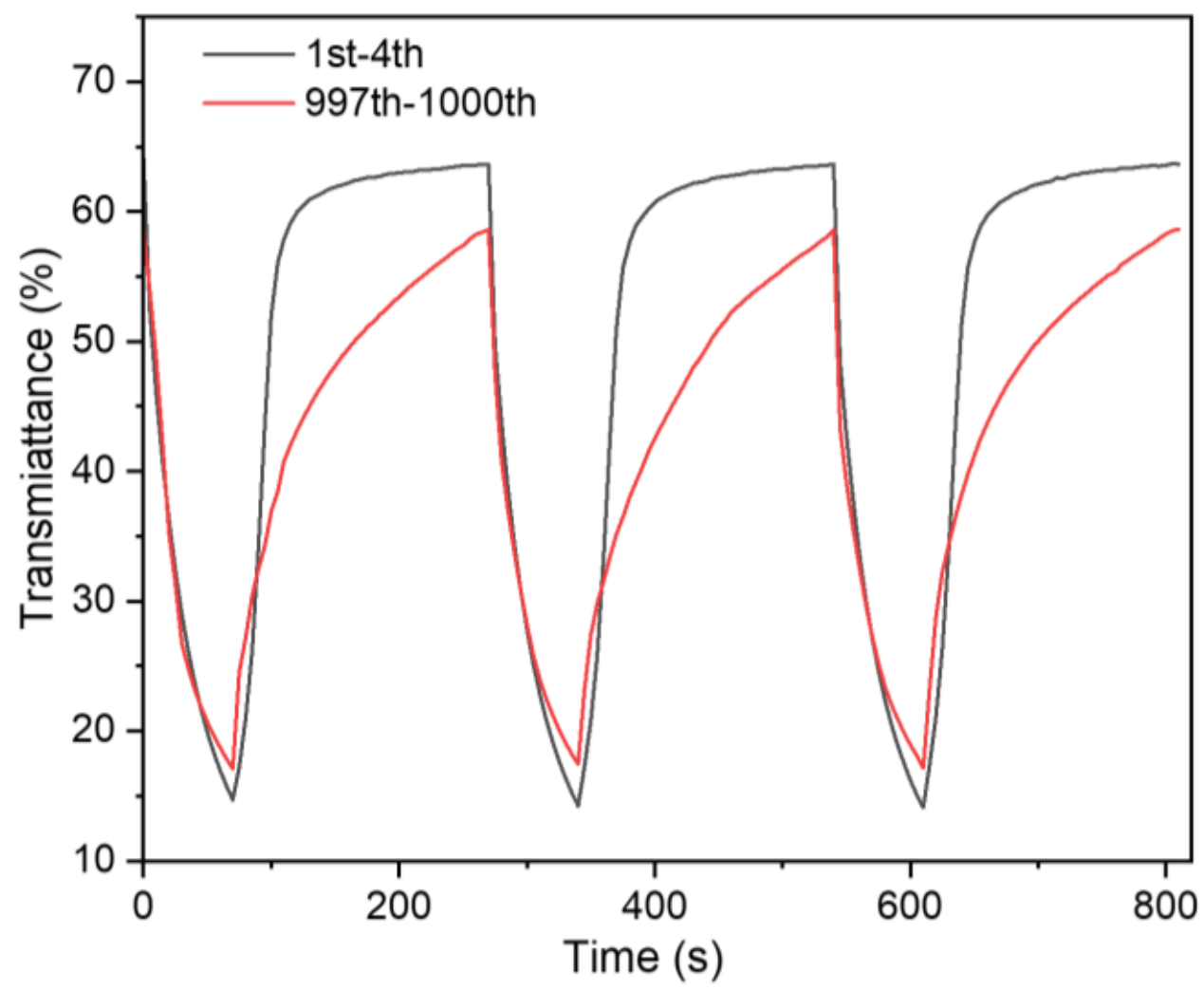

Supplementary Fig. 4. The transmittance–time response during the initial cycles and after 1000 cycles of the 30 × 30 cm^2 ECD

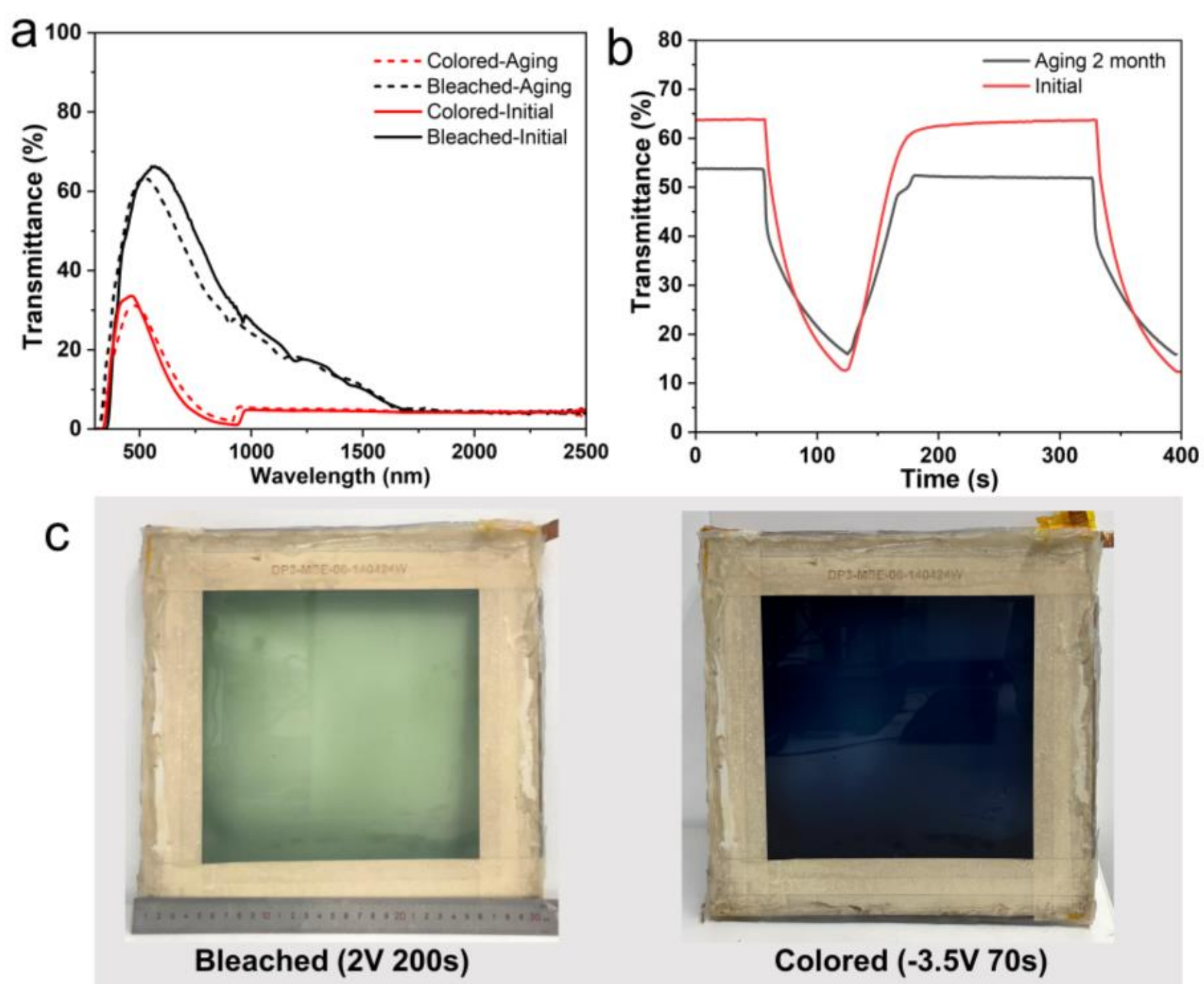

Supplementary Fig. 5. Comparison of the initial and aged electrochromic device after two months of outdoor exposure in Singapore. **a**, Transmittance spectra in the colored and bleached states. **b**, In-situ transmittance response at 633nm and **c** digital photos of 30 × 30 cm^2 ECD in colored/bleached states of the initial device and the device after two months of outdoor aging.

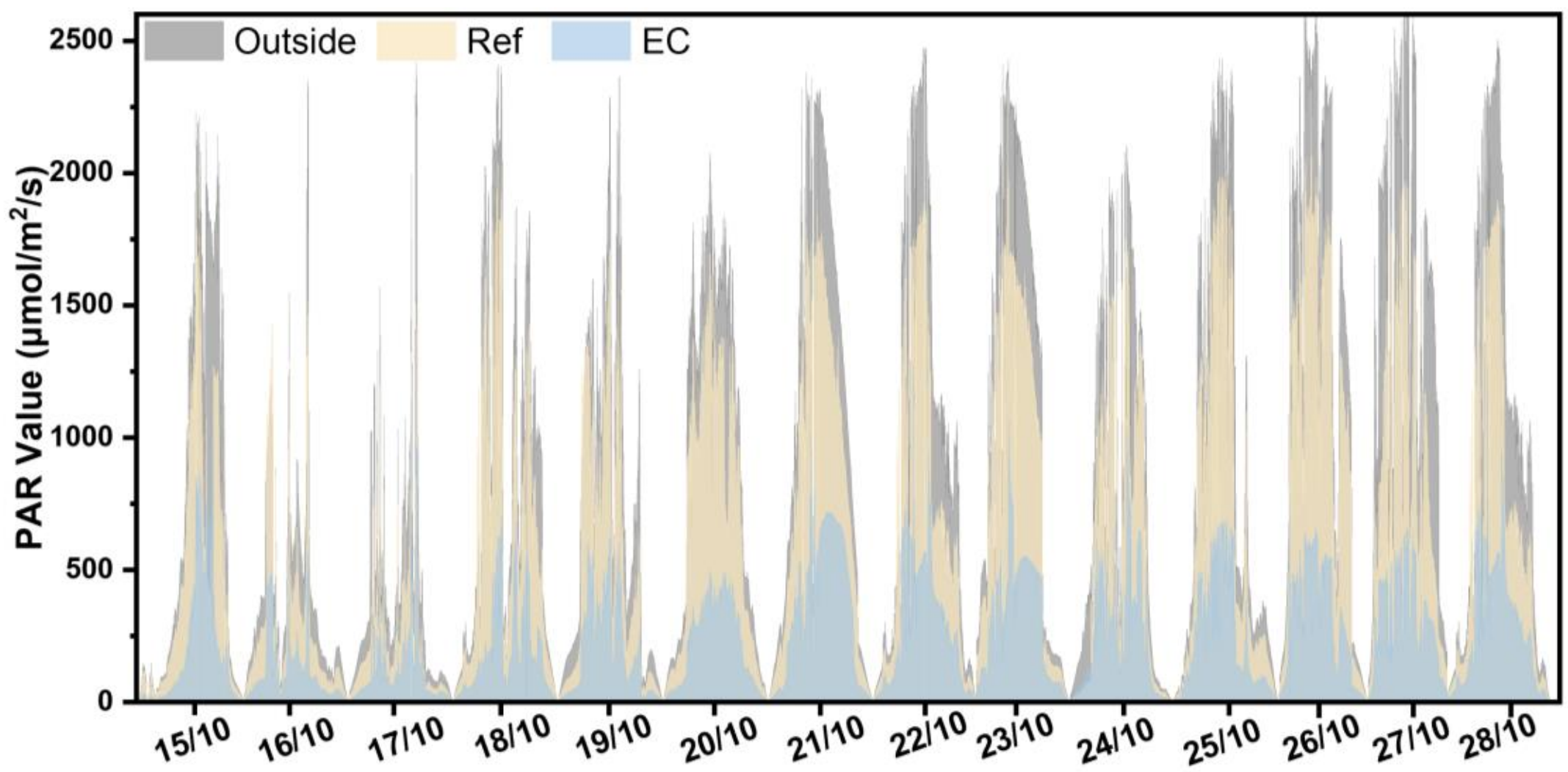

Supplementary Fig. 6. The PAR values of the EC/Reference testbeds and outside environment during the growing period (October 15th - 28th, 2024)

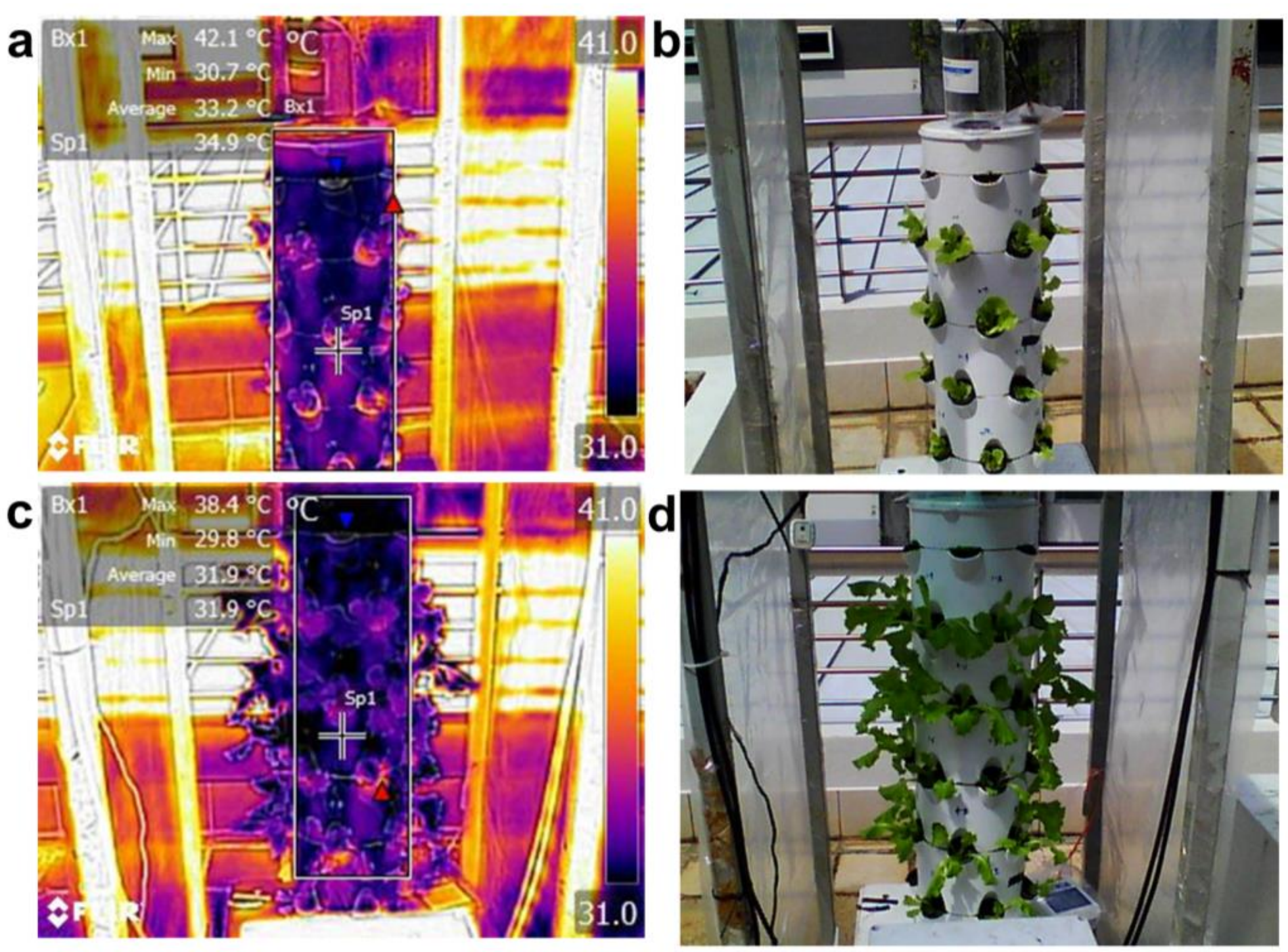

Supplementary Fig. 7. The IR (a, c) and optical image (b, d) of the Ref and EC tower with growing lettuce on October 23rd at 11:30 am.

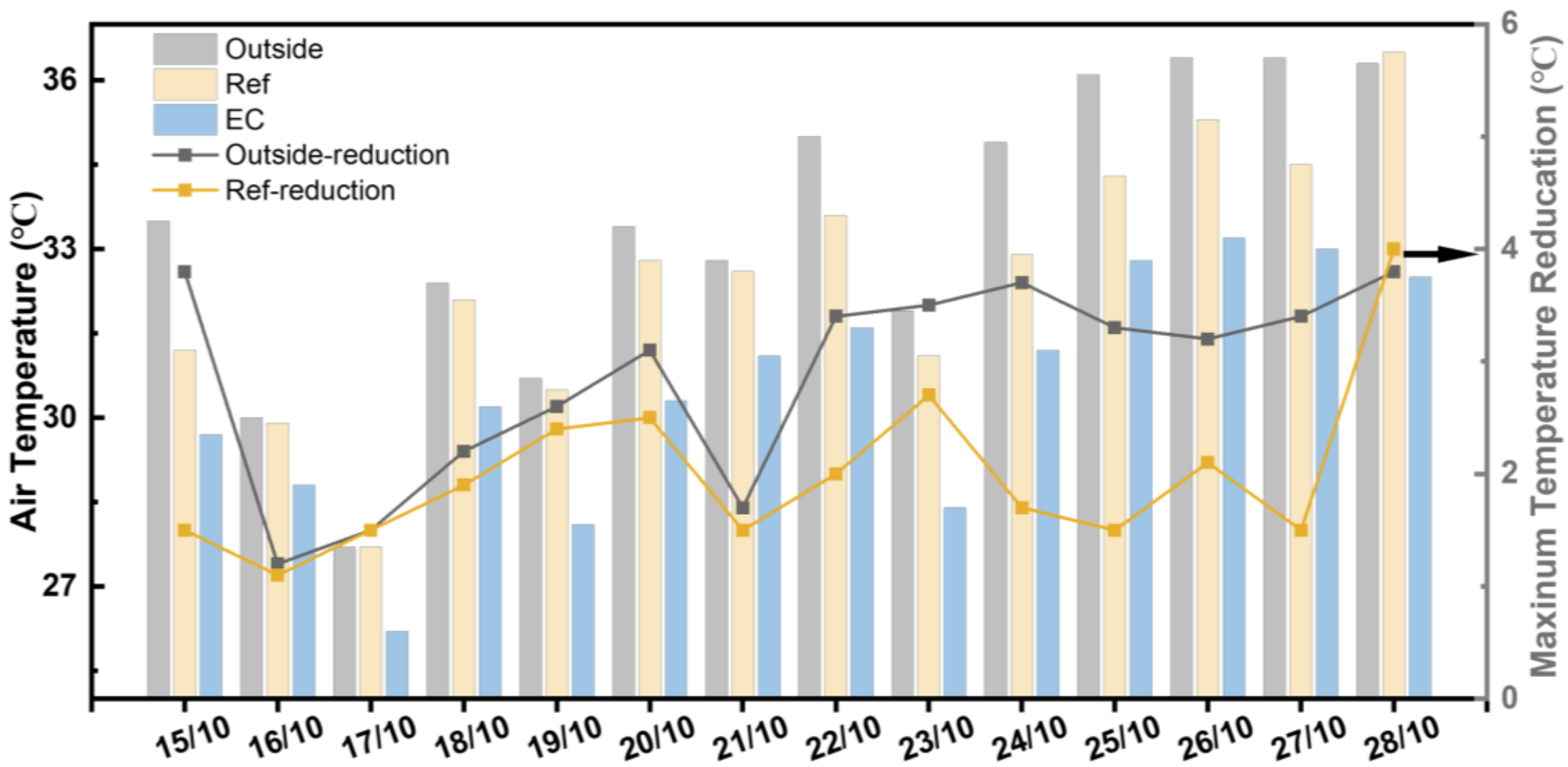

Supplementary Fig. 8. The temperature reduction and corresponding temperature points of the EC testbed compared with the Ref testbed and outside environment during the growing period (October 15th - 28th, 2024)

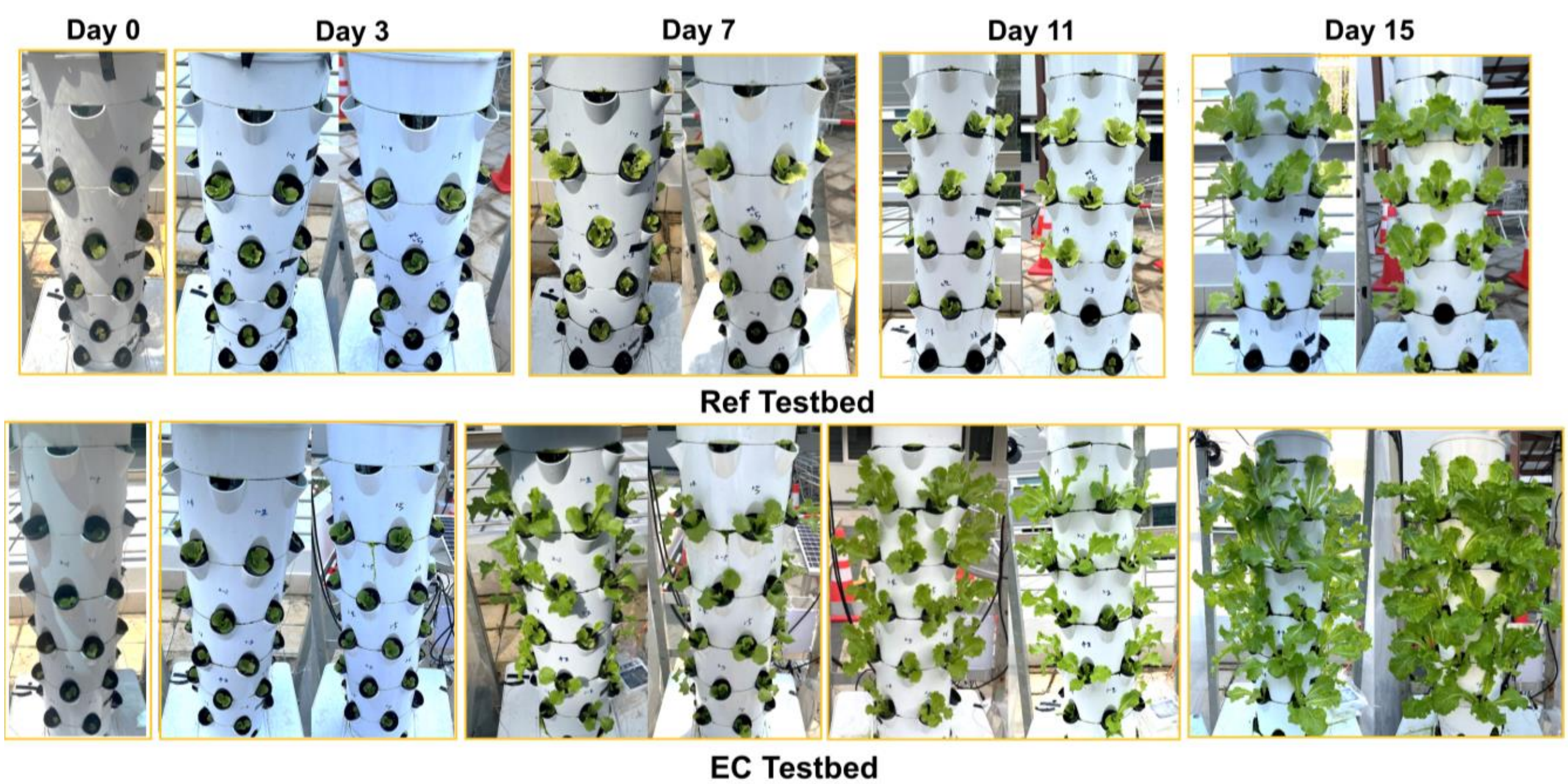

Supplementary Fig. 9. The photographs of growing variations of lettuces in the vertical tower under EC and Ref shading throughout the cultivation cycle.

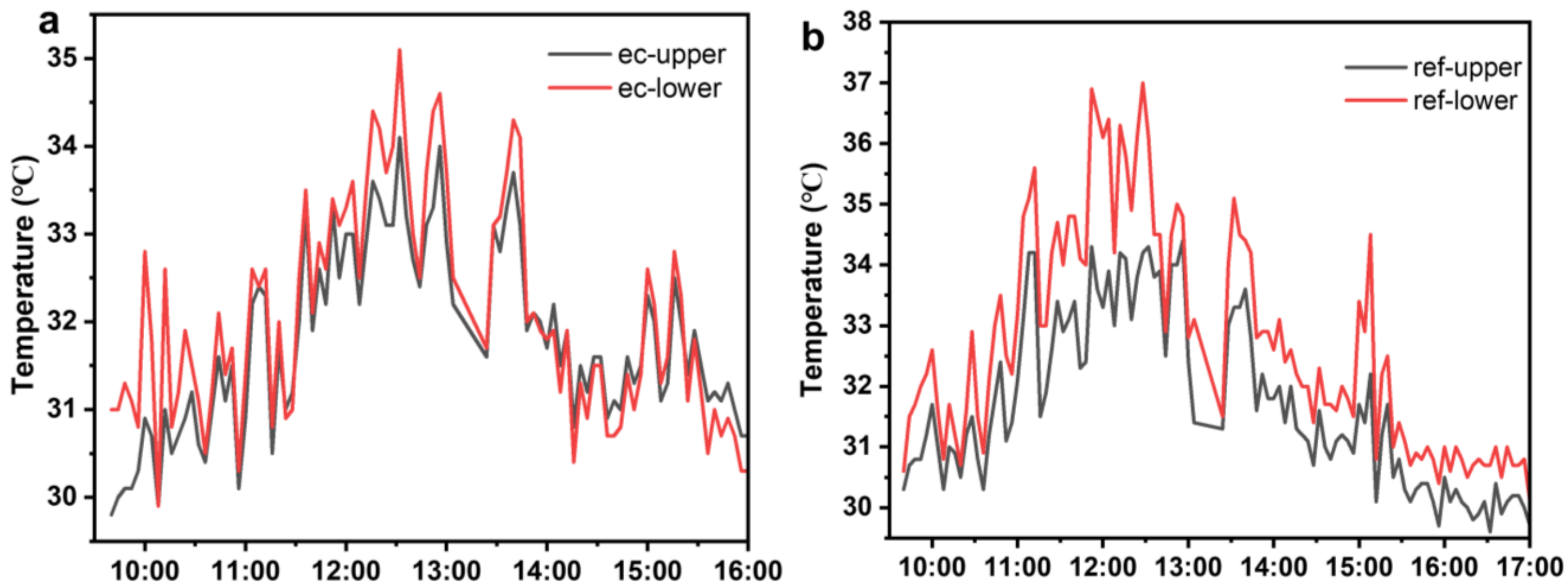

Supplementary Fig. 10. The air temperature recorded by the thermocouples on the upper/lower location of the EC/Ref towers at the same height on October 25th, 2024.

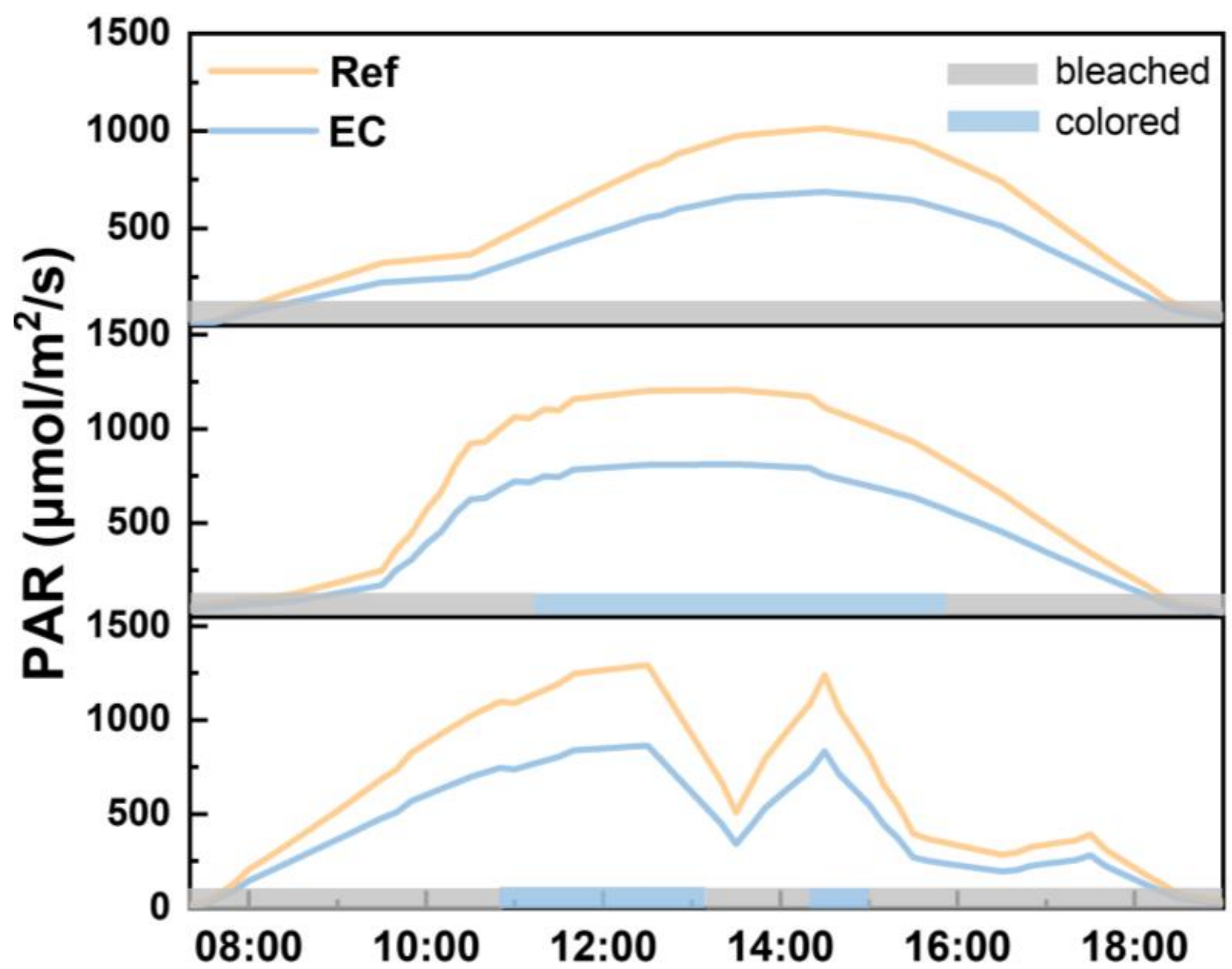

Supplementary Fig. 11. Simulated daily PAR profiles under representative sky conditions and corresponding EC switching states. Three representative days were selected from the full-year Singapore simulation to illustrate the irradiance-adaptive control performance under different weather/sky conditions: overcast (top), clear-sky (middle), and cloudy/variable (bottom). Lines show the simulated PAR (PPFD) time series for the EC roof (blue) and reference roof (orange). Shaded bars indicate the EC roof optical state (grey: bleached; blue: colored) determined by the dual-threshold switching strategy.

The overcast day exhibits low PAR and no coloration event, the clear-sky day triggers coloration during peak irradiance hours, and the cloudy/variable day shows intermittent switching in response to fluctuating irradiance.

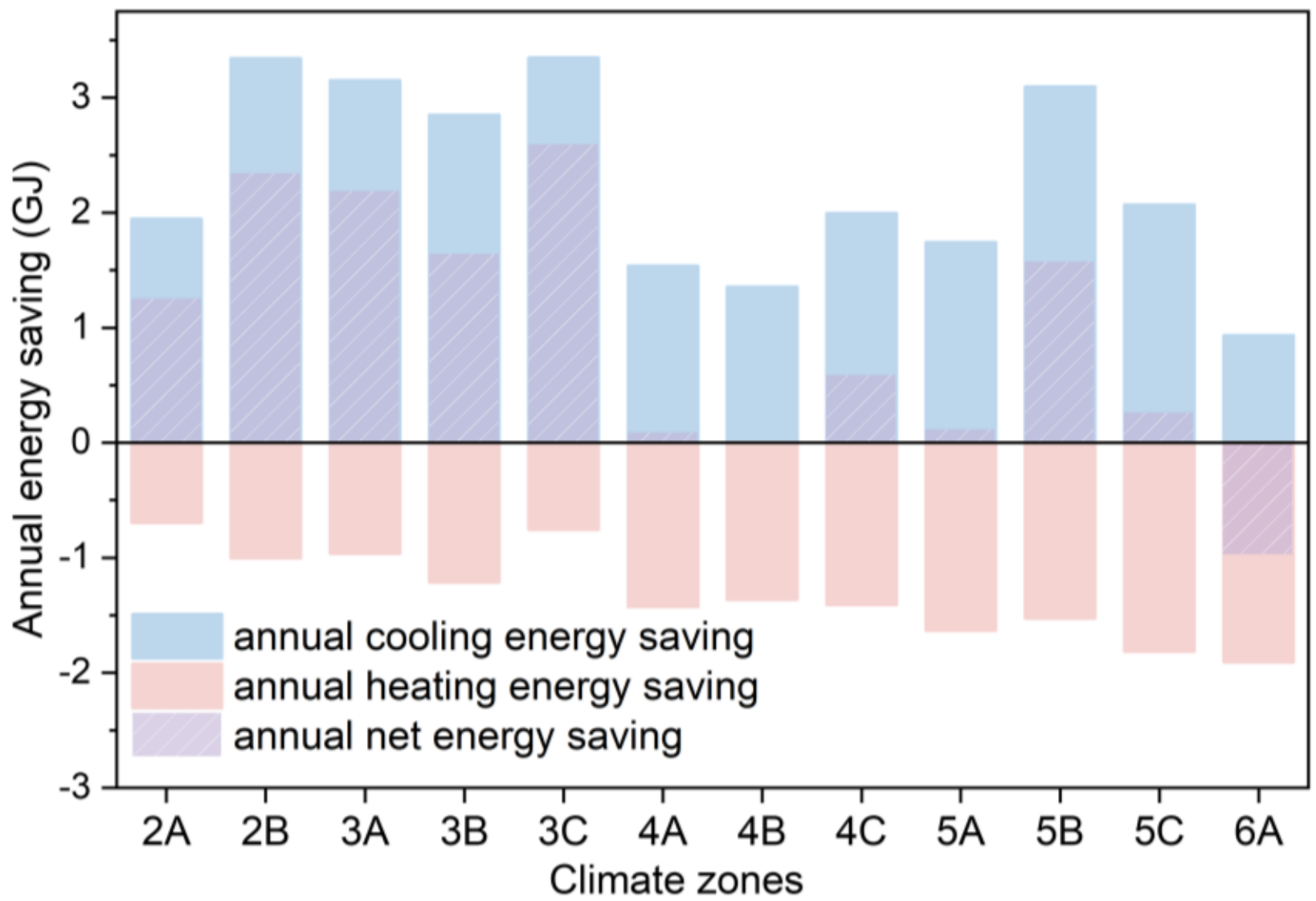

Supplementary Fig. 12. Annual cooling-energy saving, heating-energy saving (negative values indicate heating penalty), and net annual energy saving of EC shading relative to the reference roof across climate zones 2A–6A, based on year-round EnergyPlus simulations (heating setpoint: 20 °C; cooling setpoint: 24 °C).

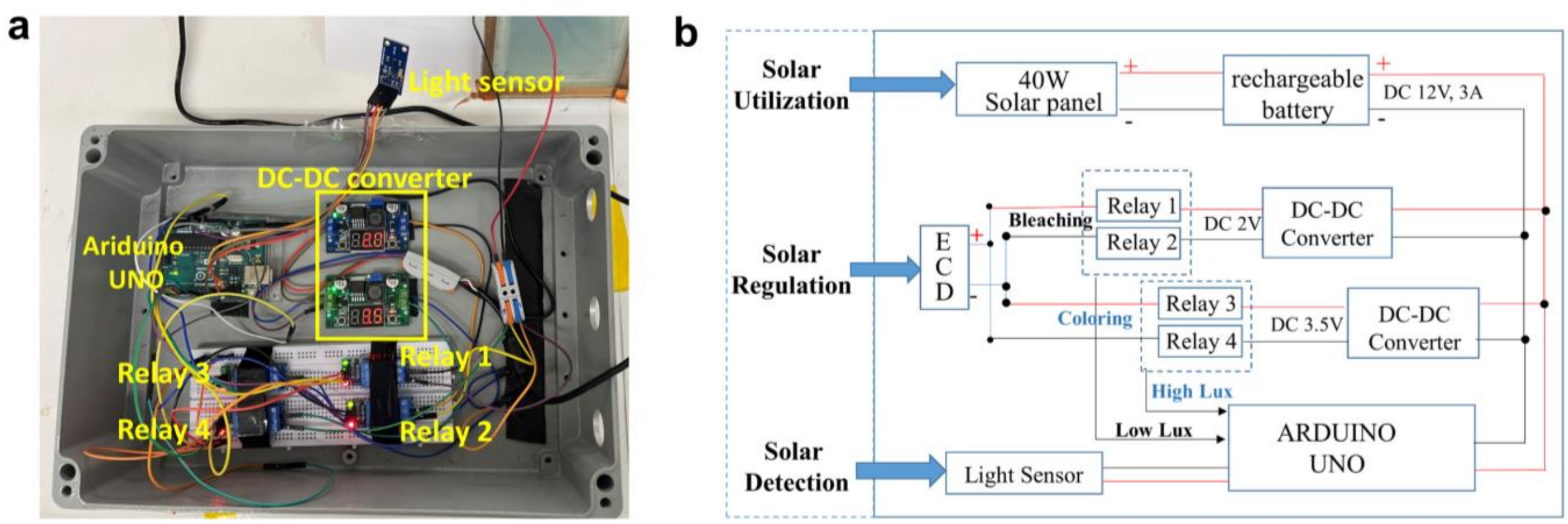

Supplementary Fig. 13. (a) Digital photo and (b) diagram of the circuit in the light sensor-controlled system.

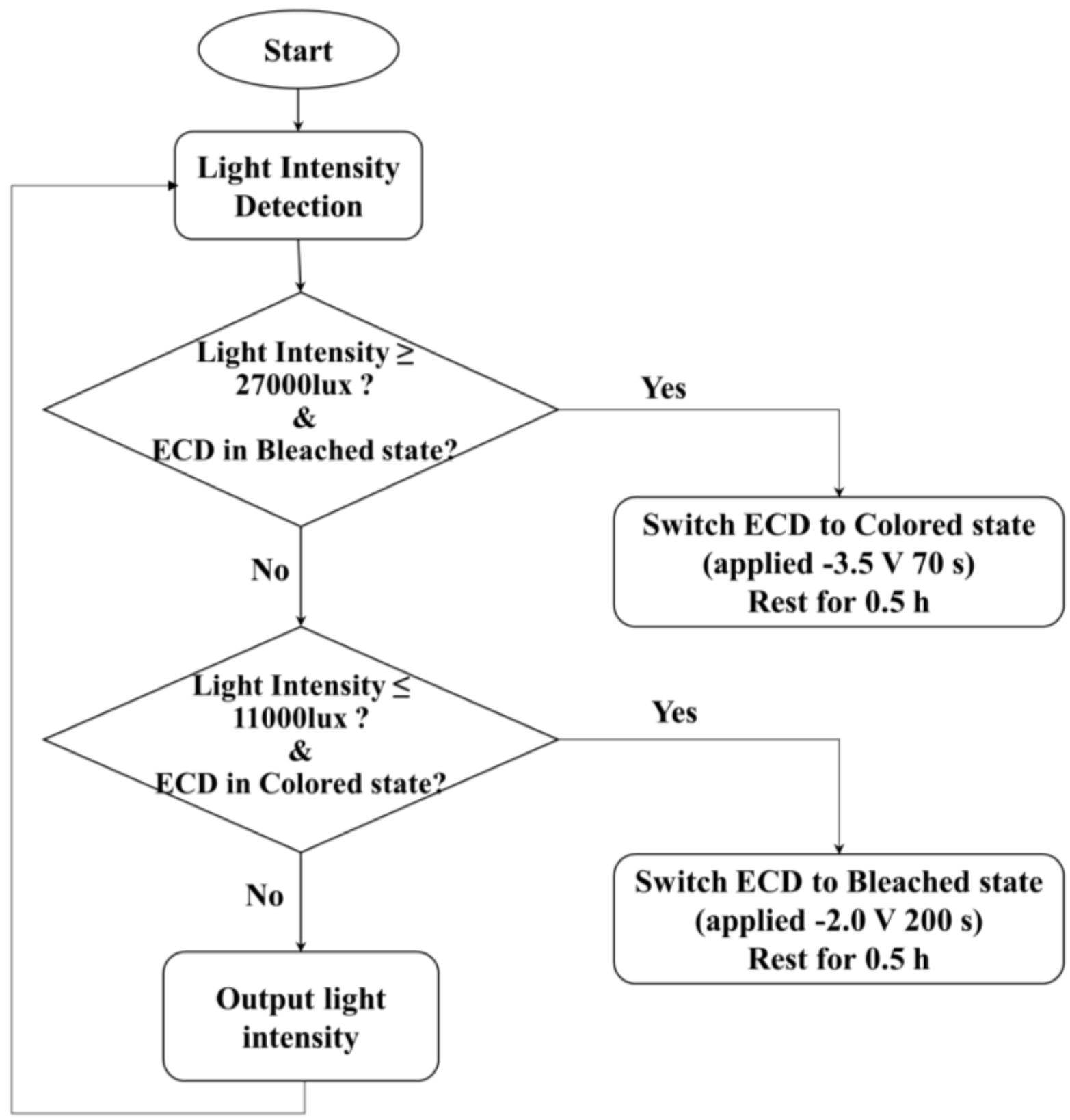

Supplementary Fig. 14. The flowchart with the setting parameters of the circuit in the light sensor-controlled system

The light sensor was set below the electrochromic shading. When the light intensity is higher than 27000 lux (equivalent to 750 μmol/m²/s under bleached electrochromic shading according to our pilot test) and the ECDs are in the bleached state, the ECD will switch to the colored state. When the light intensity is lower than 11000 lux (equivalent to 250 μmol/m²/s under colored electrochromic shading according to our pilot test) and the ECDs are in colored state, the ECD will switch to bleached state. To adapt to the fluctuating weather conditions, we set a 0.5 h rest time for efficient device operation after every switch. The light sensor was detected every second for control, whereas the PAR sensors were logged every 2 minutes; therefore, outdoor PAR was used as an independent reference and threshold to validate switching points.

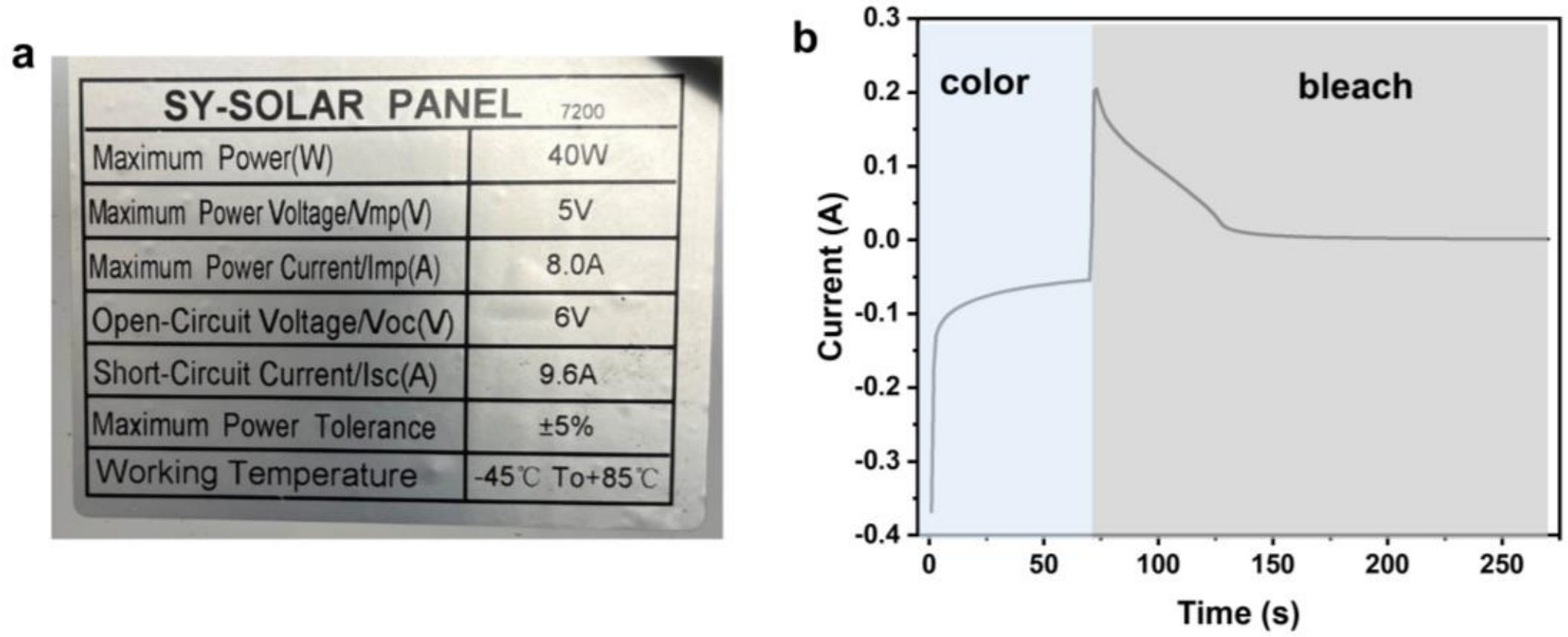

Supplementary Fig. 15. a. The solar panel (35 × 63 cm^2) parameters provided by the supplier. **b.** The current-time curves of one ECD during switch.

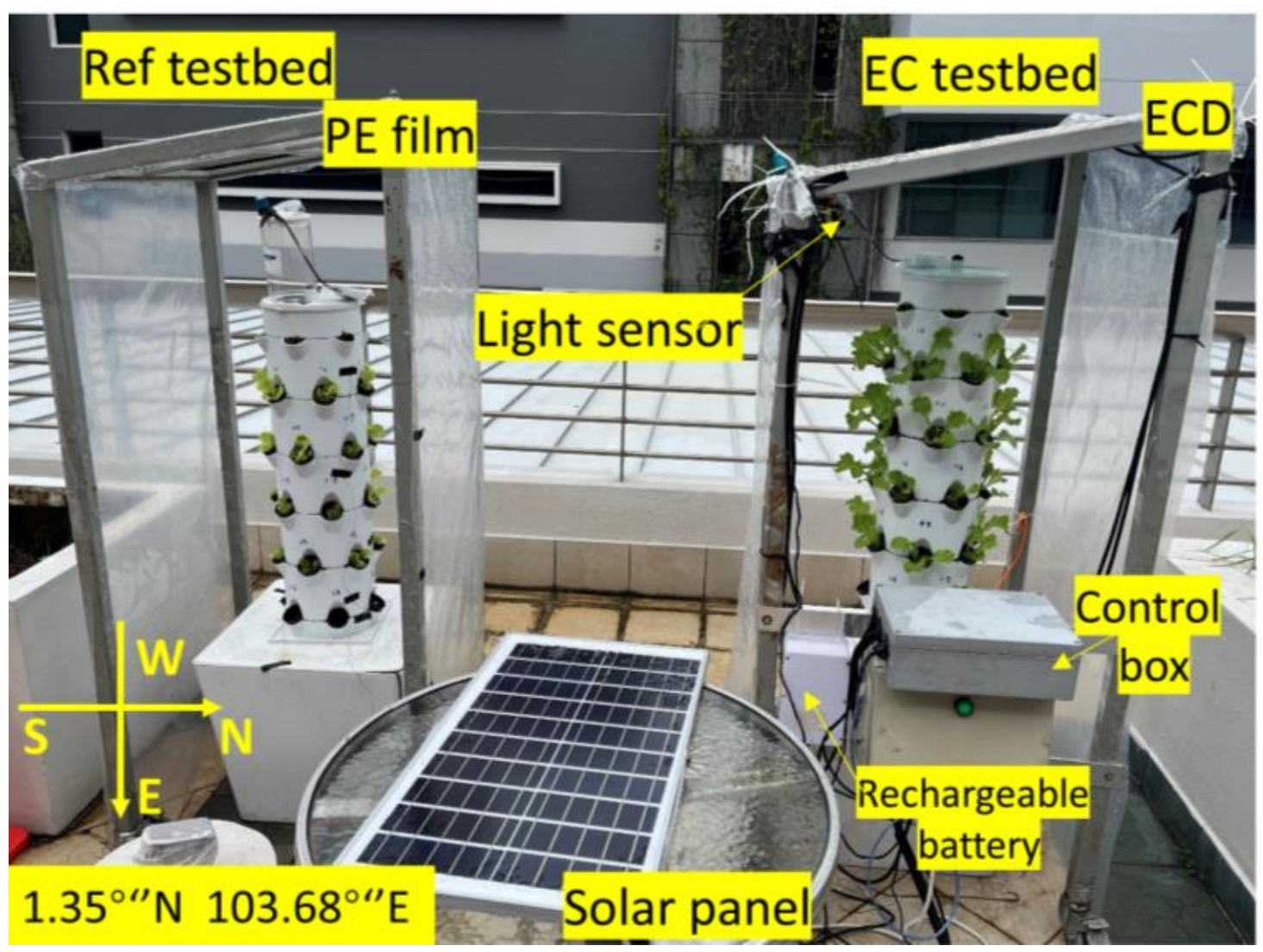

Supplementary Fig. 16. The Reference and Electrochromic testbed layout

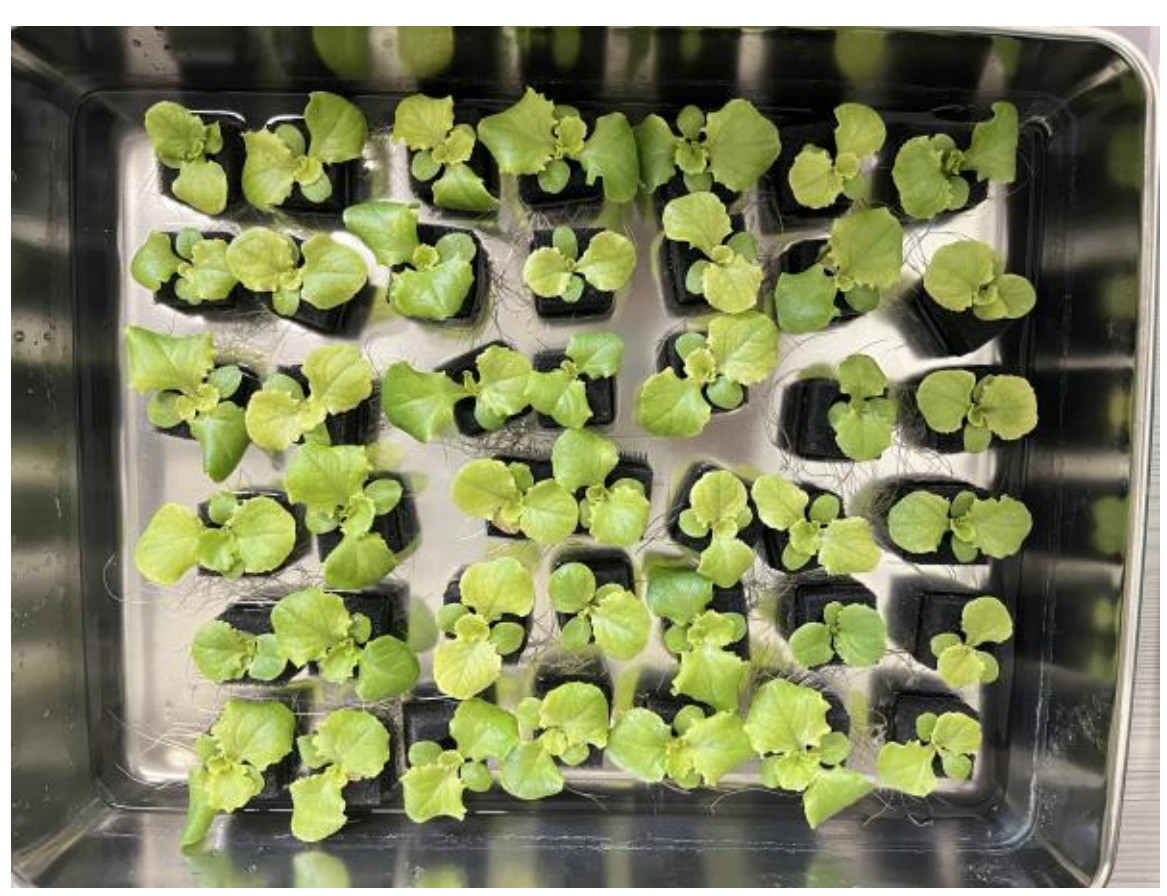

Supplementary Fig. 17. The lettuce seedlings with similar growth status before transplant

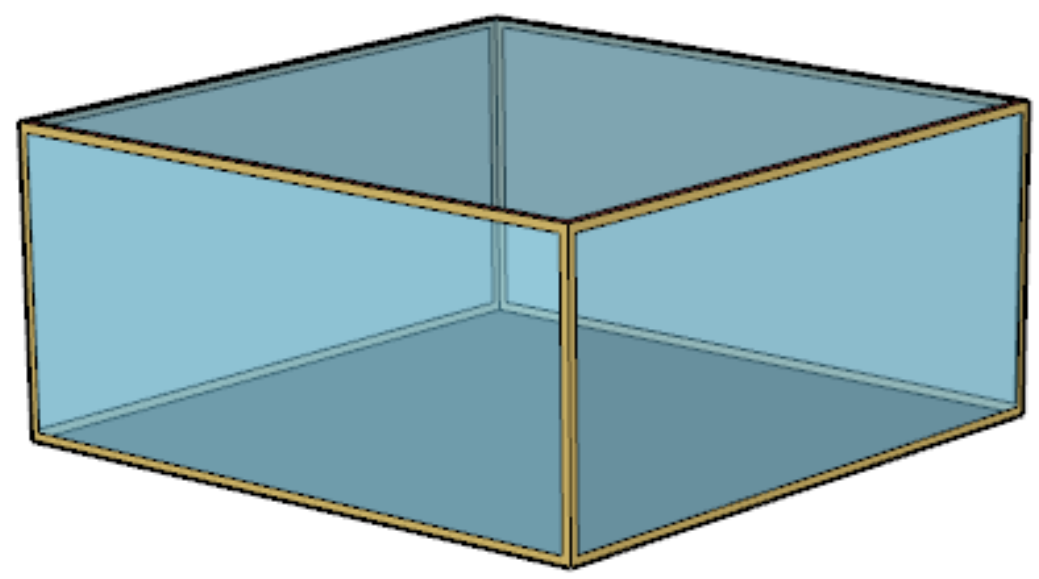

Supplementary Fig. 18. A large-scale cultivation model (3.2 m (high) × 7 m (long) × 7 m (wide)) with dynamic EC shading/commercial Ref shading

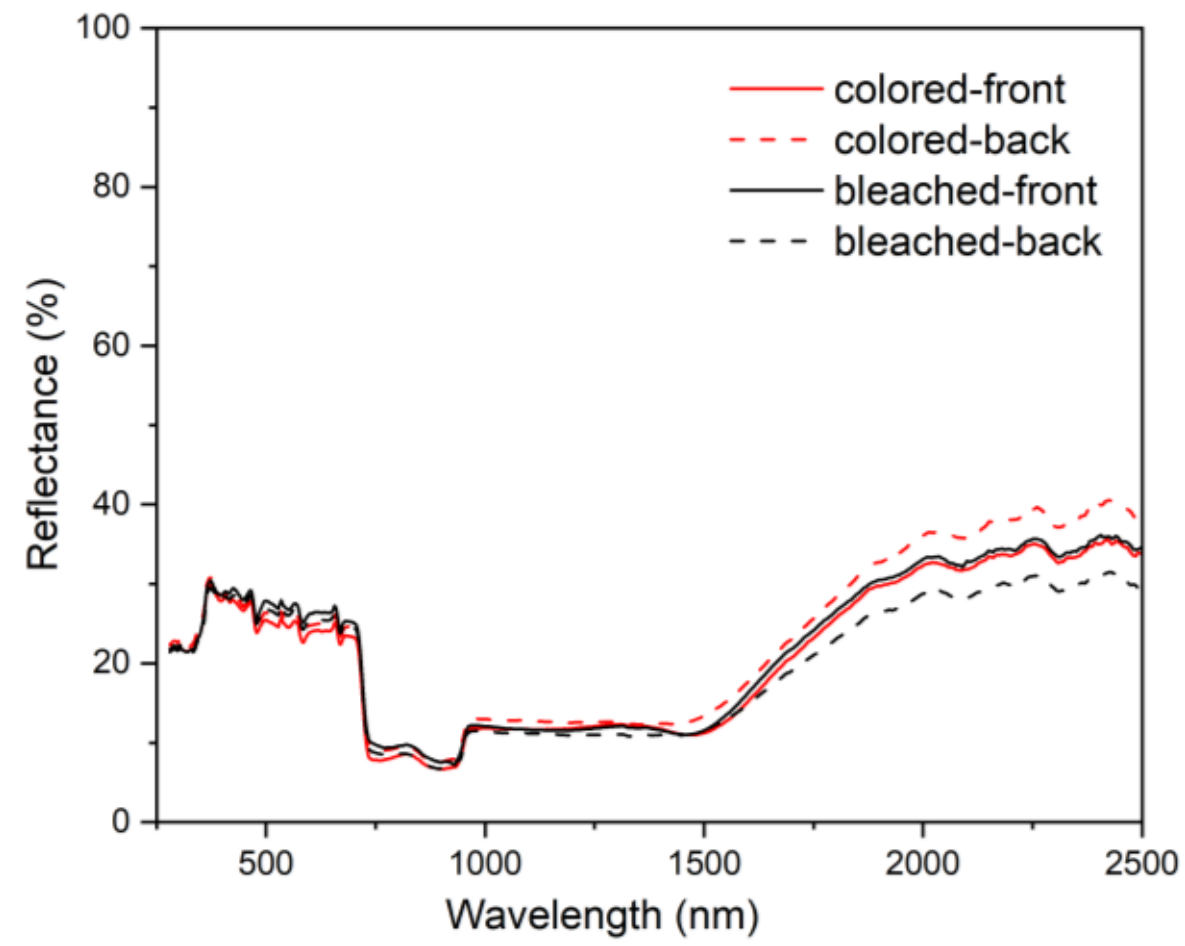

Supplementary Fig. 19. The solar reflectance spectrum of ECD with colored/bleached states

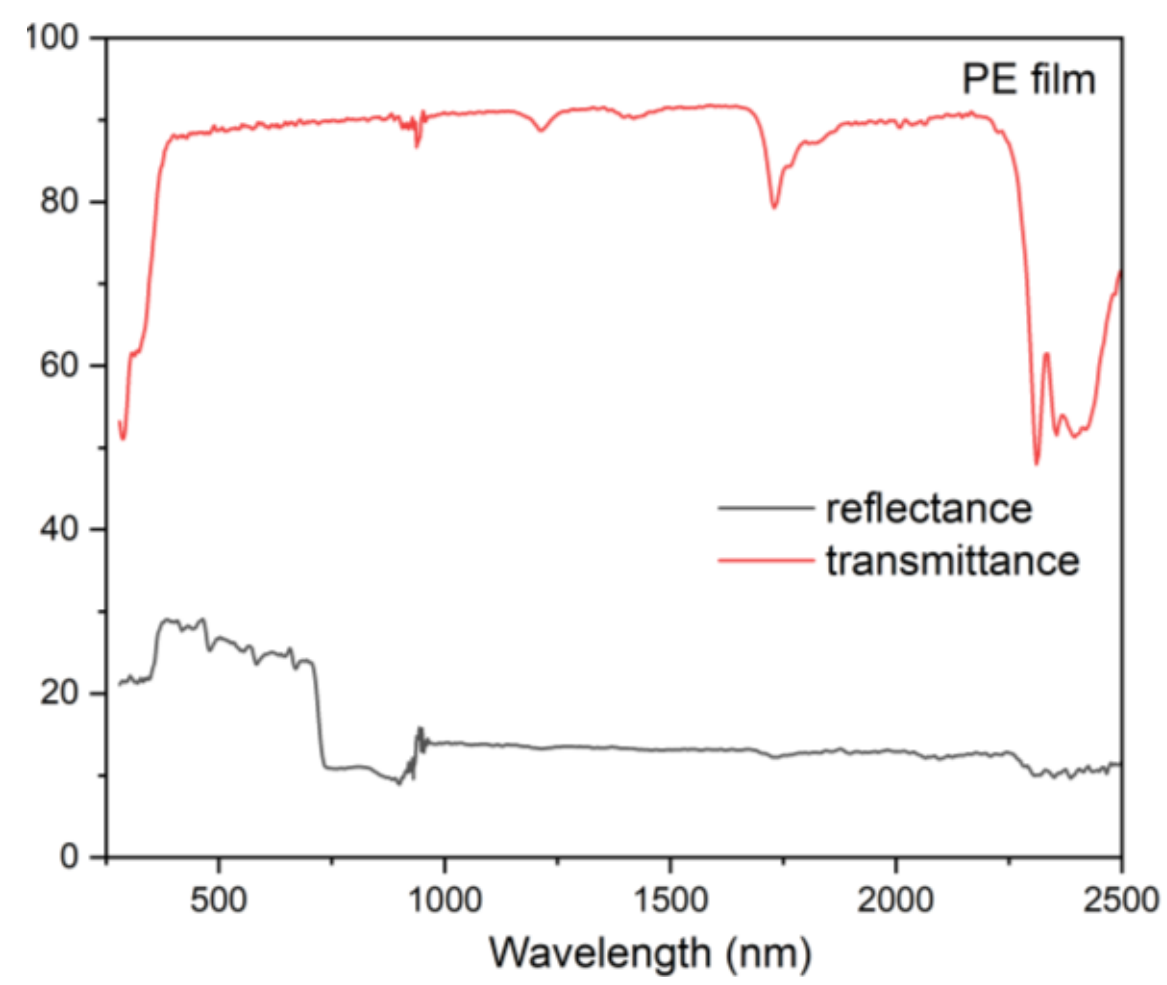

Supplementary Fig. 20. The solar reflectance and transmittance spectrum of PE film

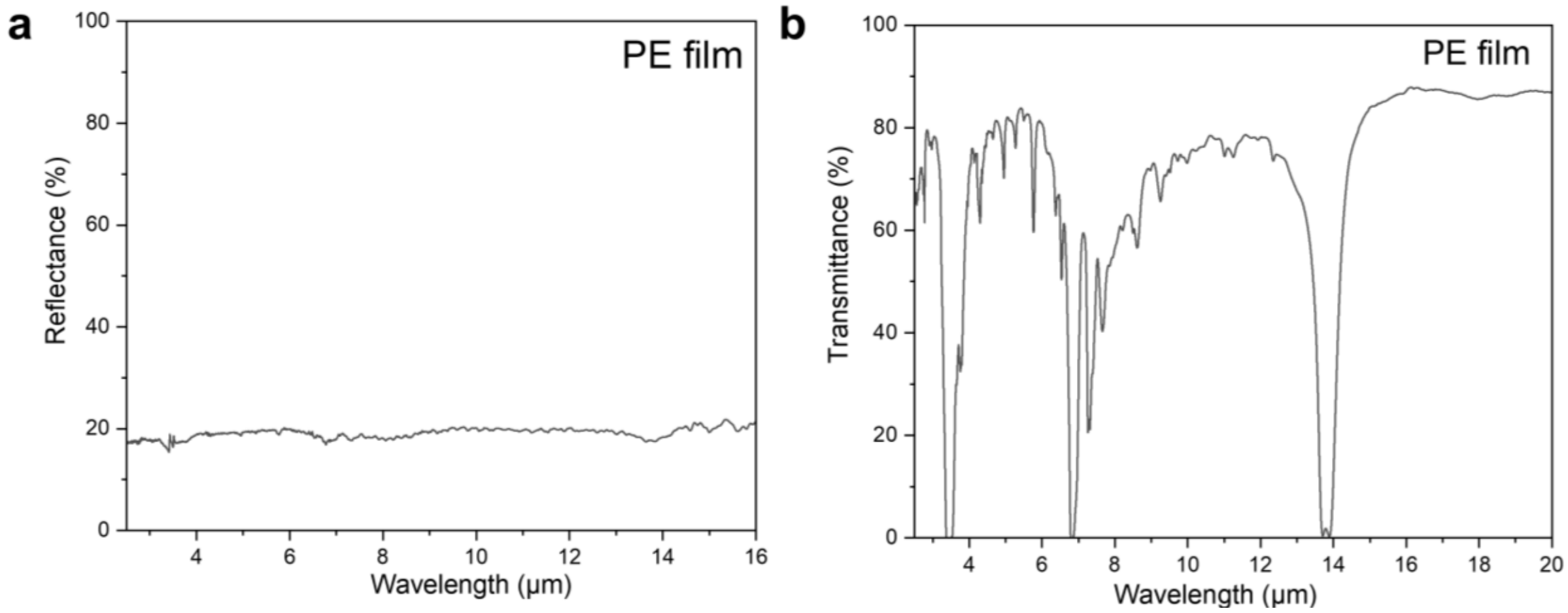

Supplementary Fig. 21. The infrared reflectance and transmittance spectrum of PE film

Supplementary Table

Supplementary Table 1. Band-integrated UV/NIR transmittance and blocking

State	T_{UV}(280-400 nm)	UV blocking	T_{VIS}(400-780 nm)	VIS blocking	T_{NIR} (780-2500 nm)	NIR blocking
Bleached	18.2%	81.8%	57.6%	42.4%	22.4%	77.6%
Colored	16.3%	83.7%	24.0%	76%	3.7%	96.3%

Band-integrated transmittances for the ECD in colored and bleached states were calculated from the measured spectral transmittance $T(\lambda)$ following the EN 410 weighted-integration framework with the following equations[6]:

$$T_{\mathrm{UV}} = \int_{280}^{400} T(\lambda)\phi_{\mathrm{SOL}}(\lambda)d\lambda \,/\, \int_{280}^{400} \phi_{\mathrm{SOL}}(\lambda)d\lambda \quad \text{(S19)}$$

$$\mathrm{T}_{\mathrm{VIS}} = \int_{400}^{780} T(\lambda)\phi_{\mathrm{VIS}}(\lambda)d\lambda \,/\, \int_{400}^{780} \phi_{\mathrm{VIS}}(\lambda)d\lambda \quad \text{(S20)}$$

$$\mathrm{T}_{\mathrm{NIR}} = \int_{780}^{2500} T(\lambda)\phi_{\mathrm{SOL}}(\lambda)d\lambda \,/\, \int_{780}^{2500} \phi_{\mathrm{SOL}}(\lambda)d\lambda \quad \text{(S21)}$$

Where $T(\lambda)$ represents the transmittance at wavelength λ, $\phi_{VIS}(\lambda)$ and $\phi_{SOL}(\lambda)$ are the standard efficiency functions for the photopic vision and the solar irradiance spectrum for an air mass of 1.5.

Supplementary Table 2. The transmitted solar energy irradiation (W/m^2) of ECD with colored/bleached states under different wavebands (UV 280 - 400 nm, VIS 400 - 780 nm, NIR 780 - 2500 nm)

	(W/m^2)	UV (280-400nm)	VIS (400-780nm)	NIR (780-2500nm)	Total
**Sun**		50	520	430	1000
**PE**	**Trans**	35.8	465	387.4	888.2
**Bleached**	**Trans**	8.6	303	97	408.6
	**Blocked**	41.4	217	333	591.4
**Colored**	**Trans**	7.6	122.4	18	148
	**Blocked**	42.4	397.6	412	852

The light energy irradiation (W/m^2) under different wavebands (UV 280 - 400 nm, VIS 400 - 780 nm, NIR 780 - 2500 nm) can be obtained from the solar irradiance spectrum for an air mass of 1.5:

$$E_{\mathrm{UV}} = \int_{280\ \mathrm{nm}}^{400\ \mathrm{nm}} T(\lambda)E_{\mathrm{e}}(\lambda)d\lambda \quad \text{(S22)}$$

$$E_{\mathrm{VIS}} = \int_{400\ nm}^{780\ nm} T(\lambda)E_{\mathrm{e}}(\lambda)d\lambda \quad \text{(S23)}$$

$$E_{\mathrm{NIR}} = \int_{780\ nm}^{2500\ nm} T(\lambda)E_{\mathrm{e}}(\lambda)d\lambda \quad \text{(S24)}$$

where $E_{\mathrm{e}}(\lambda)$ represents the energy irradiation at wavelength λ, $T(\lambda)$ represents the transmittance of the ECD with colored/bleached state at wavelength λ.

Supplementary Table 3. The PAR values (μmol/m^2/s) under different wavebands (blue 400-500 nm, green 500-600 nm and red 600-700 nm)

(μmol/m^2/s)	Blue (400-500nm)	Green (500-600nm)	Red (600-700nm)	PAR (400-700nm)	B/R ratio
**Sun**	538	698	754	1990	1:1.4
**Bleached**	290	452	461	1200	1:1.6
**Colored**	200	196	114	510	1:0.6
**PE**	476	618	688	1772	1:1.45

The number of photons per area in different wavebands (blue 400-500 nm, green 500-600 nm and red 600-700 nm), referred to as the Photosynthetic Photon Flux Density (PPFD), is given in μmol/m²/s:

$$\mathrm{PAR} = \int_{400\,nm}^{700\,nm} T(\lambda)E_{\mathrm{e}}(\lambda)K(\lambda)d\lambda \quad \text{(S25)}$$

$$\mathrm{PAR(Blue)} = \int_{400\,nm}^{500\,nm} T(\lambda)E_{\mathrm{e}}(\lambda)K(\lambda)d\lambda \quad \text{(S26)}$$

$$\mathrm{PAR(Green)} = \int_{500\,nm}^{600\,nm} T(\lambda)E_{\mathrm{e}}(\lambda)K(\lambda)d\lambda \quad \text{(S27)}$$

$$\mathrm{PAR(Red)} = \int_{600\,nm}^{700\,nm} T(\lambda)E_{\mathrm{e}}(\lambda)K(\lambda)d\lambda \quad \text{(S28)}$$

$$K(\lambda) = \frac{1}{N_{\mathrm{A}}\frac{hc}{\lambda}} = \frac{\lambda}{hcN_{\mathrm{A}}} \quad \text{(S29)}$$

$$E_{\mathrm{PAR}} = \int_{400\,nm}^{700\,nm} E_{\mathrm{e}}(\lambda)d\lambda \quad \text{(S30)}$$

Where λ represents the wavelength of the photon, T(λ) represents the transmittance of the ECD with colored/bleached state at wavelength λ, $E_e(\lambda)$ represents the energy irradiation at wavelength λ, *h* is the Planck's constant, *c* is the speed of light, N_A is the Avogadro number.

Supplementary Table 4. Switching condition parameters of the electrochromic shading system (under-ECD illuminance/PAR/energy irradiance, and corresponding outdoor levels)

Trigger point	Parameter	Outside	Under Bleached EC	Under Colored EC
Bleach-color	Illuminance (lux)	-	27000	-
	PPFD (μmol/m²/s)	1240	750	-
	Global solar energy irradiation (W/m^2)	530	-	-
Color-bleach	Illuminance (lux)	-	-	11000
	PPFD (μmol/m²/s)	890	-	250
	Global solar energy irradiation (W/m^2)	380	-	-

To better set up the switching condition parameters of the electrochromic shading, we mapped the real-time control signal (under-shade illuminance, lux) to the crop-relevant transmitted PAR thresholds and the corresponding outdoor reference levels. In the outdoor pilot test, switching was triggered using the under-shade illuminance thresholds (27000 lx for coloring and 11000 lx for bleaching), because the light sensor provides high-frequency lux readings for real-time control. The transmitted PAR thresholds were selected to maintain the PAR value within the optimum range of 250 to 750 μmol/m²/s for leafy greens, such as lettuce,[7,8] the system is operated according to the following logic: when the PAR value of the testbed under bleached ECD exceeds 750 μmol/m²/s, the ECD will be triggered to the colored state. In this situation, the PAR value of the outside (sunlight) is recorded as 1240 μmol/m²/s. When the PAR value of the testbed under colored ECD is lower than 250 μmol/m²/s, the ECD will be triggered to the bleached state. In this situation, the PAR value of outside (sunlight) is recorded as 890 μmol/m²/s. The recorded corresponding outdoor PAR values function as independent references for validating switching events.

For EnergyPlus simulation, equivalent switching logic was implemented using the outdoor irradiance variable required by the EMS. Outdoor PAR (PPFD) was converted to outdoor global solar irradiance using literature-based approximations: PPFD was first converted to PAR radiant flux using a typical factor of ~4.6 μmol/J,[9] and PAR was assumed to represent ~50% of the total broadband solar energy under representative solar spectra.[10] This yields the irradiance thresholds of 530 W/m^2 (for coloration) and 380 W/m^2 (for bleaching). We note that this conversion is approximate and may vary with spectral distribution; however, it provides a consistent mapping for implementing the irradiance-based EMS switching logic.

Supplementary Table 5. Optical and thermal properties of the roof shading/wall materials used in the EneryPlus model

Property	unit	EC roof (colored)	EC roof (bleached)	Reference PE film/ Side wall
Optical data type	-	spectral average	spectral average	spectral average
Thickness	m	0.01365	0.01365	0.000135
Solar transmittance	-	0.150	0.411	0.888
Front side solar reflectance	-	0.187	0.200	0.191
Back side solar reflectance	-	0.198	0.190	0.191
Visible transmittance	-	0.240	0.576	0.891
Front side visible reflectance	-	0.229	0.248	0.240
Back side visible reflectance	-	0.238	0.240	0.240
Infrared transmittance	-	0	0	0.692
Front side infrared emissivity	-	0.89	0.89	0.118
Back side infrared emissivity	-	0.89	0.89	0.118
Conductivity	W/(m·K)	0.83	0.83	0.33
Dirt correction factor	-	1	1	1
Solar diffusing	-	no	no	no

The integral solar transmittance (T_{SOL} = 280-2500 nm) and visible transmittance (T_{VIS} = 400-780 nm) of the ECD under colored/bleached state and PE film were obtained based on the corresponding transmittance spectrum (Fig. 1c) with the following equations:

$$T_{\mathrm{SOL}} = \int \phi_{\mathrm{SOL}}(\lambda) T(\lambda) d\lambda / \int \phi_{\mathrm{SOL}}(\lambda) d\lambda \quad \text{(S31)}$$

$$T_{\mathrm{VIS}} = \int \phi_{\mathrm{VIS}}(\lambda) T(\lambda) d\lambda / \int \phi_{\mathrm{VIS}}(\lambda) d\lambda \quad \text{(S32)}$$

where $T(\lambda)$ represents the transmittance at wavelength λ, and ϕ_{VIS} and ϕ_{SOL} are the standard

efficiency function for photopic vision and the solar irradiance spectrum for an air mass of 1.5.

The integral solar reflectance (R_{SOL} = 280-2500 nm) and visible reflectance (R_{VIS} = 400-780 nm) of the ECD with back/front side under colored/bleached state and PE film were obtained based on the corresponding reflectance spectrum (Supplementary Figs. 19 and 20) with the following equations:

$$R_{\mathrm{SOL}} = \int \phi_{\mathrm{SOL}}(\lambda) R(\lambda) d\lambda / \int \phi_{\mathrm{SOL}}(\lambda) d\lambda \tag{S33}$$

$$R_{\mathrm{VIS}} = \int \phi_{\mathrm{VIS}}(\lambda) R(\lambda) d\lambda / \int \phi_{\mathrm{VIS}}(\lambda) d\lambda \tag{S34}$$

where $R(\lambda)$ represents the reflectance at wavelength λ, and ϕ_{VIS} and ϕ_{SOL} are the standard efficiency function for photopic vision and the solar irradiance spectrum for an air mass of 1.5.

The infrared transmittance (T_{IR} = 2.5-20 μm) and infrared emissivity (ε_{IR} = 2.5-20 μm) of the ECD with back/front side mainly come from the quartz glass substrate, the glass substrate has no transmittance in this region[11], and the infrared emissivity was referred to the reported literature.[12] And the infrared transmittance (T_{IR} = 2.5-20 μm) and infrared reflectance (R_{IR} = 2.5-20 μm) of commercial PE film were obtained based on the corresponding spectrum in the infrared region (Fig. S18) with the following equations:

$$T_{\mathrm{IR}} = \int L_{\mathrm{IR}}(\lambda) T(\lambda) d\lambda / \int L_{\mathrm{IR}}(\lambda) d\lambda \tag{S35}$$

$$R_{\mathrm{IR}} = \int L_{\mathrm{IR}}(\lambda) R(\lambda) d\lambda / \int L_{\mathrm{IR}}(\lambda) d\lambda \tag{S36}$$

where $T(\lambda)$ represents the transmittance at wavelength λ and $R(\lambda)$ represents the emissivity at wavelength λ, and L_{IR} are the spectral radiance based on room-temperature black-body radiation spectrum. Then the infrared emissivity ε_{IR} was determined by 1-T_{IR}-R_{IR}.

Supplementary Table 6. Geometry, EC control logic, and simulation settings of the cultivation model

Parameter	Unit	Value	Description
Length	m	7	Zone geometry
Width	m	7	Zone geometry
Height	m	3.2	Zone geometry
Floor area, A_f	m^2	49	Floor area used in the analysis
Volume	m^3	156.8	Zone volume
Roof area, A_{roof}	m^2	49	Roof area in Eq. (S4)
Single side-wall area, $A_{wall,j}$	m^2	22.4	Area of each wall in Eq. (S4)
Number of side walls	–	4	Greenhouse enclosure
Weather file	–	Singapore EPW	Annual simulation
Cooling setpoint	°C	24	ZoneHVAC:IdealLoadsAirSystem
EC coloring threshold, E_{col}	W/m^2	530	EC Roof shading switches to colored state above this level, used in Eq. (S1)
EC bleaching threshold, E_{ble}	W/m^2	380	EC Roof shading switches to bleached state below this level, Eq. (S1)
State-retention range	W/m^2	380–530	Previous state retained
Simulation period	–	Full year	Annual run
Simulation timestep	min	10	Used in Eqs. (S13)–(S14)

Supplementary Table 7. Illuminance sampling and effective radiative-input parameters

Parameter	Unit	Value	Description
Lowest sampling height, z_1	m	0.6	First illuminance point
Vertical interval, Δz	m	0.16	Distance between adjacent sampling points
Highest sampling height	m	3.0	Effective top of the cultivation zone
Number of sampling points, N	–	16	Used in Eqs. (S2) and (S3)
Thermal-radiation fraction, f_{th}	–	10%	Fraction of the total effective radiative heat input represented as supplementary radiative term from reported studies[13]
Crop representation	–	Internal cultivation zone, no explicit plant geometry	Used for light-regulation and cooling-energy analysis
Additional mechanical ventilation	–	No	No extra mechanical ventilation schedule imposed

Supplementary Table 8. Comparison between EC shading with other light-thermal regulation technologies, including radiative cooling, photoselective cover, smart glass cover, and NIR-reflective cover.

Cover	Materials	Plant species	Plant growth	Light regulation	Thermal regulation	Dynamic function	Ref
Smart glass film ULR-80	Glass-based	eggplant	28% mean fruit number<br>-32% fruit weight<br>-17% biomass<br>+8% total sugars<br>+29% sucrose	Block 85% UV,19% PAR, 88% NIR	0.5-0.9℃	×	14
Radiative cooling film	Emitter-PEN-PMMA-PET stacks	Spinach, lettuce, Chinese little greens	+78.5%/37.5% (spinach)<br>+245%/179% (lettuce)<br>+100%-300% (little greens)<br>biomass than PE/bare	Block PAR (48% 400-480nm, 15% 600-700nm, 100% 480-600nm) and 100% NIR; 91% MIR emissivity	2.3-5.0℃	×	15
Radiative cooling film	PDMS-SiO_2-TiO_2-SiO_2-Al_2O_3-Ag-Al_2O_3-TiO_2-SiO_2-TiO_2-PAM	Lettuce, Soybean, Gardenia	20%-370% biomass yield increase	Block PAR (29% 400-500nm, 23% 600-700nm);70% NIR; 92% MIR emissivity	1.9-4.6℃	×	16
Radiative cooling film	PVDF-Ag-SiO_2-Ag-SiO_2-PET	Cabbage, Pakchoi, Cherry radish	+23.4%/17.3% leaf width<br>76.2%/120.1% nutrient organs<br>-62.7% PRO contents	Block 85% UV,31% PAR, 79% NIR; 80-90% MIR emissivity	2-6℃	×	17
NIR filter film	/	Tomato	10%-12% production increase	Block 10% PAR, 100% NIR	1-2℃	×	18
Photoselective shading	Shade net	/	/	Block 40%-50% PAR and regulate R/FR ratio	×	×	19
EC shading	FTO glass-WO_3-Electrolyte-FTO glass	Lettuce	+235% biomass<br>+31% leaf number<br>+60%-64% pigment<br>+61.9% total sugar<br>+23.7% vitamin C<br>-27.9% PRO	**Colored**: Block 84.8% UV,78% NIR, **39.5% PAR; B/R=1:0.6**<br>**Bleached**: Block 82.8% UV, 96% NIR, **74.2% PAR; B/R=1:1.6**	1.2-3.8℃	√	This work

Supplementary Table 9. The parameters of the testbed

Location		Testbed Dimension		Tower Dimension	
City	Singapore	Height (longer side)	1.6 m	Height	1.4 m
Longitude	103.68°E	Height (shorter side)	1.55 m	Width	0.24 m
Latitude	1.35°N	Roof area	0.7*0.7 m^2	Length	0.24 m
Altitude	54m	Roof Angle	4°	Vegetable planting height	0.4~1.4 m

Nomenclature/Symbols

Symbol	Definition	Unit
A	floor area	m^2
A_{roof}	Roof area	m^2
$A_{wall,j}$	Area of wall (j)	m^2
A_s	Area of interior surface (s)	m^2
C_s	Thermal capacitance of interior surface (s)	J/K
C_z	Thermal capacitance of zone air	J/K
C_p	Specific heat capacity of air	J/(kg·K)
$E_{roof}(t)$	Incident solar radiation on the roof surface at time (t)	W/m^2
$E_{wall,j}(t)$	Incident solar radiation on wall (j) at time (t)	W/m^2
E_{col}	EC roof coloring threshold	W/m^2
E_{ble}	EC roof bleaching threshold	W/m^2
$E_{cool,n}$	Cooling energy at timestep (n)	J
$E_{cool,m}$	Monthly cooling-energy	J
$E_{cool,year}$	Annual cooling-energy	J
$E_{cool,P}$	cooling-energy for selected period	J
f_{th}	Fraction of the total effective radiative heat input represented as thermal radiation	–
$h_{c,s}(t)$	Inside convective heat-transfer coefficient of surface (s)	$W/(m^2{\cdot}K)$
$I_i(t)$	Total illuminance at sampling point (i) and time (t)	lux
$I_{dir,i}(t)$	Direct transmitted daylight illuminance at point (i)	lux
$I_{dif,i}(t)$	Diffuse transmitted daylight illuminance at point (i)	lux
$I_{ref,i}(t)$	Internally reflected visible-radiation illuminance at point (i)	lux
$\bar{I}(t)$	Mean illuminance within the cultivation zone at time (t)	lux
N	Number of illuminance sampling points	–
N_{year}	Total number of 10-minute timesteps in one year	–
$q_{conv,s}(t)$	Convective heat exchange between interior surface (s) and zone air	W
$q_{cond,s}(t)$	Conductive heat transfer through interior surface (s)	W

$q_{\mathrm{cool}}(t)$	Instantaneous cooling rate	W
$q_{\mathrm{LW,s}}(t)$	Net longwave radiative exchange at interior surface (s)	W
$q_{\mathrm{rad}}(t)$	Transmitted shortwave solar radiation entering the greenhouse	W
$q_{\mathrm{sol,s}}(t)$	Absorbed radiative heat gain at interior surface (s)	W
$q_{\mathrm{sol,tot}}(t)$	Total absorbed indoor radiative gain	W
$q_{\mathrm{th}}(t)$	Thermal-radiation component of the effective radiative input	W
$q_{\mathrm{use}}(t)$	Total effective radiative heat input across the cultivation region	W
$Q_{\mathrm{int,sens}}(t)$	Net sensible internal gain in the zone air balance	W
$T_{\mathrm{out}}(t)$	Outdoor air temperature	°C or K
$T_{\mathrm{sup}}(t)$	Supply-air temperature	°C or K
$T_s(t)$	Interior surface temperature	°C or K
$T_{\mathrm{z}}(t)$	Zone air temperature	°C or K
t	Simulation time	–
ΔE_m	Monthly cooling-energy saving of the EC roof	J
ΔE_{year}	Annual cooling-energy saving of the EC roof	J
ΔE_P	cooling-energy saving of the EC roof for selected period	J
η_{m}	Monthly cooling-energy saving rate	%
$m_{\mathrm{inf}}(t)$	Infiltration / outdoor-air exchange rate	kg/s
$m_{\mathrm{sys}}(t)$	HVAC supply-air mass flow rate	kg/s
$T_{\mathrm{EC}}(t)$	EC roof transmittance at time (t)	–
$T_{\mathrm{roof}}(t)$	Roof shading (EC/Ref) solar transmittance at time (t)	–
T_{wall}	Side-wall solar transmittance	–
T_{col}	EC roof transmittance in the colored state	–
T_{ble}	EC roof transmittance in the bleached state	–
$\Psi_s(t)$	Effective surface-specific radiative-distribution coefficient	–
z_i	Height of sampling point (i) above the floor	m
z_1	Lowest sampling height	m
Δz	Vertical interval between adjacent sampling points	m

Supplementary References

1. Singh, H., Dunn, B. L., Payton, M. & Brandenberger, L. Selection of Fertilizer and Cultivar of Sweet Pepper and Eggplant for Hydroponic Production. *Agronomy* **9**, 433 (2019).
2. Ronoh, E. K. & Rath, T. Modelling of longwave radiation exchange at greenhouse surfaces under all-sky conditions. *Agricultural Engineering International: CIGR Journal* **17**, 23–35 (2015).
3. Papadakis, G., Frangoudakis, A. & Kyritsis, S. Theoretical and experimental investigation of thermal radiation transfer in polyethylene covered greenhouses. *Journal of Agricultural Engineering Research* **44**, 97–111 (1989).
4. Wang, C., Zou, H., Huang, D., Yang, R. & Wang, R. Enhancing food production in hot climates through radiative cooling mulch: A nexus approach. *Nexus* **1**, (2024).
5. Hassanien, R. H. E. & Ming, L. Influences of greenhouse-integrated semi-transparent photovoltaics on microclimate and lettuce growth. *Int J Agric & Biol Eng* **10**, 11–22 (2017).
6. de Normalisation, C. E. Glass in Building–Determination of Luminous and Solar Characteristics of Glazing. *Brussels (Belgium): CEN, Publicatio n No. EN* **410**, 64 (2011).
7. Ahmed, H. A., Yu-Xin, T. & Qi-Chang, Y. Optimal control of environmental conditions affecting lettuce plant growth in a controlled environment with artificial lighting: A review. *South African Journal of Botany* **130**, 75–89 (2020).
8. Fu, W., Li, P., Wu, Y. & Tang, J. Effects of different light intensities on anti-oxidative enzyme activity, quality and biomass in lettuce. *Horticultural Science* **39**, 129–134 (2012).
9. Arst, H., Erm, A., Kangro, K., Nõges, T. & Nõges, P. Comparison of spectral and broad-band models for computing photosynthetically absorbed radiation in turbid waters. *Boreal environment research* **11**, 55 (2006).
10. Amthor, J. S. From sunlight to phytomass: on the potential efficiency of converting solar radiation to phyto-energy. *New Phytologist* **188**, 939–959 (2010).
11. Biswas, P. K. *et al.* Effects of tin on IR reflectivity, thermal emissivity, Hall mobility and plasma wavelength of sol–gel indium tin oxide films on glass. *Materials Letters* **57**, 2326–2332 (2003).
12. Shao, Z. *et al.* Tri-band electrochromic smart window for energy savings in buildings. *Nature Sustainability* **7**, 796–803 (2024).

13. Al-Helal, I. M. & Abdel-Ghany, A. M. Energy partition and conversion of solar and thermal radiation into sensible and latent heat in a greenhouse under arid conditions. *Energy and Buildings* **43**, 1740–1747 (2011).
14. Chavan, S. G. *et al.* Light-limited photosynthesis under energy-saving film decreases eggplant yield. *Food and Energy Security* **9**, e245 (2020).
15. Jiang, S. *et al.* A Low-Cost and Large-Scale Producible Polymer Multilayer Radiative Cooling Film for Reducing Plant Heat Stress. *ACS Photonics* **12**, 528–536 (2025).
16. Li, J. *et al.* A photosynthetically active radiative cooling film. *Nature Sustainability* **7**, 786–795 (2024).
17. Zou, H. *et al.* Eliminating greenhouse heat stress with transparent radiative cooling film. *Cell Reports Physical Science* **4**, (2023).
18. Hemming, S., Kempkes, F., van der Braak, N., Dueck, T. & Marissen, N. Filtering natural light at the greenhouse covering-better greenhouse climate and higher production by filtering out NIR? in 411–416 (2005).
19. Arthurs, S. P., Stamps, R. H. & Giglia, F. F. Environmental Modification Inside Photoselective Shadehouses. *HortScience horts* **48**, 975–979 (2013).
