# Supplementary material for: Scalable irradiance-adaptive electrochromic shading for photothermal regulation
Source: Nat Commun. 2026 Jul 28;17:9126. doi: 10.1038/s41467-026-76115-0 (PMC13518875; doi:10.1038/s41467-026-76115-0)
Supplement: Supplementary file 2 [file 41467_2026_76115_MOESM2_ESM.pdf]

nature portfolio

Peer Review File

Scalable irradiance-adaptive electrochromic shading for photothermal regulation

Corresponding Author: Professor Pooi See Lee

This file contains all reviewer reports in order by version, followed by all author rebuttals in order by version.

Version 0:

Reviewer comments:

Reviewer #1

(Remarks to the Author)
The manuscript deals with the integration of Electrochromic devices in greenhouses, investigating the effect on lettuce growth. The topic is indeed interesting, but the manuscript should undergo major revisions before being considered suitable for publication.

In the introduction section, the authors should improve the list of bibliographic references. The bibliographic section does not clarify the positioning of this work with respect to the state of the art. The authors should report a wide comparison with previous works dealing with the same topic. The authors should clarify what constitutes the degree of innovation of the proposed work.

With reference to the architecture proposed for the electrochromic device, it would be useful to provide some data regarding the durability of the system. In particular, the authors are asked to justify the choice not to use a layer acting as an ion-storage layer. Did the authors observe irreversible intercalation in the FTO layer during the bleaching phase? It is not clear if the size of ECDs is 30 cm or 70 cm.

In the Results section, at line 100, the authors should report precise values for blocking of UV and NIR radiation, using metrics from EN 410, e.g.

At line 269, the authors revealed the control strategy, base on irradiance (indoor or outdoor?). Nevertheless, in the supporting information, when they describe the setting of the tests, an illuminance-based approach is adopted. The authors should clarify this point.

More details about the EnergyPlus model setting should be reported. It is not clear how the energy savings were estimated.

The results of the work should be described in greater detail. For example, a bar chart is presented showing the results obtained from the simulations for different locations, but these results are not described exhaustively.

Reviewer #2

(Remarks to the Author)
The authors report about an electrochromic system able to provide controlled shading to greenhouses, in order to provide

internal adaptive photo and thermal regulation.
The results, tested for lettuce growing, look promising for a mainly unexplored application of electrochromic technology. These findings can be interesting and innovative in the field, but some concerns arise in order to consider the manuscript as a sound reference. Let me comment on that.

1. There is a critical aspect that should be considered and commented about the capabilities of these devices to modulate solar spectrum. The problem is that, presently, they cannot modulate UV nor NIR. This is because glass and FTO, main components of the device, act as passive filters in those ranges. This limits the functionality of the devices to Visible range. The authors should clearly state these limitations and discuss them.
2. This relates with a second concern, and is about the extension to which this technology can be applied for different climate conditions. The authors claim that this is an "all-weather" solution, which is clearly arguable. The proposed architecture is mainly useful for hot climates, but probably counter-productive in warm or cold climates. Blocking NIR range in cold seasons could be damaging to the crop, as this radiation is needed to heat the inner space of the greenhouse. In the worst case, additional heating will be needed, with additional energy costs. Th authors should not claim that this is an "all-weather" solution.
3. Regarding the electrochromic material chosen, I would say WO3 is not probably the best option. A tailored material that does not absorb red or blue ranges could be beneficial (the authors point to this direction too, lines 332-334. In any case I don't think this is critical at this moment and this material can be used as a proof of concept.
4. Climate modelling. As I mentioned before, the proposed configuration won't be practical for warm or cold climates. Indeed, when performing the analysis of energy savings, just the hot season is taking into consideration for those climates. This is not a valid analysis: an all-year analysis should be performed in these climates. Energy savings in hot season could be accompanied by the need of heating energy costs during cold season (due to NIR blocking) and consequently result in net savings below Zero.

In view of these concerns, I recommend major revision of the manuscript.

Additional comments or corrections:
5. Less critical, but please also consider it: Personally, I don't understand the use of "urban farming" along the text. The solution is clearly applicable to greenhouses and from my point of view this is the correct term to use.
6. Line 65: please correct this sentence "…ensuring reliable operations under realistic conditions…". Be aware that assuring a two-months stability period in a device does not guarantee reliable operation under realistic conditions. Realistic conditions in agricultural greenhouses would probably require several years of operation.
7. Lines 67, 68, 94: please avoid stating that the device modulates solar spectrum, specially in the UV or NIR ranges. It only modulates visible range, and just block the rest.
8. Electrochromic device design: In the proposed architecture, FTO is used as simultaneously as counter electrode and substrate. No ion-storage material is used. In the past, these configurations have been reported to result in irreversible degradation of FTO (or ITO) visible through a browning of the substrate. The authors should comment on these aspects related to their own devices.
9. Supplementary fig 3. It would be convenient to add the initial performance of the devices so it can be compared with the after two-month test.
10. Supplementary fig 9. It is not clear what is meant with "across diverse weather conditions". What is exactly shown in the graphs? Please specify.
11. Line 379. Please correct (-3.5 V/ 2.0 V).
12. Line 371. Please correct "is detailed introduced"

Reviewer #3

(Remarks to the Author)
Manuscript ID: NCOMMS-26-004207-T
Title: Scalable all-weather electrochromic shading for adaptive photothermal regulation in urban agriculture
The manuscript presents a research study proposing the use of a self-powered electrochromic device to regulate temperature and incoming radiation in order to improve lettuce production and quality. A comprehensive characterization is provided, including structural and optical analyses, together with a full implementation of the proposed device. The manuscript also addresses applicability under different climatic conditions and scalability aspects through simulations, further supporting the practical relevance of the proposed approach. The results clearly demonstrate the suitability of the approach for the intended application and, in my view, represent outstanding outcomes, supported by very clear and well-designed figures, graphs, and photographs. Therefore, I recommend the manuscript for publication after minor revisions.
Please find below some specific comments and queries:
1. Some acronyms are not defined or are inconsistently defined throughout the manuscript. Please verify and clarify, namely "EC", "DLI", and "Ref".
2. The scientific name of lettuce appears twice using different formats. Please revise for consistency.
3. In Figure 1, the caption could be improved for clarity. In particular, the description of Figure 1d should more clearly guide the reader, explicitly referring to the left panel and to the colour graphs (left, centre, and right). Moreover, in the left graph of Figure 1d, additional data corresponding to the use of PE film only should be included, similarly to what is presented in the right graph.
4. In line 193, the authors state that the DLI under EC shading was 10 mol m^{-2} day^{-1}; however, this value corresponds only some of the tested days. It should be clearly stated that the DLI under EC conditions fell within the recommended range only on a limited number of test days, although it remained closer to the optimal range than in the outdoor or Ref conditions.

5. In Figure 3, for improved readability, I recommend adding visual guides (e.g., dashed lines or shaded regions) indicating the optimal values or ranges for crop production, since the current description of this figure is somewhat dense and extensive.

Version 1:

Reviewer comments:

Reviewer #1

(Remarks to the Author)
Dear Authors
after this round of revisions, your manuscript has been improved and, in my opinion, canbe considered suitable for publication in this journal.
Kind regards.

Reviewer #2

(Remarks to the Author)
I congratulate the authors for the great effort put towards addressing all reviewer comments. The manuscript has been considerably improved. From my side, I consider that all my comments have been adequately responded, and the manuscript is ready for publication.

Reviewer #3

(Remarks to the Author)
The authors are acknowledged for the efforts on addressing my comments. From my viewpoint, the manuscript can now be accepted.

Open Access This Peer Review File is licensed under a Creative Commons Attribution 4.0 International License, which permits use, sharing, adaptation, distribution and reproduction in any medium or format, as long as you give appropriate credit to the original author(s) and the source, provide a link to the Creative Commons license, and indicate if changes were made.
In cases where reviewers are anonymous, credit should be given to 'Anonymous Referee' and the source.
The images or other third party material in this Peer Review File are included in the article's Creative Commons license, unless indicated otherwise in a credit line to the material. If material is not included in the article's Creative Commons license and your intended use is not permitted by statutory regulation or exceeds the permitted use, you will need to obtain permission directly from the copyright holder.
To view a copy of this license, visit https://creativecommons.org/licenses/by/4.0/

Response Letter

Scalable irradiance-adaptive electrochromic shading for photothermal regulation in agriculture

Dear Reviewers,

We would like to express our sincere thankfulness for your comments and suggestions that have helped improve our manuscript “Scalable irradiance-adaptive electrochromic shading for photothermal regulation in agriculture” (Manuscript ID: NCOMMS-26-004207-T). The reviewers’ comments have been invaluable in improving the quality and clarity of our manuscript. This letter encloses our point-by-point response to the comments raised by the Reviewers. The reviewers’ comments are reproduced in black, whereas our responses are given afterward in blue. In the revised manuscript and supplementary information, the contents that are revised/added have been marked in red.

Reviewer #1 (Remarks to the Author):

The manuscript deals with the integration of Electrochromic devices in greenhouses, investigating the effect on lettuce growth. The topic is indeed interesting, but the manuscript should undergo major revisions before being considered suitable for publication.

We thank the referee for their time and remarks in reviewing our manuscript. We appreciate the constructive comments and suggestions. Listed below are the reviewer's comments and our responses. We hope that we have addressed all of the reviewer's comments.

Comment #1. *In the introduction section, the authors should improve the list of bibliographic references. The bibliographic section does not clarify the positioning of this work with respect to the state of the art. The authors should report a wide comparison with previous works dealing with the same topic. The authors should clarify what constitutes the degree of innovation of the proposed work.*

Response: We thank the reviewer for this important comment. In response, we have substantially revised and reorganized the Introduction: (i) **broadens the bibliographic coverage** and **provides a clearer and wider comparison** with previous agricultural photothermal control strategies, (ii) **defines the current state of the art** and its **key limitations**, and (iii) **clearly positions the innovation of this work**.

Specifically, the revised Introduction now includes a more comprehensive and structured discussion of existing approaches for agricultural light–thermal regulation, including photoselective, photoluminescent, photoconversion, and radiative cooling materials. These approaches are discussed within a unified framework to highlight that, despite their effectiveness, their optical properties are typically static once fabricated, limiting adaptability under fluctuating outdoor irradiance. Building on this comparison, we further clarify the need for adaptive strategies and explicitly position electrochromic (EC) technologies as a dynamically tunable solution. Finally, the revised Introduction explicitly clarifies the innovation of this work in the context of real-world deployment.

To address this comment, the Introduction part has been substantially rewritten and expanded in the **revised Introduction**:

Lines 49-83, Page 2-3

To overcome these limitations, emerging agricultural photothermal control strategies have been developed in heat-intensive regions. Photoselective, photoluminescent, and photoconversion covers enable moderate shading while tailoring spectral composition to crop-specific needs.[10–13] Radiative cooling covers enhance heat dissipation through additional infrared emission.[14–16] Together, these approaches demonstrate that coordinated regulation of light and heat is a viable route for improving crop microclimates.[17,18] Nevertheless, their optical properties are typically fixed once designed and fabricated, limiting adaptability under fluctuating outdoor irradiance. Current photothermal control materials generally either block broad portions of the solar spectrum[19] or transmit PAR within a predefined spectral window,[9,20–22] making it difficult to dynamically maintain crop-optimal photosynthetic and thermal conditions under fluctuating solar irradiance. Thus, the current state of the art in agricultural photothermal control is severely constrained by materials with static optical properties.

These limitations highlight the need for adaptive strategies capable of dynamically regulating both the intensity and spectral quality of PAR while simultaneously suppressing excess heat gain. PAR accounts for nearly 50% of total solar energy and plays a dual role in governing photosynthesis and contributing to thermal load.[23,24] Precise control of PAR is therefore critical for crop performance: excessive irradiance can induce photoinhibition and saturation, whereas insufficient irradiance limits carbon assimilation and growth.[25] Electrochromic (EC) technologies are distinct in this context because they offer reversible and on-demand tuning of solar-spectrum transmission through switching between visible-colored and bleached states, enabling adaptive photothermal regulation.[26,27] In principle, EC shading can simultaneously regulate PAR intensity, modify spectral quality, and suppress heat-inducing radiation in response to changing environmental conditions. This combination of dynamic spectral tunability and real-time environmental responsiveness differentiates EC shading from existing agricultural photothermal-control materials with static optical properties. However, despite this potential, translating EC technologies from lab demonstrations to outdoor agricultural environments remains challenging because it requires simultaneous

achievement of large-area scalability, device durability, and autonomous system-level operation.

Here, we develop a scalable irradiance-adaptive EC shading platform for photothermal regulation in hot-climate urban agriculture. The platform is enabled by robust large-area EC devices with large optical contrast, fast switching, excellent optical memory, and maintains stable electrochromic performance over a two-month outdoor exposure test under tropical conditions. Building on this device-level robustness, we integrate the EC devices into a modular, solar-powered shading system that autonomously switches between bleached and colored states in response to real-time solar irradiance. This enables irradiance-adaptive regulation of visible (PAR) transmission under fluctuating outdoor conditions. It maintains PAR within optimal photosynthetic ranges (Fig. 1a) while suppressing excess radiative heat through effective UV and NIR attenuation. Additionally, state-dependent modulation of PAR quality, particularly the blue-to-red (B/R) light ratio, further supports photosynthesis under varying light conditions.

These revisions provide a clearer comparison with prior work, define the state of the art, and explicitly articulate the innovation of this study as a scalable, adaptive, and field-deployable electrochromic shading platform for agricultural applications.

Comment #2. *With reference to the architecture proposed for the electrochromic device, it would be useful to provide some data regarding the durability of the system. In particular, the authors are asked to justify the choice not to use a layer acting as an ion-storage layer. Did the authors observe irreversible intercalation in the FTO layer during the bleaching phase? It is not clear if the size of ECDs is 30 cm or 70 cm.*

Response: We thank the reviewer for this insightful comment. **In response, we have (i) added quantitative durability data and clarified the degradation mechanism, (ii) explained the rationale for not incorporating a dedicated ion-storage layer together with supporting control-experiment evidence, and (iii) clarified the device size and module configuration** in the revised manuscript.

(i) Durability data and degradation mechanism

To address the durability of the system, we have added a direct comparison of the transmittance–time (T–t) response during the initial cycles and after 1000 cycles (**Fig. R1b (Revised Supplementary Fig. 4)**), together with the corresponding cycling performance (Fig. R1a). This comparison shows that the degradation is mainly associated with a decrease in bleached-state transmittance after cycling, whereas the colored-state transmittance changes much less markedly, indicating that the long-term optical degradation mainly originates from the bleaching process.

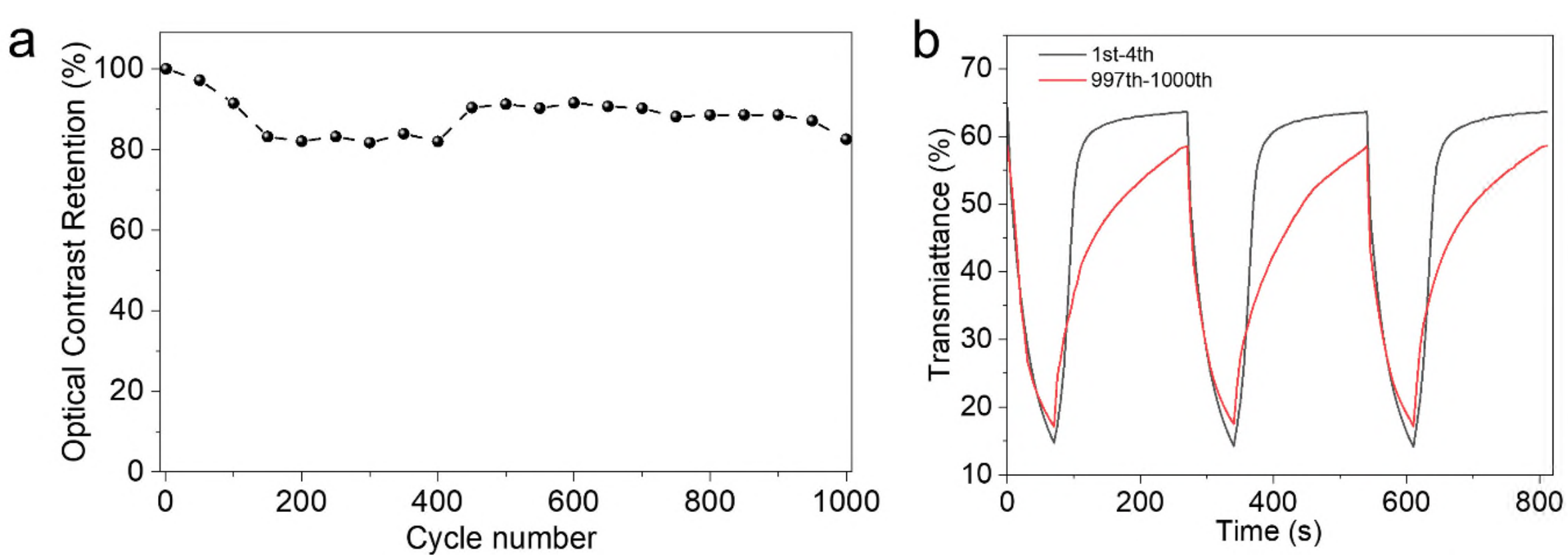

Fig. R1 (Revised Supplementary Fig. 4). **a,** Cycling performance of the ECD. **b,** the transmittance–time response during initial cycles and after 1000 cycles of the 30 × 30 cm^2 ECD

The decrease in bleached-state transmittance after repeated cycling is consistent with limited charge reversibility and irreversible ion intercalation at the bare FTO counter electrode, which acts as the ion-storage side in the current device architecture. In the absence of a dedicated ion-storage layer, the FTO substrate participates in charge compensation during cycling. Although FTO can function as a simplified counter electrode in EC devices,[1] its ion-storage capacity and charge-

balance capability are limited compared with those of purpose-designed ion-storage electrodes.[2,3] As a result, irreversible ion accumulation at the FTO side during repeated cycling can reduce bleaching efficiency and ultimately limit device stability.

(ii) Ion-storage layer, architectural rationale, and supporting experiment.

From an application-oriented perspective, the primary objective of this work was to establish a scalable, outdoor-deployable EC shading platform for a pilot greenhouse cultivation demonstration. Within this context, the present large-area ECD configuration already delivers the key performance metrics required for greenhouse operation, including substantial optical contrast (51% at 633 nm), long optical memory (>21600 s), stable cycling performance (82.5% retention after 1000 cycles), and reliable outdoor operation over two months under tropical conditions. These results demonstrate that the current device architecture is sufficient to support the intended proof-of-concept for adaptive photothermal regulation.

Nevertheless, we fully agree with the Reviewer that the incorporation of a dedicated ion-storage/counter-electrode layer is important for further improving charge balance, reducing operating voltage, and enhancing long-term durability. This point has been clarified in the revised manuscript, and the Discussion section has been expanded accordingly.

To further examine this architectural limitation, we performed a supporting control experiment using the same 30 × 30 cm^2 printed WO_3 electrode and the same electrolyte, but with an added currently under-development large-area PEDOT:PSS-based composite ion-storage electrode. Under otherwise comparable conditions, the ion-storage layer-based device achieved a similar optical contrast (50.0% at 633 nm) while operating at substantially reduced voltages (-2.0 V for 60 s / 0.1 V for 100 s, compared with -3.5 V for 70 s / 2.0 V for 200 s for the bare-FTO architecture), and exhibited improved cycling stability (91% optical-contrast retention after 1000 cycles, compared with 82.5% retention after 1000 cycles for the bare-FTO architecture) **(Fig. R2)**. These results further support the Reviewer's point and confirm that counter-electrode design plays a critical role in determining long-term device performance.

Because this ion-storage-electrode-based architecture is still under active development and optimization for multiple performance metrics relevant to outdoor EC shading (e.g., optical memory and environmental stability), it was not incorporated into the main device platform of the present study.

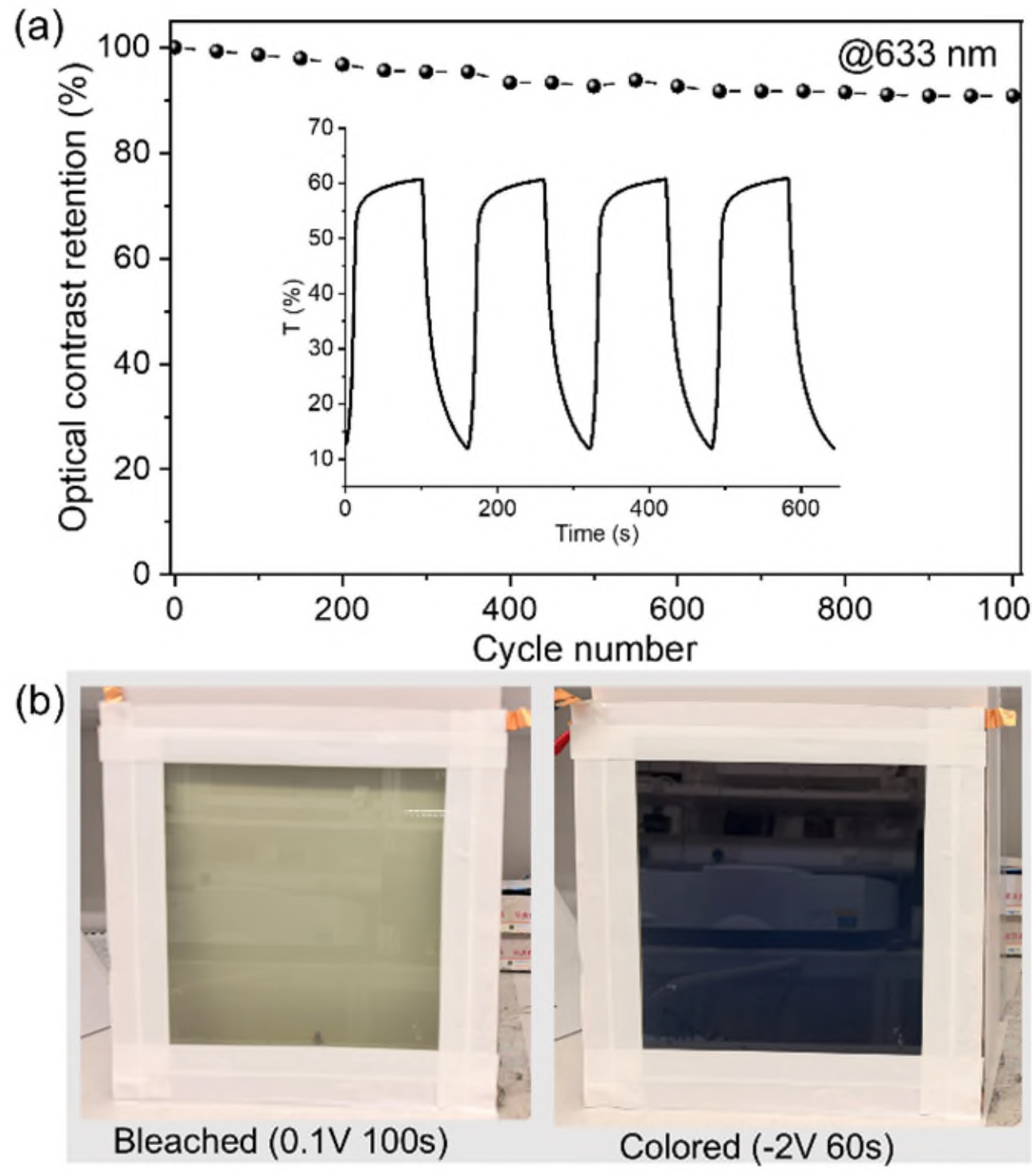

Fig. R2. Supporting control experiment using an added large-area ion-storage electrode. **a**, Optical-contrast retention at 633 nm over 1000 coloration/bleaching cycles for a 30 × 30 cm² ECD fabricated using the same printed WO_3 working electrode and the same electrolyte, with an added large-area PEDOT:PSS-based composite ion-storage electrode. The inset shows the corresponding transmittance–time response during initial representative cycles. **b**, Photographs of the device in the bleached state (0.1 V, 100 s) and colored state (−2.0 V, 60 s).

(iii) Device size clarification.

We apologize for any confusion regarding the device size. The individual ECD size is 30 × 30 cm^2. The larger 0.7 × 0.7 m^2 unit refers to the EC shading module used for outdoor field experiments, which consists of a 2 × 2 array of four 30 × 30 cm^2 ECD units mounted on a supporting frame. This has been clearly clarified in the revised manuscript.

To address this comment, we have revised the manuscript to:

1. Add a comparison of the initial and post-1000-cycle T–t responses as Supplementary Fig. 4;

2. Revise the stability discussion to state that the reduced bleached-state transmittance after cycling is consistent with limited charge reversibility and irreversible ion intercalation at the bare FTO counter side:

Lines 165-170, Page 6

Comparison of the initial and after-cycling transmittance–time responses (Supplementary Fig. 4) further indicates that the degradation is mainly associated with a decrease in bleached-state transmittance, whereas the colored-state transmittance changes less markedly. This behavior is consistent with limited charge reversibility and irreversible ion intercalation at the bare FTO counter side during repeated bleaching/coloration processes, as reported for bare FTO/ITO-based counter electrode configurations.[37,38]

3. Expand the **Discussion** part to acknowledge the architectural limitations of the current device and outline the future need for scalable ion-storage electrode materials to improve the long-term stability of large-area EC devices:

Lines 392-396, Pages 15-16

Building on the above scope and limitation, several directions can further advance this technology. First, future device architectures can incorporate ion-storage electrode materials with electrochemical characteristics appropriately matched to WO_3, improving charge balance, reducing operating voltage, and further enhancing the long-term stability of large-area EC devices while remaining compatible with large-area and low-temperature fabrication.

4. State that the 0.7×0.7 m^2 modular EC shading system is a 2×2 array of 30×30 cm^2 ECD units mounted on a supporting frame:

Line 18, Page 1

Building on this device-level robustness, a modular 0.7×0.7 m^2 solar-powered EC shading system (2×2 array of 30×30 cm^2 EC device units) autonomously regulates photosynthetically active radiation (PAR) intensity

Lines 191-193, Page 8

Leveraging the satisfactory electrochromic performance of these scalable and parallel ECDs, we deployed them as EC shading in a meter-scale (0.7×0.7 m^2, 2×2 array of 30×30 cm^2 ECD units mounted on a supporting frame) plant-growing testbed for outdoor field experiments in Singapore.

References:

1. Perez, I., Sosa, V., Gamboa, F., Enriquez-Carrejo, J. L. & Mixteco Sanchez, J. C. Role of lithium intercalation in fluorine-doped tin oxide thin films: Ab initio calculations and experiment. *The Journal of Chemical Physics* **156**, 094701 (2022).
2. Wu, L., Fang, H., Li, J., Wu, L. & Wang, H. Catalyzing Viologen Electrochromic Reaction with CuOx Nanoparticles. *Advanced Materials Technologies* **8**, 2301028 (2023).
3. Jo, M.-H., Koo, B.-R., Kim, K.-H. & Ahn, H.-J. Tailored interface stabilization of FTO transparent conducting electrodes boosting electron and Li ion transport for electrochromic energy-storage devices. *Chemical Engineering Journal* **431**, 134036 (2022).

Comment #3. *In the Results section, at line 100, the authors should report precise values for blocking of UV and NIR radiation, using metrics from EN 410, e.g.*

Response: We appreciate the reviewer's valuable suggestion. In response, we have added quantitative UV and NIR blocking values using EN 410-type metrics with the clarified calculation methodology, and incorporated these standardized metrics directly into the main text to support the corresponding statements.

We have quantified the UV, VIS, and NIR blocking performance of the ECD using EN 410-type band-integrated transmittance metrics, calculated from the measured spectral transmittance (T(λ)) with spectral weighting using the following equations[4]:

$$T_{UV} = \int_{280}^{400} T(\lambda)\phi_{sol}(\lambda)d\lambda \,/ \int_{280}^{400} \phi_{sol}(\lambda)d\lambda \quad (1)$$

$$T_{VIS} = \int_{400}^{780} T(\lambda)\phi_{VIS}(\lambda)d\lambda \,/ \int_{400}^{780} \phi_{VIS}(\lambda)d\lambda \quad (2)$$

$$T_{NIR} = \int_{780}^{2500} T(\lambda)\phi_{sol}(\lambda)d\lambda \,/ \int_{780}^{2500} \phi_{sol}(\lambda)d\lambda \quad (3)$$

where T(λ) represents the transmittance at wavelength λ, $\phi_{vis}(\lambda)$ and $\phi_{sol}(\lambda)$ are the standard efficiency functions for the photopic vision and the solar irradiance spectrum for an air mass of 1.5. The resulting T_{UV}, T_{VIS} and T_{NIR}, together with the corresponding blocking values, are summarized in Supplementary Table 1 for the colored and bleached states.

Table. R1 (Revised Supplementary Table 1). Band-integrated UV/VIS/NIR transmittance and blocking (EN 410-type metrics)

State	T_{UV}(280-400 nm)	UV blocking	T_{VIS}(400-780 nm)	VIS blocking	T_{NIR} (780-2500 nm)	NIR blocking
Bleached	18.2%	81.8%	57.6%	42.4%	22.4%	77.6%
Colored	16.3%	83.7%	24.0%	76%	3.7%	96.3%

Based on these calculations, the ECD exhibits strong UV suppression in both optical states, blocking 83.7% (colored) and 81.8% (bleached) of UV radiation. For the NIR region, the ECD blocks 96.3% (colored) and 77.6% (bleached) of the integrated NIR radiation. The relatively low NIR transmittance in both states is mainly governed by the intrinsic filtering effect of the FTO-glass substrates. A single FTO-glass already shows substantial attenuation (Fig. R3, integrated transmittance: T_{uv}=48.1%, T_{vis}=76.5%, T_{NIR}=47.9%), and this effect is further strengthened in the

device due to the two FTO-glass panes (FTO-glass/WO_3/electrolyte/FTO-glass), resulting in a multiplicative reduction in UV/NIR transmission.

Under this configuration, UV attenuation is dominated by passive filtering of the FTO-glass substrates, whereas NIR attenuation arises from the combined effect of substrate filtering and additional electrochromic modulation. As a result, the NIR transmittance remains relatively low in both states, and the switching-induced modulation is relatively limited (~18.7%), compared with the more actively tunable modulation in the visible (PAR) range.

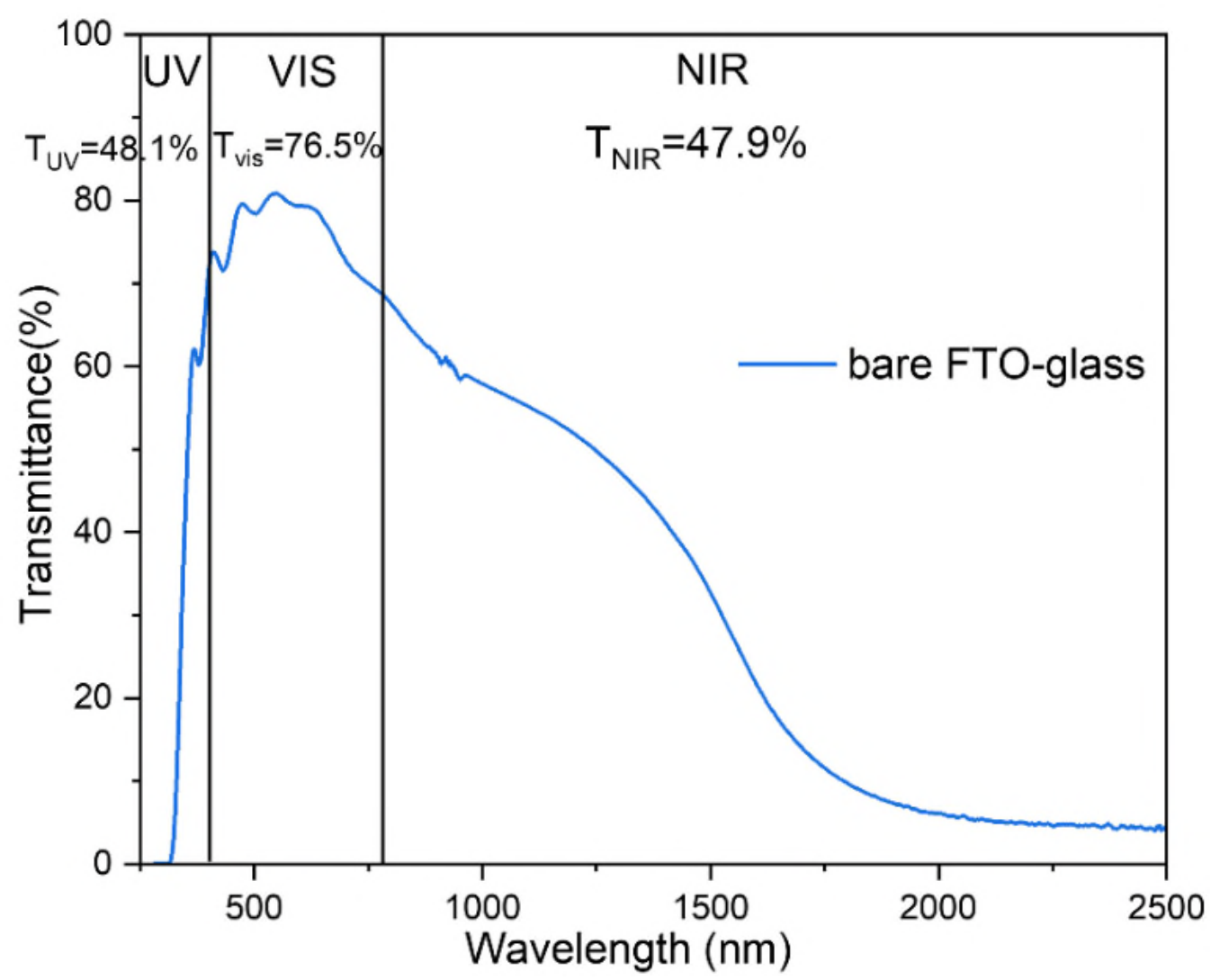

Fig. R3 (Revised Supplementary Fig. 1) Bare FTO-glass spectral transmittance and band-integrated transmittance value

Therefore, the revised text now clarifies that UV suppression is strong and largely state-independent, while NIR attenuation is significant in both states and primarily governed by the FTO glass baseline, with additional moderate switchable electrochromic modulation.

To address this comment, we have revised the manuscript to:

1. Add the quantitative UV- and NIR-blocking values to support and strengthen the statement "EC shading in both colored and bleached states effectively blocks UV and NIR radiation":

Lines 104-113, Page 4

As shown in Fig.1d (centre and right), the ECD in both colored and bleached states strongly suppresses UV transmission and maintains low NIR transmittance, while providing switchable visible light transmission. Mechanistically, UV suppression is dominated by the passive filtering of the FTO-glass substrates (Supplementary Fig. 1), whereas NIR attenuation is also substantial in both states and primarily governed by the FTO-glass baseline, with additional moderate

electrochromic modulation. These effects are quantified for the complete device using EN 410-type band-integrated metrics (Supplementary Table 1): the ECD blocks 83.7%/81.8% (colored/bleached) of UV radiation (280–400 nm), and attenuates 96.3%/77.6% (colored/bleached) of NIR radiation (780–2500 nm), respectively. This spectral attenuation is further reflected in the reduced transmitted solar energy across these regions, leading to a lower radiative heat load (Supplementary Table 2).

2. Provide the calculation details (EN 410-type integrated metrics and equations) and the results for colored/bleached states in the **Supplementary Information** (Supplementary Table 1 and accompanying text).

Supplementary Table 1. Band-integrated UV/NIR transmittance and blocking

State	T_{UV}(280-400 nm)	UV blocking	T_{VIS}(400-780 nm)	VIS blocking	T_{NIR} (780-2500 nm)	NIR blocking
Bleached	18.2%	81.8%	57.6%	42.4%	22.4%	77.6%
Colored	16.3%	83.7%	24.0%	76%	3.7%	96.3%

Band-integrated transmittances for the ECD in colored and bleached states were calculated from the measured spectral transmittance T(λ) following the EN 410 weighted-integration framework with the following equations[6]:

$$T_{UV} = \int_{280}^{400} T(\lambda)\phi_{sol}(\lambda)d\lambda \,/\, \int_{280}^{400} \phi_{sol}(\lambda)d\lambda \quad (4)$$

$$T_{VIS} = \int_{400}^{780} T(\lambda)\phi_{VIS}(\lambda)d\lambda \,/\, \int_{400}^{780} \phi_{VIS}(\lambda)d\lambda \quad (5)$$

$$T_{NIR} = \int_{780}^{2500} T(\lambda)\phi_{sol}(\lambda)d\lambda \,/\, \int_{780}^{2500} \phi_{sol}(\lambda)d\lambda \quad (6)$$

Where T(λ) represents the transmittance at wavelength λ, $\phi_{vis}(\lambda)$ and $\phi_{sol}(\lambda)$ are the standard efficiency functions for the photopic vision and the solar irradiance spectrum for an air mass of 1.5.

References:

4. de Normalisation, C. E. Glass in Building–Determination of Luminous and Solar Characteristics of Glazing. *Brussels (Belgium): CEN, Publication No. EN* **410**, 64 (2011).

Comment #4. *At line 269, the authors revealed the control strategy, based on irradiance (indoor or outdoor?). Nevertheless, in the supporting information, when they describe the setting of the tests, an illuminance-based approach is adopted. The authors should clarify this point.*

Response: We thank the reviewer for this important comment. To directly address the question: the simulation (EnergyPlus EMS) control strategy uses outdoor solar irradiance (W/m^2) as required by the model input, whereas the pilot (field) system uses under-shade illuminance (lux) control strategy due to sensor limitations. Although different physical quantities are used, both control strategies implement the same PAR-based switching logic and are quantitatively linked.

In response, we have (i, ii) explained the rationale for using irradiance in the simulation control system and using illuminance in the pilot system, (iii) clarified the definition and validation of the pilot PAR and illuminance threshold variables setting, and (iv) clarified the connection between the outdoor pilot PAR control and simulation irradiance control variables.

(i) Simulation control (EnergyPlus EMS): outdoor solar irradiance in W/m^2

At line 269, the switching thresholds (530 and 380 W/m^2) refer to the outdoor incident solar irradiance used by the EnergyPlus energy management system (EMS) to trigger the electrochromic state change. This is consistent with the EMS input variable ("Solar Radiation Rate per Area [W/m^2]", and therefore the control thresholds are defined in terms of outdoor irradiance.[5]

(ii) Field control (pilot system): illuminance (lux) measured under the EC shading

In the pilot system, real-time switching is based on an illuminance (lux) sensor positioned under EC shading. This design is necessary because the sensor (BH1750FVI) has a limited measurement range (1-65535 lx)[6] and would saturate under full outdoor light before reaching the intended switching threshold (~1240 $\mu mol/m^2/s$). As 65535 lx corresponds to ~1212 $\mu mol/m^2/s$ using a standard adopted conversion factor (PPFD=0.0185×lux) for sunlight.[7] Thus, under-shade illuminance is used as a practical proxy for real-time control, while PAR is simultaneously recorded for validation.

(iii) Definition and validation of the pilot PAR and illuminance threshold variables setting (target: maintain PAR under EC shading within 250–750 $\mu mol/m^2/s$)

The dual-threshold strategy was designed to maintain the PAR value under EC shading within the crop-optimal window for lettuce (250–750 $\mu mol/m^2/s$). Specifically, when the ECD was in the bleached state and transmitted PAR exceeded ~750 $\mu mol/m^2/s$, the controller switched the device to the colored state; the corresponding illuminance at the switching event was ~27000 lx (set as

the coloration threshold). Conversely, when the ECD was in the colored state and transmitted PAR dropped below ~250 μmol/m^2/s, the controller reverted the ECD to the bleached state; the corresponding under-shade illuminance was ~11000 lx (set as the bleaching threshold). The flowchart in Fig. R4 (Revised Supplementary Fig. 14) clearly demonstrates this dual-threshold setting method in the light sensor-controlled system.

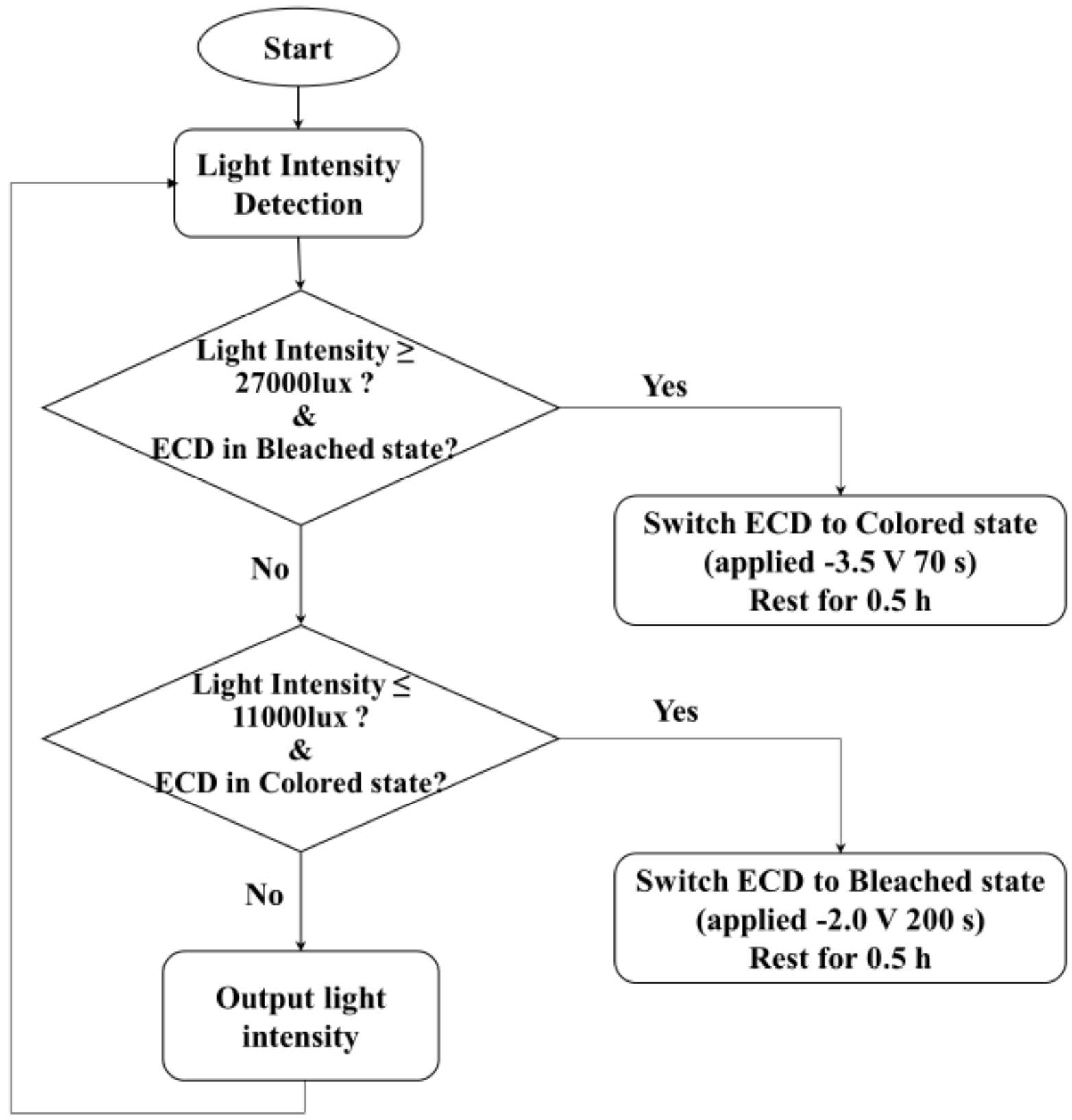

Fig. R4 (Revised Supplementary Fig. 14). The flowchart with the setting parameters of the circuit in the light sensor-controlled system

We note that the illuminance sensor was sampled every second, whereas the PAR sensors were logged every 2 minutes. Because the electrochromic switching durations (70 s for coloration and 200 s for bleaching) are on the same order as the PAR logging interval (120 s), the under-shade PAR records may not capture the exact onset of each switching event with high temporal precision. Importantly, the outdoor PAR time series provides an independent reference, as it is not affected by the EC switching process. Therefore, the outdoor PAR values (~1240 and ~890 μmol/m^2/s) reported correspond to the nearest recorded data points around the switching events. By examining multiple representative coloration and bleaching events in the PAR time-series map (Fig. 3b), we confirm that these outdoor PAR values are consistent with the intended switching conditions at Fig. R5.

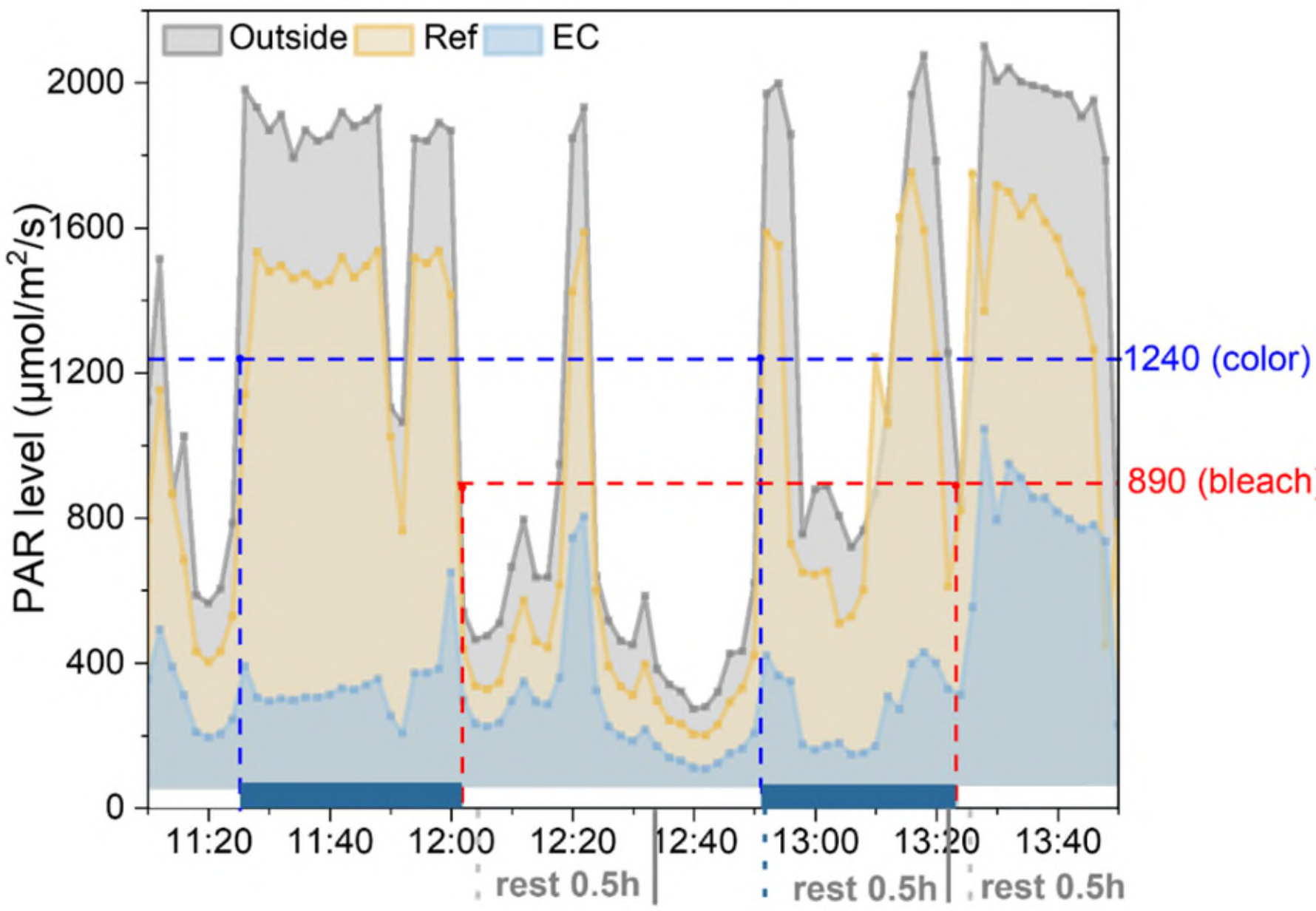

Fig. R5. PAR values of the EC/Ref testbeds and outside environment (11:00-13:50, October 24, 2024). The EC testbed is operated under the light sensor-controlled system, and different colored bottom bars indicate different working states (white bar: bleached state, blue bar: colored state), the rest time are point for clarification.

(iv) Connection between the outdoor PAR and simulation irradiance control variables.

To ensure consistent switching logic between experiment and simulation, we converted the outdoor PAR reference thresholds (1240 and 890 μmol/m^2/s) into outdoor irradiance thresholds (530 and 380 W/m^2). This conversion follows literature-reported relationships between PPFD and global solar irradiance: PPFD can be converted to PAR radiant flux using a typical factor of ~ 4.6 μmol/J,[8] and PAR accounts for ~50% of total solar energy under representative spectra.[9] This provides an approximate conversion from outdoor PPFD (μmol/m^2/s) to the global solar irradiance (W/m^2) thresholds required by the EnergyPlus EMS.

In summary, the pilot system uses under-shade illuminance (lux) thresholds for practical real-time control, whereas the simulation uses outdoor incident solar irradiance (W/m^2) as required by the EMS input variable; these control variables are quantitatively linked through the measured outdoor PAR reference points and the PPFD-to-irradiance conversion. The corresponding threshold parameters are summarized in **Table R2 (Revised Supplementary Table 4)**.

Table R2 (Revised Supplementary Table 4). Switching condition parameters of the electrochromic shading system (under-ECD illuminance/PAR/energy irradiance, and corresponding outdoor levels)

Trigger point	Parameter	Outside	Under Bleached EC	Under Colored EC
Bleach-color	Illuminance (lux)	-	27000	-
	PPFD (μmol/m²/s)	1240	750	-
	Global solar energy irradiation (W/m^2)	530	-	-
Color-bleach	Illuminance (lux)	-	-	11000
	PPFD (μmol/m²/s)	890	-	250
	Global solar energy irradiation (W/m^2)	380	-	-

To address this comment, we have revised the manuscript and supplementary information:

1. Clarified the pilot control strategy as a lux-based system operating under EC shading, with PAR-based threshold design stated:

Lines 196-198, Page 8

> In the EC testbed, the ECD optical state was autonomously controlled by a solar-powered sensing and control unit to maintain PAR within the optimal range (250-750 μmol/m²/s) for lettuce (Green Span) growth.[39,40] For the pilot system, the real-time switching was triggered by a light sensor (lux) positioned under the EC shading, with corresponding EC PAR levels and outdoor PAR threshold levels summarized in Supplementary Table 4.

2. Clarified that the simulation control uses outdoor irradiance (W/m^2) consistent with EnergyPlus EMS input variables and defined switching thresholds:

Lines 294-298, Page 12

> Dynamic EC shading was implemented through an energy management system (EMS) using predefined outside incident solar irradiance thresholds on the roof surface (Supplementary Note 1 and Supplementary Tables 6): the EC shading switched to the colored state when outside solar irradiance exceeded 530 W/m^2 and reverted to the bleached state when outside solar irradiance fell below 380 W/m^2.

3. Added a clear explanation of the mapping between illuminance, PAR, and irradiance, including threshold definitions and validation, and updated **Supplementary Fig. 14** and **Supplementary Table 4** to document this workflow and parameter relationships:

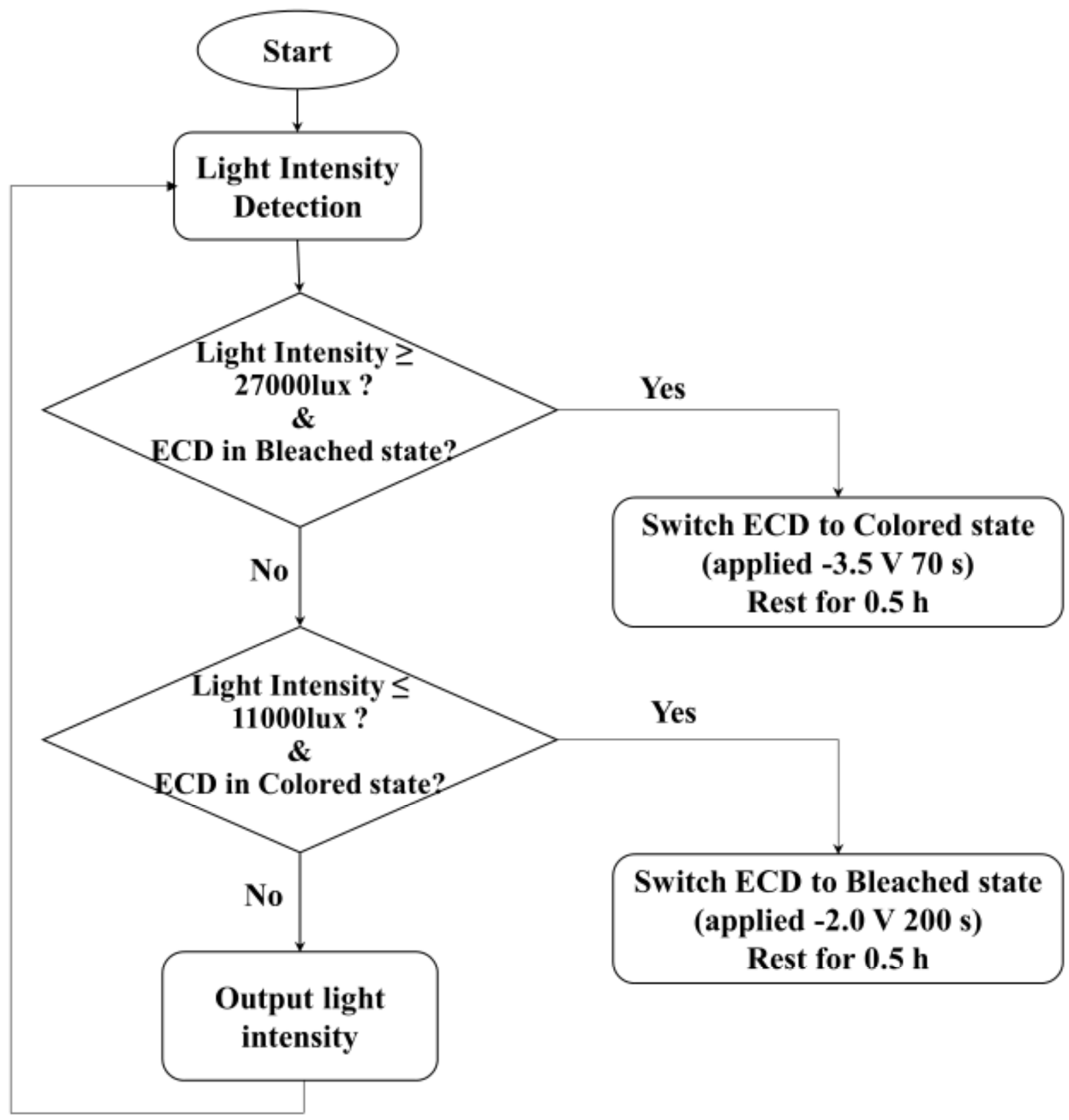

Supplementary Fig. 14. The flowchart with the setting parameters of the circuit in the light sensor-controlled system

The light sensor was set below the electrochromic shading. When the light intensity is higher than 27000 lux (equivalent to 750 μmol/m²/s under bleached electrochromic shading according to our pilot test) and the ECDs are in the bleached state, the ECD will switch to the colored state. When the light intensity is lower than 11000 lux (equivalent to 250 μmol/m²/s under colored electrochromic shading according to our pilot test) and the ECDs are in colored state, the ECD will switch to bleached state. To adapt to the fluctuating weather conditions, we set a 0.5 h rest time for efficient device operation after every switch. The light sensor was detected every second for control, whereas the PAR sensors were logged every 2 minutes; therefore, outdoor PAR was used as an independent reference and threshold to validate switching points.

Supplementary Table 4. Switching condition parameters of the electrochromic shading system (under-ECD illuminance/PAR/energy irradiance, and corresponding outdoor levels)

Trigger point	Parameter	Outside	Under Bleached EC	Under Colored EC
Bleach-color	Illuminance (lux)	-	27000	-
	PPFD (µmol/m²/s)	1240	750	-
	Global solar energy irradiation (W/m^2)	530	-	-
Color-bleach	Illuminance (lux)	-	-	11000
	PPFD (µmol/m²/s)	890	-	250
	Global solar energy irradiation (W/m^2)	380	-	-

To better set up the switching condition parameters of the electrochromic shading, we mapped the real-time control signal (under-shade illuminance, lux) to the crop-relevant transmitted PAR thresholds and the corresponding outdoor reference levels. In the outdoor pilot test, switching was triggered using the under-shade illuminance thresholds (27000 lx for coloring and 11000 lx for bleaching), because the light sensor provides high-frequency lux readings for real-time control. The transmitted PAR thresholds were selected to maintain the PAR value within the optimum range of 250 to 750 µmol/m²/s for leafy greens, such as lettuce,[7,8] the system is operated according to the following logic: when the PAR value of the testbed under bleached ECD exceeds 750 µmol/m²/s, the ECD will be triggered to the colored state. In this situation, the PAR value of the outside (sunlight) is recorded as 1240 µmol/m²/s. When the PAR value of the testbed under colored ECD is lower than 250 µmol/m²/s, the ECD will be triggered to the bleached state. In this situation, the PAR value of outside (sunlight) is recorded as 890 µmol/m²/s.

The recorded corresponding outdoor PAR values function as independent references for validating switching events.

For EnergyPlus simulation, equivalent switching logic was implemented using the outdoor irradiance variable required by the EMS. Outdoor PAR (PPFD) was converted to outdoor global solar irradiance using literature-based approximations: PPFD was first converted to PAR radiant flux using a typical factor of ~4.6 μmol/J,[9] and PAR was assumed to represent ~50% of the total broadband solar energy under representative solar spectra.[10] This yields the irradiance thresholds of 530 W/m^2 (for coloration) and 380 W/m^2 (for bleaching). We note that this conversion is approximate and may vary with spectral distribution; however, it provides a consistent mapping for implementing the irradiance-based EMS switching logic.

References:

5. https://energyplus.net/assets/nrel_custom/pdfs/pdfs_v23.1.0/OutputDetailsAndExamples.pdf

6. https://x2robotics.ca/bh1750fvi-digital-light-intensity-sensor

7. Pedersen, O., Colmer, T. D., Borum, J., Zavala-Perez, A. & Kendrick, G. A. Heat stress of two tropical seagrass species during low tides – impact on underwater net photosynthesis, dark respiration and diel in situ internal aeration. *New Phytologist* **210**, 1207–1218 (2016).

8. Arst, H., Erm, A., Kangro, K., Nõges, T. & Nõges, P. Comparison of spectral and broad-band models for computing photosynthetically absorbed radiation in turbid waters. *Boreal environment research* **11**, 55 (2006).

9. Amthor, J. S. From sunlight to phytomass: on the potential efficiency of converting solar radiation to phyto-energy. *New Phytologist* **188**, 939–959 (2010).

Comment #5. *More details about the EnergyPlus model setting should be reported. It is not clear how the energy savings were estimated.*

Response: We thank the reviewer for this comment. To directly address this concern, we have expanded the description of the global cultivation simulation and provided the full model documentation of cooling-energy savings in **Supplementary Note 1**: daylighting timestep outputs were used to quantify the light regulation and cooling demand was evaluated through EnergyPlus zone heat-balance calculations under IdealLoads control. The corresponding parameter tables (**Supplementary Tables 5-7**) summarize: (i) envelope optical/thermal properties, (ii) EMS control thresholds and simulation settings, (iii) illuminance sampling configuration and post-processing, and (iv) exact definitions used to compute monthly/annual cooling-energy demand and savings. Under those conditions, the only differences between Ref roof shading and EC roof shading in cooling-energy demand arise from the roof optical properties and EC switching control.

Accordingly, in the **Results** section (*Global modelling of light regulation and cooling-energy savings with EC shading*), we have stated the EMS-based EC switching logic using outdoor incident solar irradiance thresholds (530/380 W/m^2), the illuminance sampling strategy (0.6–3.0 m height, 0.16 m interval, N = 16) based on daylighting calculations. We also clarify that cooling demand was evaluated using a ZoneHVAC with a constant 24 °C setpoint.

In the **Methods** section (*EnergyPlus simulation of the light regulation and cooling effect for electrochromic shading*), we further specify the key simulation inputs (Singapore TMY weather file, 10-min timestep, roof/wall optical and thermal properties). Critically, we state that cooling-energy was obtained directly from the EnergyPlus output variable "Zone Ideal Loads Supply Air Total Cooling Energy [J] (TimeStep)" and aggregated to monthly and annual totals to calculate the energy savings between EC and Ref cases:

Lines 515-537, Pages 19-20

> Dynamic EC switching was implemented via the EnergyPlus Energy Management System (EMS) using outdoor incident solar irradiance on the roof surface (sensor variable: *Surface Outside Face Incident Solar Radiation Rate per Area*). The EC roof switched to the colored state when incident irradiance exceeded 530 W/m^2 and reverted to the bleached state with relevant optical properties when outside incident solar irradiance dropped below 380 W/m^2, retaining the previous state between thresholds (Supplementary Note 1; Supplementary Table 6).

To evaluate light regulation within the crop-growing region, illuminance sampling points were placed vertically from 0.6 m to 3.0 m above the floor at 0.16 m intervals (N = 16; Supplementary Table 7). EnergyPlus daylighting calculations were used to obtain timestep illuminance (lux) at each point, accounting for both transmitted daylight and internal inter-reflections within the enclosure. The zone-mean illuminance was calculated and converted to PAR values (Supplementary Note 1).

Cooling-energy demand was quantified using a ZoneHVAC:IdealLoadsAirSystem with a constant cooling setpoint of 24 °C. From a physical perspective, radiative energy incident on the crop canopy is ultimately partitioned into sensible heat (via convective exchange with the air) and latent heat (via evapotranspiration),[69,70] which together represent the thermal load that must be removed to maintain the temperature setpoint. In this study, this partition is used as a mechanistic framework for interpreting the cooling demand rather than as an explicitly prescribed latent load. Cooling energy was obtained directly from the EnergyPlus output variable Zone Ideal Loads Supply Air Total Cooling Energy [J] (TimeStep) and aggregated to monthly/annual totals for comparison between EC and Ref cases (Supplementary Note 1). To account for additional effective radiative heat influence from surrounding heated surfaces (e.g., enclosure and ground), a supplementary radiative heat-input term was implemented as an EMS-controlled radiant internal gain, applied identically in both EC and Ref simulations to preserve comparability (Supplementary Note 1).

Together, these revisions provide a clear and reproducible description of the EnergyPlus model configuration and the cooling-energy saving calculation procedure requested by the reviewer.

To address this comment, we have attached the **Supplementary Note 1** below:

Supplementary Note 1. EnergyPlus simulation of the light regulation and cooling-energy consumption of the cultivation model

S1.1 Model inputs and boundary conditions

A single-zone cultivation model (3.2 m high × 7 m wide × 7 m long) was developed in OpenStudio/EnergyPlus to quantify the influence of dynamic EC roof shading on (i) light regulation within the crop zone and (ii) cooling-energy demand under Singapore's

climate. Side walls were modelled using a conventional greenhouse film in both EC and Ref cases. Roof optical properties for the EC (colored/bleached) and Ref roofs, together with side-wall properties, are summarized in Supplementary Table 5. The cultivation region was represented as an effective crop zone (no explicit plant geometry), and no additional mechanical ventilation control was imposed. Infiltration/outdoor-air exchange was treated identically in the EC and Ref simulations; therefore, differences in cooling-energy demand arise from the roof optical properties and EC switching control rather than ventilation settings. Cooling-energy simulations employed a ZoneHVAC:IdealLoadsAirSystem with a constant indoor cooling set point of 24 °C. Simulations used a 10-minute time step, and outputs were reported at the time step resolution.

S1.2 Dynamic EC roof control in EnergyPlus (EMS)

The EC roof shading state was controlled through an Energy Management System (EMS) according to the outdoor incident solar radiation on the roof surface. The EC roof shading switched to the colored state when the incident solar radiation exceeded 530 W/m^2, switched to the bleached state when it fell below 380 W/m^2, and retained its previous state between these thresholds:

$$T_{EC}(t) = \begin{cases} T_{colored}, & E_{roof}(t) > E_{col} \\ T_{bleached}, & E_{roof}(t) < E_{ble} \\ T_{EC(t-1)}, & E_{ble} \le E_{roof}(t) \le E_{col} \end{cases} \quad \text{(S1)}$$

where $E_{roof}(t)$ represents the incident solar radiation on the roof surface in W/m^2, E_{col} =530 and E_{ble} = 380 W/m^2, and $T_{colored}$ and $T_{bleached}$ denote the transmittance of the EC roof shading in colored and bleached states, respectively. $T_{EC}(t)$ is not limited to transmittance but also applies to reflectance-related parameters. For the Ref shading case, the roof optical performance remained constant. The same EMS logic was applied in both the daylighting and cooling-energy simulations.

S1.3 Output 1: Light regulation within the cultivation zone

To characterize light regulation within the cultivation zone, illuminance sampling points were distributed vertically from 0.6 m to 3.0 m above the floor at 0.16-m intervals (N = 16; **Supplementary Table 8**). The point height is:

$$z_i = z_1 + \Delta z\,(i-1)\ ,\ i=1,\ldots,N \tag{S2}$$

EnergyPlus timestep daylighting calculations were used to obtain illuminance $I_i(t)$ (lux) at each point, which accounts for transmitted visible daylight and internal visible reflection inside the cultivation model. The zone-mean illuminance across the cultivation zone was calculated as:

$$\bar{I}(t) = \frac{1}{N}\sum_{i=1}^{N} I_i(t) \tag{S4}$$

where $\bar{I}(t)$ is the average illuminance within the cultivation zone at time t (lux). For comparison with crop-relevant metrics, illuminance was converted to PAR using a standard conversion factor,[1] and monthly mean PAR values were reported for EC and Ref cases.

S1.4 Output 2: Cooling-energy demand and energy-savings calculation

For the cooling-energy analysis, the transmitted shortwave solar radiation entering the cultivation zone was calculated as:

$$q_{rad}(t) = E_{roof}(t)A_{roof}T_{roof}(t) + \sum_{j=1}^{4} E_{wall,j}(t)A_{wall,j}T_{wall} \tag{S5}$$

where $q_{rad}(t)$ is the transmitted shortwave solar radiation entering the cultivation model at time t (W), $E_{roof}(t)$ and $E_{wall,j}(t)$ are the incident solar radiation on the roof and wall surfaces (W/m^2), A_{roof} and $A_{wall,j}$ are the corresponding surface areas (m^2), and $T_{roof}(t)$ and T_{wall} is the solar transmittance of the roof shading and side-walls, respectively.

The transmitted shortwave input $q_{rad}(t)$ in Eq. (S5) serves as the primary radiative input to the cultivation model. In addition, to account for effective radiative heat influence from surrounding surfaces (e.g., enclosure and ground), we introduced a supplementary radiative heat-input term.[2,3] This term was implemented in EnergyPlus as an EMS-controlled radiant internal gain and was applied consistently in both EC and Ref models to preserve comparability. We note that this auxiliary term is introduced as an equivalent

radiant load and does not modify the native EnergyPlus surface longwave-exchange algorithm, thereby avoiding double counting with the standard longwave radiation exchange solved internally by EnergyPlus.

In the present model, the supplementary radiative contribution was approximated as a fixed fraction of the total effective heat input:

$$q_{use}(t) = \frac{q_{rad}(t)}{1-f_{th}} \tag{S6}$$

$$q_{\mathrm{th}}(t) = f_{th} q_{\mathrm{use}}(t) \tag{S7}$$

where $q_{use}(t)$ is the total effective radiative energy input after accounting for transmitted shortwave solar radiation and supplementary radiative component (W), $q_{th}(t)$ is the thermal radiation component (W), and f_{th} is the thermal radiation fraction. The corresponding effective radiative-input parameter is listed in **Supplementary Table 8**.

The total absorbed indoor shortwave radiative gain was written as:

$$q_{sol,tot}(t) = \sum_s q_{sol,s}(t) \tag{S8}$$

where $q_{sol,s}(t)$ is the absorbed radiative gain at interior surface *s*. In EnergyPlus, transmitted solar radiation is distributed over the interior surfaces according to the cultivation model geometry, solar optical properties, and solar position, and the absorbed shortwave gain at the interior surface can be represented conceptually as:

$$q_{sol,s}(t) = \Psi_s(t) q_{use}(t) \tag{S9}$$

where $\Psi_s(t)$ is an effective surface-specific solar-distribution coefficient determined by the standard EnergyPlus solar-distribution routine. From a physical perspective, part of the effective radiative input absorbed by the crop canopy is ultimately partitioned into latent heat associated with evapotranspiration and sensible heat associated with crop-air heat exchange.[4,5] As crop sensible heat can be one possible pathway through which absorbed radiation is transferred to the indoor air, in the present study, this latent/sensible partition was therefore considered as a mechanistic framework for interpreting how canopy-absorbed radiation contributes to the model thermal regulation rather than as additional prescribed loads, to avoid double counting with thermal gains.

At the indoor surface level, the absorbed solar gains were resolved together with longwave radiation exchange, conduction through the roof shading, and convection with the indoor air through the standard surface heat balance, which can be written as:

$$C_s \frac{dT_s(t)}{dt} = q_{sol,s}(t) + q_{LW,s}(t) + q_{cond,s}(t) - q_{conv,s}(t) \qquad \text{(S11)}$$

where C_s is the thermal capacitance of interior surface *s*, $T_s(t)$ is the inside surface temperature, $q_{sol,s}(t)$ is the absorbed shortwave solar heat gain, $q_{LW,s}(t)$ is the net longwave radiative exchange, $q_{cond,s}(t)$ is the conductive heat transfer through the surface, and $q_{conv,s}(t)$ is the convective heat exchange between surface *s* and indoor air. Here, $q_{sol,s}(t)$ is determined from the effective radiative input together with the solar optical properties and geometry of the cultivation model. $q_{LW,s}(t)$ is determined by the infrared emissivity properties of the roof shading/side wall materials and the enclosure radiative exchange conditions. $q_{cond,s}(t)$ represents the conductive heat transfer through the roof shading and side wall materials, determined by the material thickness and conductivity. These terms were internally resolved by the standard EnergyPlus surface heat-balance routines and were not prescribed as independent input loads.

The convective term is:

$$q_{conv,s}(t) = h_{c,s}(t) A_s \left(T_s(t) - T_Z(t)\right) \qquad \text{(S12)}$$

where $h_{c,s}(t)$ is the inside convective heat-transfer coefficient, A_s is the surface area, and $T_z(t)$ is the zone air temperature.

The zone air temperature was then determined from the zone air heat balance:

$$C_z \frac{dT_z(t)}{dt} = \sum_s q_{conv,s}(t) + m_{inf}(t) c_p \left(T_{out}(t) - T_Z(t)\right) + m_{sys}(t) c_p (T_{sup}(t) - T_z(t)) + Q_{int.sens}(t) \qquad \text{(S13)}$$

where C_z is the thermal capacitance of the zone air, $m_{inf}(t)$ is the infiltration/outdoor-air exchange rate, $m_{sys}(t)$ is the HVAC supply-air mass flow rate, $T_{out}(t)$ is the outdoor air temperature, $T_{sup}(t)$ is the supply-air temperature, and $Q_{int,sens}(t)$ is the net sensible internal gain (including the EMS-controlled supplementary radiant internal gain described above). Under the ZoneHVAC:IdealLoadsAirSystem assumption, the cooling system provides the ideal sensible and latent removal required to maintain the zone air temperature at the prescribed set point. The instantaneous ideal cooling rate can be expressed as:

$$q_{cool}(t) = m_{sys}(t) c_p (T_z(t) - T_{sup}(t)) \qquad \text{(S14)}$$

for the case where $T_z(t)$ exceeds the cooling setpoint and cooling is required.

In the present study, cooling-energy was obtained directly from the EnergyPlus output variable “Zone Ideal Loads Supply Air Total Cooling Energy [J] (TimeStep)”, reported

at the 10-min timestep resolution over a full year using Singapore's typical meteorological data. Since the EnergyPlus output used here is a timestep cooling-energy quantity, the monthly cooling-energy was obtained by summing the timestep cooling-energy output within each month:

$$E_{cool,m} = \sum_{n \in m} E_{cool,n} \tag{S15}$$

and the annual cooling-energy was similarly obtained by summing all timestep cooling-energy values over the full-year:

$$E_{cool,year} = \sum_{n=1}^{N_{year}} E_{cool,n} \tag{S16}$$

The monthly cooling-energy saving achieved by the EC roof shading was calculated as:

$$\Delta E_m = E_{cool,m}^{ref} - E_{cool,m}^{EC} \tag{S17}$$

Similarly, the annual cooling-energy saving was calculated as:

$$\Delta E_{year} = E_{cool,year}^{ref} - E_{cool,year}^{EC} \tag{S18}$$

where E_{cool}^{ref} and E_{cool}^{EC} denote the cooling-energy demands of the reference roof shading and EC roof shading, respectively.

S1.5 Cooling-energy evaluation across global climate zones

To assess geographic applicability, the EnergyPlus cultivation model was simulated using typical meteorological year (TMY/IWEC/CWEC) weather files from 16 representative cities spanning climate zones 0A–6A, obtained from the official EnergyPlus weather database. For each city, the same model geometry, material properties, EMS switching logic, timestep (10 min), and HVAC control settings were retained, and only the weather file was changed (Supplementary Table 7).

Cooling energy for each city was obtained directly from the EnergyPlus output variable "Zone Ideal Loads Supply Air Total Cooling Energy [J] (TimeStep)", reported at the timestep resolution. For equatorial and tropical regions (climate zones 0A–1B), cooling-energy totals were evaluated over the full year. For cities within climate zones 2A–6A with distinct hot/cold seasons, the analysis focused on the hot seasons (June to August for cities in the Northern Hemisphere; December to February for cities in the Southern Hemisphere) to quantify cooling-energy savings under cooling-dominated conditions, consistent with the intended operating scope of the irradiance-adaptive EC shading.

The seasonal cooling energy was computed by summing timestep cooling-energy outputs over the selected analysis period P:

$$E_{cool,p} = \sum_{n \in p} E_{cool,n} \quad \text{(S19)}$$

where Ecool,n is the timestep cooling energy output and P denotes either (i) the full-year period (for zones 0A–1B) or (ii) the June–August period (for cities in the Northern Hemisphere within zones 2A–6A) or December to February period (for cities in the Southern Hemisphere within zones 2A–6A). The corresponding cooling-energy saving was calculated as:

$$\Delta E_p = E_{cool,p}^{ref} - E_{cool,p}^{EC} \quad \text{(S20)}$$

Note: For climates outside the tropics, we report cooling-energy results for the hot season to quantify cooling-dominated performance; year-round heating and cooling results are provided separately to illustrate potential cold-season heating penalties (Supplementary Fig. 12).

Comment #6. *The results of the work should be described in greater detail. For example, a bar chart is presented showing the results obtained from the simulations for different locations, but these results are not described exhaustively.*

Response: We thank the reviewer for this helpful suggestion. To directly address this concern, we have expanded the description of the simulation results associated with Fig. 5 to provide a more quantitative and comprehensive interpretation across different locations and climate zones. Specifically, for the bar chart (Fig. 5c), we report the numerical cooling-energy savings range across representative cities and highlight the highest- and lowest-saving cases, while clarifying the evaluation windows (year-round for climate zones 0A–1B and hot-season results for zones 2A–6A) consistent with the cooling-dominated operating scope:

Lines 317-333, Page 12-13:

To assess the geographical applicability of EC shading's cooling potential under hot climates, we extended the simulation to 16 representative global cities spanning climate zones 0A to 6A using location-specific weather data. For equatorial and tropical hot-climate zones (0A–1B; 0A/0B/1A/1B), which experience persistently intense solar irradiance and high ambient temperatures across much of the year,[62] cooling-energy savings were evaluated on a year-round basis. In these regions, EC shading yields the largest absolute savings compared with the Ref shading, reaching 46.91GJ in Port Hedland (1B), 39.34 GJ in Karachi (0B), 34.49 GJ in Singapore (0A) and 22.44 GJ in Hong Kong (1A) (Fig. 5c). For climate zones with distinct hot/cold seasons (2A-6A), values in Fig.5c correspond to the hot-season totals (June to August for Northern Hemisphere cities; December to February for Southern Hemisphere cities) to quantify cooling-energy saving performance. Under this seasonal evaluation, intermediate savings are obtained in warm-to-hot locations such as Lima (2B, 16.02 GJ) and Thessaloniki (3B, 14.99 GJ), whereas smaller savings occur in milder or cooler climates with lower cooling demand, including Nairobi (3C, 4.19 GJ) and Stockholm (6A, 4.38 GJ). Overall, the geographic variation in cooling-energy savings is consistent with differences in cooling loads driven by ambient temperature and solar heat-gain potential: locations with hotter conditions and higher irradiance exhibit larger absolute cooling-energy savings under EC shading. Cooling-energy savings remain consistently positive across all evaluated locations within the scope of the hot-season analysis.

For the global map (Fig. 5d), we added a concise explanation of the climate-zone-based extrapolation method and described the spatial patterns, identifying high-savings hot-spot regions and explaining their linkage to cooling demand and solar heat-gain potential. Then, we strengthened the discussion of the implications for future smart-farming deployment (Fig. 5e), emphasizing the consistent benefits across climate zones and the scalability of the proposed irradiance-adaptive EC shading platform for urban farming infrastructures:

Lines 334-346, Page 13

> To visualize the broader geographic implications, the cooling-energy savings associated with each representative city were extrapolated to all global locations sharing the same climate-zone classification (Fig. 5d). This zone-based extrapolation converts the discrete results in Fig. 5c into continuous geographic patterns and highlights where EC shading is expected to deliver the greatest cooling-energy benefits. Significant cooling-energy savings "hot spots" emerge across large parts of the tropics and subtropics, as well as hot arid and semi-arid regions, most notably the Middle East and North Africa, consistent with their high solar heat-gain potential and substantial cooling demand. In contrast, temperate and cold regions exhibit weaker savings, reflecting lower cooling-energy demand. Importantly, the consistent cooling-energy reductions observed across all simulated zones indicate that the irradiance-adaptive EC shading can be integrated into a wide range of greenhouse-type or farm cultivation infrastructures, supporting energy-efficient, climate-adaptive crop production. This scalability and geographic robustness highlight EC shading as a promising platform for futuristic smart farming, enabling responsive microclimate control on existing greenhouse cultivation structures (Fig. 5e).

To address this comment, we have made these revisions in **Global modelling of light regulation and cooling-energy savings with EC shading** part to provide a more exhaustive description of the simulation results, as requested by the reviewer.

Reviewer #2 (Remarks to the Author):

The authors report about an electrochromic system able to provide controlled shading to greenhouses, in order to provide internal adaptive photo and thermal regulation. The results, tested for lettuce growing, look promising for a mainly unexplored application of electrochromic technology. These findings can be interesting and innovative in the field, but some concerns arise in order to consider the manuscript as a sound reference. Let me comment on that.

We thank the referee for this constructive suggestion and appreciate the overall positive assessment that our results are promising for a largely unexplored application of electrochromic technology in adaptive photo-thermal regulation for urban farming. Listed below are the reviewer's comments and our responses. We hope that we have addressed all of the reviewer's comments.

Comment #1. *There is a critical aspect that should be considered and commented about the capabilities of these devices to modulate solar spectrum. The problem is that, presently, they cannot modulate UV nor NIR. This is because glass and FTO, main components of the device, act as passive filters in those ranges. This limits the functionality of the devices to Visible range. The authors should clearly state these limitations and discuss them.*

Response: We thank the reviewer for this important and constructive comment. To address this point directly: we agree that the UV and NIR blocking behavior is largely governed by the optical filtering of the FTO-glass substrates in the ECD with the structure of FTO-glass/electrolyte/WO_3/FTO-glass, and that the primary switchable functionality of the device lies in the visible (PAR) range. We have revised the manuscript to explicitly clarify this limitation, while quantitatively distinguishing between passive filtering and active electrochromic modulation.

First, we quantified passive filtering of a bare FTO–glass substrate using AM1.5G-weighted, band-integrated transmittance metrics, calculated as follows:

$$T_{UV} = \int_{280}^{400} T(\lambda)\phi_{sol}(\lambda)d\lambda \,/\, \int_{280}^{400} \phi_{sol}(\lambda)d\lambda \quad (1)$$

$$T_{VIS} = \int_{400}^{780} T(\lambda)\phi_{VIS}(\lambda)d\lambda \,/\, \int_{400}^{780} \phi_{VIS}(\lambda)d\lambda \quad (2)$$

$$T_{NIR} = \int_{780}^{2500} T(\lambda)\phi_{sol}(\lambda)d\lambda \,/\, \int_{780}^{2500} \phi_{sol}(\lambda)d\lambda \quad (3)$$

where T(λ) represents the transmittance at wavelength λ, $\phi_{vis}(\lambda)$ and $\phi_{sol}(\lambda)$ are the standard efficiency functions for the photopic vision and the solar irradiance spectrum for an air mass of 1.5.

The resulting values (**Fig. R3, Revised Supplementary Fig. 1**) are T_{uv}=48.1%, T_{vis}=76.5%, T_{NIR}=47.9%, confirming that FTO-glass is a non-negligible passive attenuator in both UV and NIR regions. As the ECD architecture consists of two FTO–glass panes (FTO-glass/WO_3/electrolyte/FTO-glass), the overall UV/NIR attenuation is further enhanced through a multiplicative stacking effect.[10]

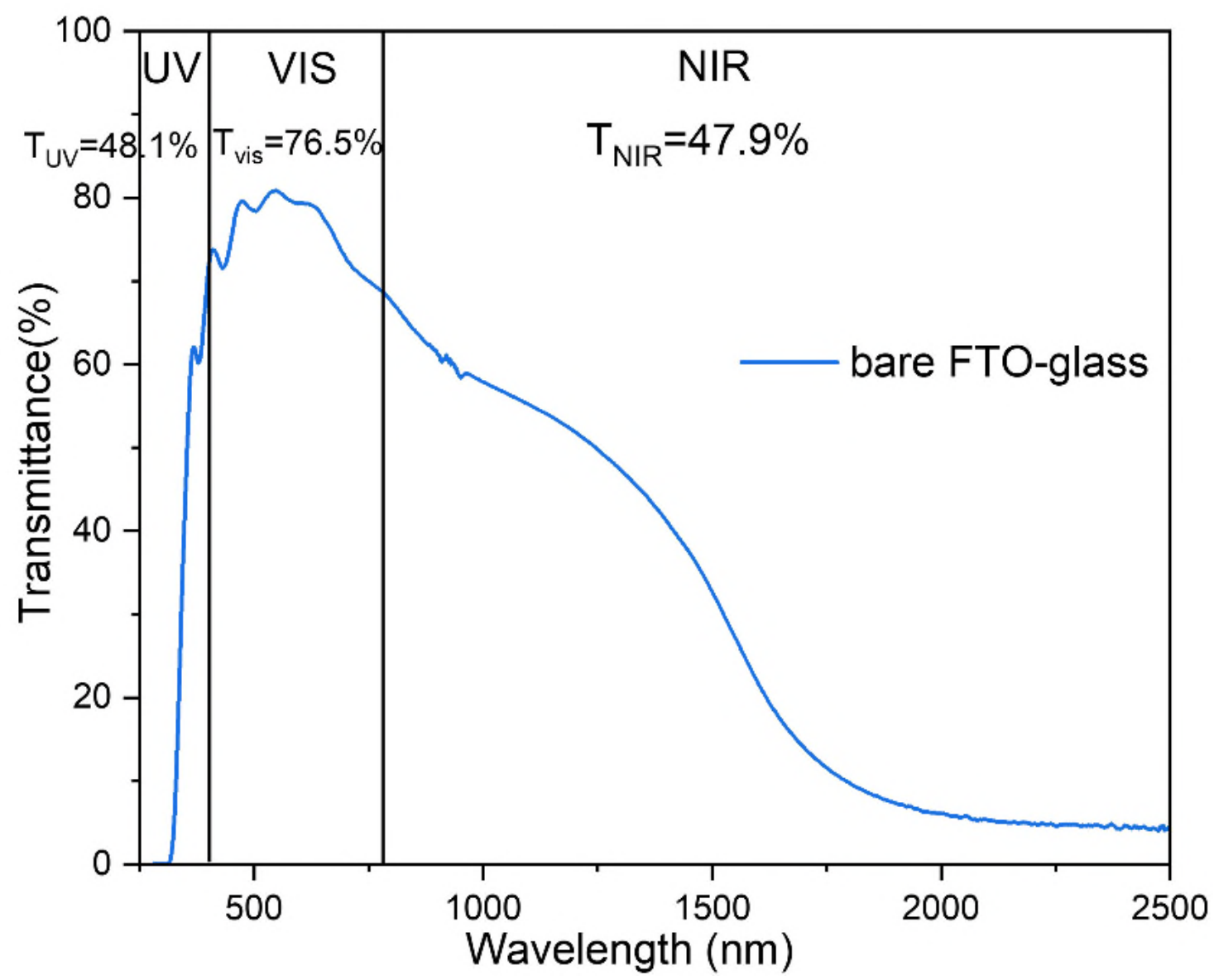

Fig. R3 (Revised Supplementary Fig. 1). Bare FTO-glass spectral transmittance and band-integrated transmittance value

Building on this, we report the band-integrated UV/NIR suppression of the complete device in both optical states (Supplementary Table 1):

Table. R1 (Revised Supplementary Table 1). Band-integrated UV/VIS/NIR transmittance and blocking (EN 410-type metrics)

State	T_{UV}(280-400 nm)	UV blocking	T_{VIS}(400-780 nm)	VIS blocking	T_{NIR} (780-2500 nm)	NIR blocking
Bleached	18.2%	81.8%	57.6%	42.4%	22.4%	77.6%
Colored	16.3%	83.7%	24.0%	76%	3.7%	96.3%

The ECD exhibits strong UV suppression in both optical states, blocking 83.7% (colored) and 81.8% (bleached) of UV radiation. This behavior is governed by the passive filtering of the FTO–glass substrates and is therefore essentially state-independent. For the NIR region, the ECD blocks 96.3% (colored) and 77.6% (bleached) of the integrated NIR radiation. While NIR attenuation is substantial in both states and primarily determined by the FTO-glass baseline, with an additional moderate modulation (~18.7%) arising from the electrochromic layer.

Therefore, we agree with the reviewer that the primary active functionality of the present ECD lies in the visible region, where the device provides pronounced and dynamically tunable modulation of PAR. In contrast, UV attenuation is not actively modulated, and NIR modulation is relatively limited compared with the visible range.

To address this comment, we have

1. **revised the manuscript text to accurately describe the spectral-modulation capability**.

We avoid overstating "solar-spectrum modulation" and to clarify that the present platform provides irradiance-adaptive visible/PAR regulation, while UV attenuation is primarily governed by the passive filtering of the FTO-glass substrates, and NIR attenuation is dominated by substrate filtering with additional moderate electrochromic modulation. We added the measured spectral transmittance and AM1.5G-weighted band-integrated Transmittance of bare FTO-glass substrate as **Supplementary Fig.1** to support this point:

Lines 102-111, Page 4

> This state transition provides coordinated photothermal regulation by reducing radiative heat gain through passive UV suppression and NIR attenuation, while tuning both the intensity and spectral composition of PAR (Fig. 1c, d). As shown in Fig.1d (centre and right), the ECD in both colored and bleached states strongly suppresses UV transmission and maintains low NIR transmittance, while providing switchable visible light transmission. Mechanistically, UV suppression is dominated by the passive filtering of the FTO-glass substrates (Supplementary Fig. 1), whereas NIR attenuation is also substantial in both states and primarily governed by the FTO-glass baseline, with additional moderate electrochromic modulation. These effects are quantified for the complete device using EN 410-type band-integrated metrics (Supplementary Table 1):

the ECD blocks 83.7%/81.8% (colored/bleached) of UV radiation (280–400 nm), and attenuates 96.3%/77.6% (colored/bleached) of NIR radiation (780–2500 nm), respectively.

Lines 360-364, Pages 14-15

Building upon this device-level robustness, the integrated EC shading system autonomously switches between bleached and colored states in response to real-time solar irradiance, enabling coordinated photothermal regulation by dynamically tuning PAR transmission while maintaining suppressed UV transmission and substrate-dominated NIR attenuation to reduce radiative heat gain.

2. **added a limitation statement in the Discussion**.

We clarify that the relatively low NIR transmission in both colored/bleached states, arising from the FTO-glass substrate baseline, constrains applicability to cooling-dominated hot-climate operation, and that extending to all-season operation requires alternative NIR-transparent conductive electrodes in **future work**:

Lines 381-385, Page 15

Notably, in the present architecture, UV attenuation is primarily determined by the passive filtering of the FTO-glass substrates (Supplementary Fig.1), while NIR transmission remains relatively low in both states due to the substrate baseline, with only limited electrochromic modulation. This results in persistently reduced NIR transmission in both bleached/colored states.

Lines 396-399, Page 16

Second, extending toward truly all-season operation will require season-aware control and device designs that retain high NIR transmittance in the bleached state for passive warming in cold seasons, which will place stricter demands on device architecture and scalable NIR-transparent conductive materials.

References:

10. https://www.newport.com/g/neutral-density-filter-selection-guide

Comment #2. *This relates with a second concern, and is about the extension to which this technology can be applied for different climate conditions. The authors claim that this is an "all-weather" solution, which is clearly arguable. The proposed architecture is mainly useful for hot climates, but probably counter-productive in warm or cold climates. Blocking NIR range in cold seasons could be damaging to the crop, as this radiation is needed to heat the inner space of the greenhouse. In the worst case, additional heating will be needed, with additional energy costs. The authors should not claim that this is an "all-weather" solution.*

Response: We sincerely thank the reviewer for raising this important point. To address this point directly: we agree that the present electrochromic shading system is not universally applicable across all climates and seasons, and is primarily suited for hot climates and cooling-dominated conditions. In response, we have **(i) clarified the intended scope and corrected the terminology ("all-weather"), (ii) discussed the limitations associated with NIR blocking in cold conditions, and (iii) outlined future directions toward season-adaptive and all-season-capable designs.**

(i) Clarification of "All-weather" terminology and application scope

We acknowledge that the term "all-weather" may be misleading and could be interpreted as implying applicability across all climates and seasons, including heating-dominated environments. This was not the intended meaning. In the original manuscript, "all-weather" referred specifically to the system's ability to operate robustly under fluctuating outdoor irradiance upon different sky/weather conditions (e.g., clear, cloudy, and overcast days), rather than universal climate applicability.

To address this, we have revised the manuscript to remove/replace "all-weather" and adopt more accurate terminology (e.g., irradiance-adaptive), while stating that the proposed EC shading is primarily intended for hot climates and cooling-dominated, heat-intensive regions where overheating and heat stress constrain crop productivity. Under these conditions, the EC shading provides coordinated photothermal regulation by suppressing heat-inducing radiation and maintaining crop-relevant PAR within an optimal range, thereby helping to mitigate high-temperature heat stress and improve growth conditions.

(ii) Limitation in cold or heating-dominated conditions

We fully agree with the reviewer that in cold seasons or heating-dominated climates, strong attenuation of near-infrared (NIR) radiation may reduce beneficial passive solar heating and potentially increase greenhouse heating demand.

In the present architecture, UV and NIR attenuation are largely governed by the passive filtering of the FTO-glass substrates, resulting in persistently reduced NIR transmission even in the bleached state. While this is beneficial for suppressing radiative heat gain in hot climates, it becomes a limitation in cold conditions where solar heat gain is desirable. We have therefore stated in the revised manuscript that performance under heating-dominated climates is beyond the intended scope of this work.

(iii) Future directions toward all-season applicability

We acknowledge that extending the system toward truly all-season operation is an important future research direction. Achieving this requires EC systems that can preserve high NIR transmittance in the bleached state to provide a warming function in cold seasons, while still remaining effective shading in hot conditions.

We further highlight that such functionality will place more stringent requirements on the device architecture and the selection/engineering of NIR-transparent conductive materials.

To address this comment, we have:

1. Revised terminology and scope: We replaced the term "all-weather" throughout the manuscript with "irradiance-adaptive" to accurately describe robust switching under fluctuating outdoor solar irradiance (e.g., clear/cloudy/overcast conditions) for hot climates. We also scope the system to hot climates and cooling-dominated, heat-stress-intensive urban farming scenarios (some examples are shown below):

Lines 1-2, Page 1 (Title)

> **Scalable irradiance-adaptive electrochromic shading for photothermal regulation in controlled-environment agriculture**

Lines 13-14, Page 1

> Here, we report a scalable, irradiance-adaptive EC shading system in hot climates that enables photothermal regulation under fluctuating outdoor conditions.

Lines 356-357, Page 14

Overall, we developed a high-performance ECD that functions as the core building block of a scalable, irradiance-adaptive EC shading system for controlled-environment agriculture under hot climates.

2. Added limitation and future directions for cold season conditions in **Discussion** part:

Lines 381-399, Page 15

Notably, in the present architecture, the UV/NIR attenuation is largely set by the FTO-glass substrates (passive filtering; Supplementary Fig.1), while NIR transmission remains relatively low in both states due to the substrate baseline, with only limited electrochromic modulation. This results in persistently reduced NIR transmission in both bleached/colored states. However, when extending the analysis to year-round operation in climate zones with distinct heating seasons (2A–6A), the same irradiance-triggered control can incur a heating penalty due to NIR attenuation, which reduces passive solar warming and may increase heating energy demand. As summarized in Supplementary Fig. 12, this heating penalty can substantially reduce and even negate (in cold climate zones) the net annual energy benefit. These results reinforce that the present system is primarily suited for cooling-dominated operation (hot climates and hot seasons), and motivate season-aware control and device designs as key future directions.

Lines 396-399, Page 16

Second, extending toward truly all-season operation will require season-aware control and device designs that retain high NIR transmittance in the bleached state for passive warming in cold seasons, which will place stricter demands on device architecture and scalable NIR-transparent conductive materials.

Comment #3. *Regarding the electrochromic material chosen, I would say WO_3 is not probably the best option. A tailored material that does not absorb red or blue ranges could be beneficial (the authors point to this direction too, lines 332-334. In any case I don't think this is critical at this moment and this material can be used as a proof of concept.*

Response: We thank the reviewer for this constructive suggestion. We agree that WO_3 is not necessarily the optimal electrochromic material for all crop-specific spectral needs, and that developing tailored electrochromic films that selectively modulate targeted wavelength bands while preserving others (e.g., red or blue, depending on crop physiology) could further improve performance.

In this work, WO_3 was selected as a proof-of-concept electrochromic layer because it is well-established, scalable, and offers robust large-area device performance (optical contrast, switching reversibility, optical memory, and outdoor durability), which was essential for demonstrating field-deployable, irradiance-adaptive EC shading.

To address this comment, we have revised the Discussion to acknowledge that crop-specific electrochromic materials and spectral-selective designs represent an important future direction, consistent with our statement on tailoring electrochromic films for crop-specific spectral modulation:

Lines 399-403, Page 16

> Third, tailoring electrochromic films for crop-specific spectral modulation would enable more precise control of light intensity and wavelength ratios to match diverse photosynthetic requirements, for example, by designing band-selective electrochromic responses that minimize absorption in crop-beneficial blue/red regions while tuning green transmission and overall PAR to better align spectral composition with crop physiological needs.

Comment #4. *Climate modelling. As I mentioned before, the proposed configuration won't be practical for warm or cold climates. Indeed, when performing the analysis of energy savings, just the hot season is taking into consideration for those climates. This is not a valid analysis: an all-year analysis should be performed in these climates. Energy savings in hot season could be accompanied by the need of heating energy costs during cold season (due to NIR blocking) and consequently result in net savings below Zero.*

Response: We thank the reviewer for this thorough and important comment regarding the climate-modelling framework and the need for a full-year energy assessment. We fully agree that if a system is intended for universal, all-climate deployment, a complete annual evaluation including both cooling and heating demands is essential. However, we clarify that our work was never intended to claim universal all-season applicability. Instead, the proposed irradiance-adaptive EC shading is designed for cooling-dominated greenhouse environments, where the primary objective is to mitigate high-temperature heat stress in urban farming by maintaining crop-relevant PAR within an optimal range under hot climates or hot seasons.

In response to this comment, we have **(i) clarified the intended application scope and refined the climate-modelling methodology accordingly, and (ii) additionally performed year-round simulations to explicitly quantify heating penalties in cold seasons and transitional climates.**

(i) Clarification of application scope and Refinement of climate-modelling methodology

We agree with the reviewer that the previous presentation could be misinterpreted as implying broader all-climate energy neutrality. To avoid this, we have clarified that the system is targeted at hot climates and cooling-dominated periods, where excessive solar heat gain is the dominant constraint for greenhouse operation. In cold seasons (heating-dominated periods), continued NIR attenuation can reduce passive solar warming and may increase heating demand; therefore, applying the same irradiance-only control logic for annual net-energy evaluation in cold climates can be counterproductive.

Accordingly, the energy analysis in the main manuscript is framed within this intended scope: cooling-energy savings are evaluated as the primary metric for system performance under heat-stress conditions. For climates zones with consistently high temperatures (equatorial/tropical zones) (0A–1B), a full-year cooling analysis is appropriate; whereas for climates zones with distinct

seasonal variability (2A–6A), the relevant performance metric is the hot-season cooling demand reduction.

(ii) Additional year-round simulations and quantification of heating penalty

To directly address the reviewer's concern, we have additionally performed year-round simulations including both heating and cooling-energy for representative climates (2A-6A) with distinct hot/cold seasons, using a heating setpoint of 20 °C and a cooling setpoint of 24 °C consistent with lettuce temperature requirements.[11] This year-round analysis confirms the reviewer's point: As shown in **Fig. R6 (Revised Supplementary Fig.12)**, in those climate zones with cold seasons/heating-dominated conditions, the EC shading can incur a heating penalty (negative heating energy saving relative to reference shading), which can substantially reduce or even negate the annual net energy saving (shown as the purple part). For example, in Stockholm (climate zone 6A), where heating demand dominates (mostly cold seasons), annual heating energy demand is higher under EC shading than under Ref shading, leading to a negative net annual energy saving. Similarly, in transitional climates (e.g., Vancouver 4C), EC shading provides cooling savings during hot periods, but the accompanying heating penalty during cold periods reduces the overall net annual energy saving. We include these year-round results (**Supplementary Fig. 12**) as supplementary evidence to transparently confirm the scope of applicability of the present irradiance-triggered EC shading strategy for hot climates and to acknowledge its limitation for all-season operation.

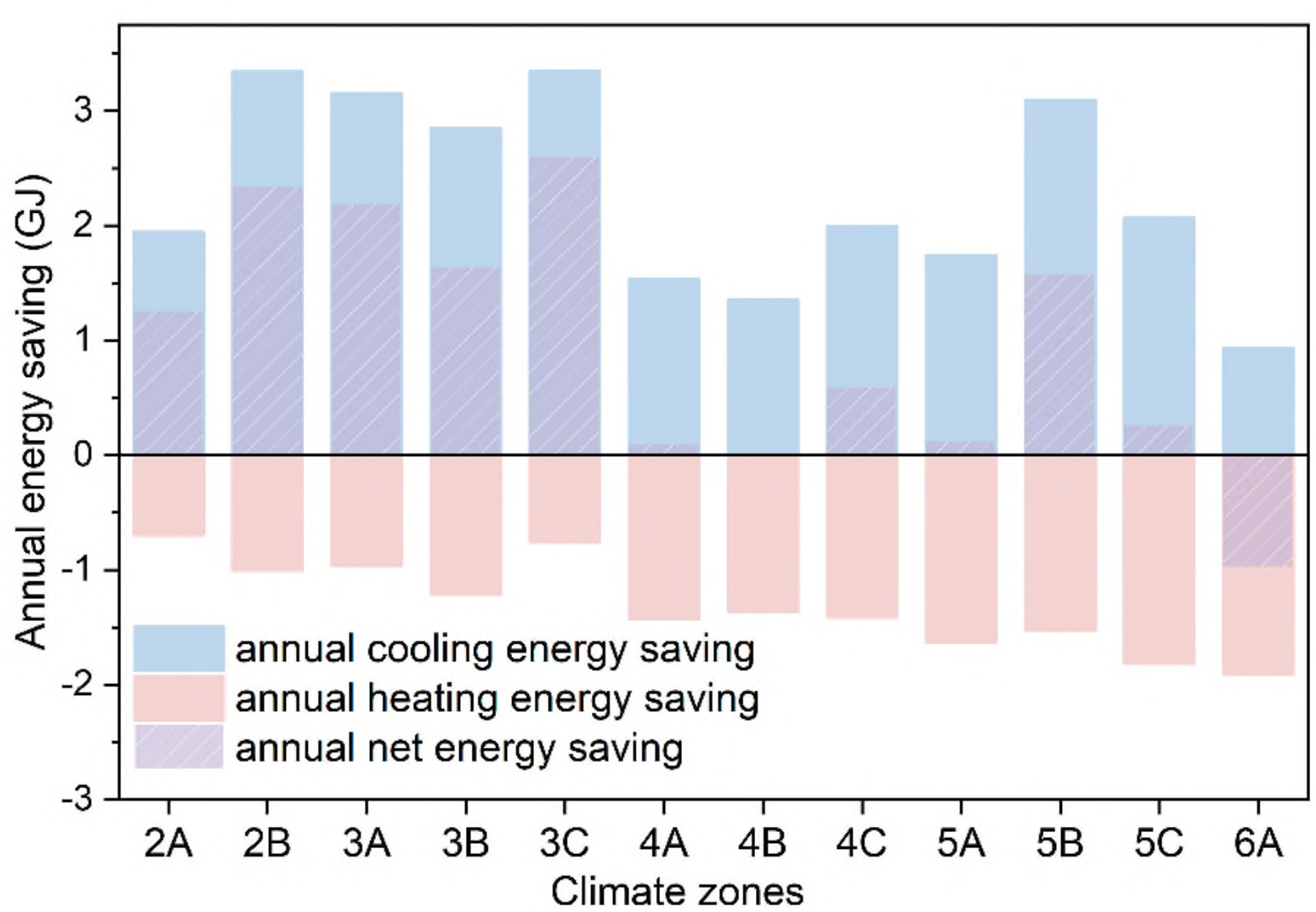

Fig. R6 (Revised Supplementary Fig. 12). The annual cooling/heating/net energy saving of EC shading relative to Ref shading across climate zones (2A-6A), based on year-round EnergyPlus simulations (heating setpoint: 20 °C; cooling setpoint: 24 °C).

Together, these revisions ensure that our claims are aligned with the cooling-dominated application domain of the proposed system, while acknowledging and quantifying the potential heating penalty outside this scope.

To address this comment, we have

1. Clarified the application scope: We revised the scope of the irradiance-adaptive EC shading system to hot climates and cooling-dominated, heat-stress-intensive urban farming, where mitigating overheating and maintaining crop-optimal PAR are the primary objectives throughout the manuscript (some examples are shown below):

Lines 13-14, Page 1

> Here, we report a scalable, irradiance-adaptive EC shading system in hot climates that enables photothermal regulation under fluctuating outdoor conditions.

Lines 356-357, Page 14

> Overall, we developed a high-performance ECD that functions as the core building block of a scalable, irradiance-adaptive EC shading system for controlled-environment agriculture under hot climates.

.

2. Refined and validated the climate-modelling reporting framework. We clarified that the global EnergyPlus analysis (climate zones 0A–6A) reports cooling-energy savings under hot climates: year-round for equatorial/tropical zones (0A–1B) and hot-season performance for zones with distinct hot/cold seasons (2A–6A):

Lines 317-321, Page 12

> To assess the geographical applicability of EC shading's cooling potential under hot climates, we extended the simulation to 16 representative global cities spanning climate zones 0A to 6A using location-specific weather data. For equatorial and tropical hot-climate zones (0A–1B; 0A/0B/1A/1B), which experience persistently intense solar irradiance and high ambient temperatures across much of the year,[62] cooling-energy savings were evaluated on a year-round basis.

3. Added limitation and future directions for cold season conditions:

Lines 380-388, Page 15

Notably, in the present architecture, UV attenuation is primarily determined by the passive filtering of the FTO-glass substrates (Supplementary Fig.1), while NIR transmission remains relatively low in both states due to the substrate baseline, with only limited electrochromic modulation. This results in persistently reduced NIR transmission in both bleached/colored states. However, when extending the analysis to year-round operation in climate zones with distinct heating seasons (2A–6A), the same irradiance-triggered control can incur a heating penalty due to NIR attenuation, which reduces passive solar warming and may increase heating energy demand. As summarized in Supplementary Fig. 12, this heating penalty can substantially reduce and even negate (in cold climate zones) the net annual energy benefit. These results reinforce that the present system is primarily suited for cooling-dominated operation (hot climates and hot seasons), and motivate season-aware control and device designs as key future directions.

Lines 396-399, Page 16

Second, extending toward truly all-season operation will require season-aware control and device designs that retain high NIR transmittance in the bleached state for passive warming in cold seasons, which will place stricter demands on device architecture and scalable NIR-transparent conductive materials.

References:

11. Caplow, T. & Nelkin, J. Building-integrated greenhouse systems for low energy cooling. in vol. 1 172–176 (2007).

In view of these concerns, I recommend major revision of the manuscript.

Additional comments or corrections:

Comment #5. *Less critical, but please also consider it: Personally, I don't understand the use of "urban farming" along the text. The solution is clearly applicable to greenhouses and from my point of view this is the correct term to use.*

Response: We thank the Reviewer for this thoughtful and helpful comment. We agree that greenhouse systems represent an important and relevant application scenario for the proposed electrochromic shading technology.

Our original use of the terms "urban agriculture" and "urban farming" was intended to place this work within a broader context of localized food production in space-constrained urban environments. However, we acknowledge that these terms may reduce clarity when referring to the specific system investigated in this study.

In the literature, a greenhouse is broadly defined as a cultivation structure covered with transparent or semi-transparent materials that enables solar radiation transmission while enabling a certain degree of microclimate modification for plant growth.[12,13]

However, the experimental platform in this work consisting of an EC shading roof combined with partial side coverage using PE films does not constitute a fully enclosed or actively climate-controlled greenhouse system.

Therefore, to avoid potential ambiguity, we consider it more appropriate to describe the specific system as "**an outdoor or semi-covered cultivation system with electrochromic shading**." At the same time, we retain the term "**greenhouse cultivation**" when referring to the broader application context in which this technology can be applied.

To address this comment, we have revised the manuscript to improve terminological clarity and consistency:

1) We use "**greenhouse cultivation**" when referring to the broader application context of the EC shading technology.

2) We describe the specific experimental system more precisely as "**an outdoor or semi-covered cultivation system with electrochromic shading**", to reflect both the experimental platform, field deployment, and system-level operation in this work.

We appreciate the Reviewer's suggestion, which has improved the precision and clarity of the manuscript (examples are shown below):

Lines 34-35, Page 2

In response, controlled-environment agriculture, particularly greenhouse-type cultivation, has emerged as a promising strategy for localized and resource-efficient food production.[5,6] However, maintaining crop productivity in outdoor or semi-covered cultivation systems remains challenging, particularly in hot climates

Lines 39-40, Page 2

In subtropical and tropical climates, active cooling technologies such as fans, pumps, and air conditioning systems are widely employed to regulate temperature in greenhouse cultivation systems.

Lines 341-346, Page 13

Importantly, the consistent cooling-energy reductions observed across all simulated zones indicate that the irradiance-adaptive EC shading can be integrated into a wide range of greenhouse-type or farm cultivation infrastructures, supporting energy-efficient, climate-adaptive crop production. This scalability and geographic robustness highlight EC shading as a promising platform for futuristic smart farming, enabling responsive microclimate control on existing greenhouse cultivation structures (Fig. 5e).

Lines 356-357, Page 14

Overall, we developed a high-performance ECD that functions as the core building block of a scalable, irradiance-adaptive EC shading system for outdoor or semi-covered cultivation systems under hot climates.

Lines 412, Page 16

EC shading provides a viable pathway toward climate-resilient outdoor farm or greenhouse-type cultivation system.

References

12. von Elsner, B. et al. Review of Structural and Functional Characteristics of Greenhouses in European Union Countries: Part I, Design Requirements. Journal of Agricultural Engineering Research ***75***, 1–16 (2000)

13.FAO. Good Agricultural Practices for Greenhouse Vegetable Crops. Principles for Mediterranean Climate Areas. (Unspecified, 2013).

Comment #6. *Line 65: please correct this sentence "…ensuring reliable operations under realistic conditions…". Be aware that assuring a two-months stability period in a device does not guarantee reliable operation under realistic conditions. Realistic conditions in agricultural greenhouses would probably require several years of operation.*

Response: We thank the Reviewer for this important comment. We agree that a two-month outdoor exposure test is not sufficient to support statements implying reliable operation under realistic long-term agricultural greenhouse conditions. The original wording was therefore not sufficiently precise.

In response, we have revised this sentence and other similar expressions throughout the manuscript to avoid overstatement. Expressions such as "realistic outdoor conditions" have been replaced by more accurate descriptions that are strictly limited to the experimental evidence presented in this work, such as "retained electrochromic functionality over two-month outdoor tropical exposure".

To address this comment, we have revised the sentence below:

Lines 75-77, Page 3

> The platform is enabled by robust large-area EC devices with large optical contrast, fast switching, excellent optical memory, and maintains stable electrochromic performance over a two-month outdoor exposure test under tropical conditions.

Lines 179-180, Page 7

> The reduced contrast mainly arises from a lowered bleached-state transmittance, consistent with device aging under prolonged sunlight exposure, while switching kinetics remain comparable (52.1 s for coloring and 41.5 s for bleaching), indicating retained electrochromic functionality after two months of tropical outdoor exposure.

Lines 357-360, Page 14

> The 30 × 30 cm^2 ECD demonstrated a large optical contrast (51% at 633 nm), fast switching speeds, excellent optical memory effect, and maintained stable electrochromic performance over a two-month outdoor exposure test under tropical conditions.

Comment #7. *Lines 67, 68, 94: please avoid stating that the device modulates solar spectrum, specially in the UV or NIR ranges. It only modulates visible range, and just block the rest.*

Response: We thank the reviewer for this comment and agree that the original wording may overstate the spectral modulation capability. To clarify this point more precisely, we have revised the statements at Lines 67, 68, and 94 to explicitly distinguish between passive filtering and active electrochromic modulation across different spectral regions.

In the revised manuscript, we clarify that the electrochromic switching primarily enables active modulation in the visible (PAR) range, which constitutes the main functional component of the device. In contrast, UV attenuation is dominated by passive filtering from the FTO–glass substrates and is essentially state-independent, while NIR attenuation is also largely governed by the substrate baseline, with only limited additional modulation arising from the electrochromic layer. Accordingly, we have avoided statements implying full-spectrum modulation and instead adopted a more accurate description: the device provides irradiance-adaptive visible/PAR regulation, while UV is passively blocked and NIR is predominantly suppressed by the device structure of FTO-glass substrates with modest electrochromic contribution. Supporting quantitative analysis is provided in Table. R1 (Revised Supplementary Table 1).

Table. R1 (Revised Supplementary Table 1). Band-integrated UV/VIS/NIR transmittance and blocking (EN 410-type metrics)

State	T_{UV}(280-400 nm)	UV blocking	T_{VIS}(400-780 nm)	VIS blocking	T_{NIR} (780-2500 nm)	NIR blocking
Bleached	18.2%	81.8%	57.6%	42.4%	22.4%	77.6%
Colored	16.3%	83.7%	24.0%	76%	3.7%	96.3%

To address this comment, the statements at Lines 67,68 and 94 (Line 79,80,102 in the updated manuscript) are rewritten as:

Lines 79-82, Page 3

> This enables irradiance-adaptive regulation of visible (PAR) transmission under fluctuating outdoor conditions, maintaining PAR within optimal photosynthetic ranges (Fig. 1a), while suppressing excess radiative heat through passive UV suppression and substrate-dominated NIR attenuation.

Lines 102-104, Page 4

This state transition provides coordinated photothermal regulation by reducing radiative heat gain through passive UV suppression and NIR attenuation, while tuning both the intensity and spectral composition of PAR (Fig. 1c, d).

Comment #8. *Electrochromic device design: In the proposed architecture, FTO is used as simultaneously as counter electrode and substrate. No ion-storage material is used. In the past, these configurations have been reported to result in irreversible degradation of FTO (or ITO) visible through a browning of the substrate. The authors should comment on these aspects related to their own devices.*

Response: We sincerely thank the reviewer for this valuable comment. In response, we have **(i) clarified the degradation mechanism associated with the bare FTO configuration, including its relation to irreversible ion intercalation, (ii) discussed the architectural limitation and rationale for the present design, and (iii) provided supporting control-experiment evidence to validate the reviewer's concern and our interpretation.**

(i) Degradation behaviour and irreversible intercalation at the FTO side.

In the revised manuscript, we added the comparison of the initial and post-cycling transmittance–time (T–t) responses (**Fig. R1b, Revised Supplementary Fig. 4**) to show that the main degradation of the ECD is associated with a reduction in bleached-state transmittance after repeated cycling, whereas the colored-state transmittance changes less markedly. This behavior is consistent with limited charge reversibility and irreversible ion intercalation at the bare FTO counter side during repeated bleaching/coloration processes, in line with previous reports on bare FTO/ITO-based counter-electrode configurations.[2,3]

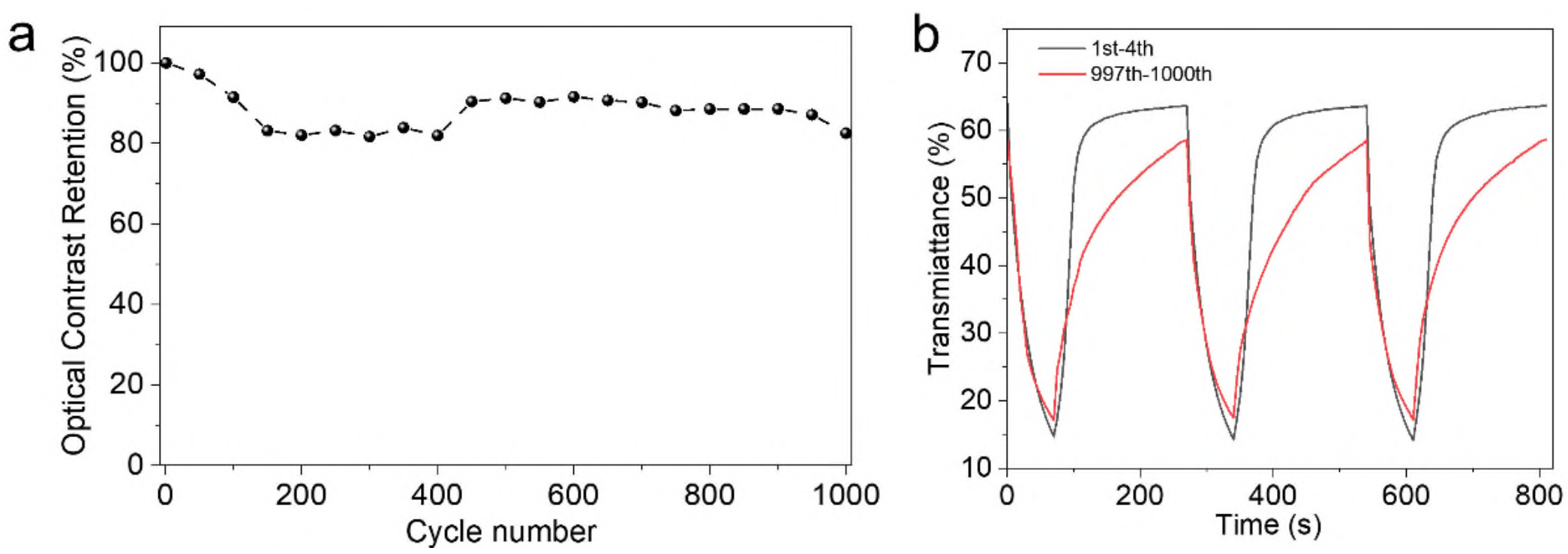

Fig. R1 (Revised Supplementary Fig. 4). **a,** Cycling performance of the ECD. **b,** the transmittance–time response during initial cycles and after 1000 cycles of the 30 × 30 cm^2 ECD

While visible substrate browning was not independently quantified in this study, the observed preferential loss in bleached-state transparency provides indirect but consistent evidence of the degradation trend expected from irreversible ion accumulation/intercalation at the bare FTO counter side

(ii) Architectural limitation and design rationale.

We acknowledge that, in the absence of a dedicated ion-storage layer, the bare FTO side serves as the counter side for charge compensation. Although this simplified configuration can support electrochromic switching, its charge-balance capability is intrinsically limited compared with that of a purpose-designed counter electrode, thereby limiting long-term stability.

From an application-oriented perspective, however, the primary objective of this work was to establish a scalable, outdoor-deployable EC shading platform for greenhouse cultivation. Under this context, the present large-area device already achieves the key performance metrics required for proof-of-concept demonstration, including 51% optical contrast (633 nm), long optical memory (>21600 s), 82.5% retention after 1000 cycles, and stable outdoor operation over two months under tropical conditions. Therefore, the current architecture was selected as a practical compromise between scalability, functionality, and field operability.

(iii) Supporting control experiment with ion-storage electrode.

To further examine the reviewer's concern on architectural limitation, we conducted a supporting control experiment using the same 30 × 30 cm^2 printed WO_3 electrode and the same electrolyte, but with an added currently under-development large-area PEDOT:PSS-based composite ion-storage electrode. Under these otherwise comparable conditions, the ion-storage electrode-based device achieved a similar optical contrast (50.0% at 633 nm) while operating at substantially reduced voltages (-2.0 V for 60 s / 0.1 V for 100 s, compared with -3.5 V for 70 s / 2.0 V for 200 s for the bare-FTO architecture), and exhibited improved cycling stability (91% optical-contrast retention after 1000 cycles, compared with 82.5% retention after 1000 cycles for the bare-FTO architecture) (**Fig. R2**).

These results directly support the reviewer's point and confirm that the absence of a dedicated ion-storage/counter-electrode layer is a key factor limiting charge balance and long-term stability in the present device architecture. As this architecture is still under active optimization for other performance metrics (e.g., optical memory and environmental stability), it was not incorporated into the main device platform of the present study.

Overall, despite the intrinsic limitations of the simplified FTO-based architecture, the device demonstrates sufficient stability and performance for large-area, outdoor electrochromic shading applications, while also highlighting clear directions for future device optimization.

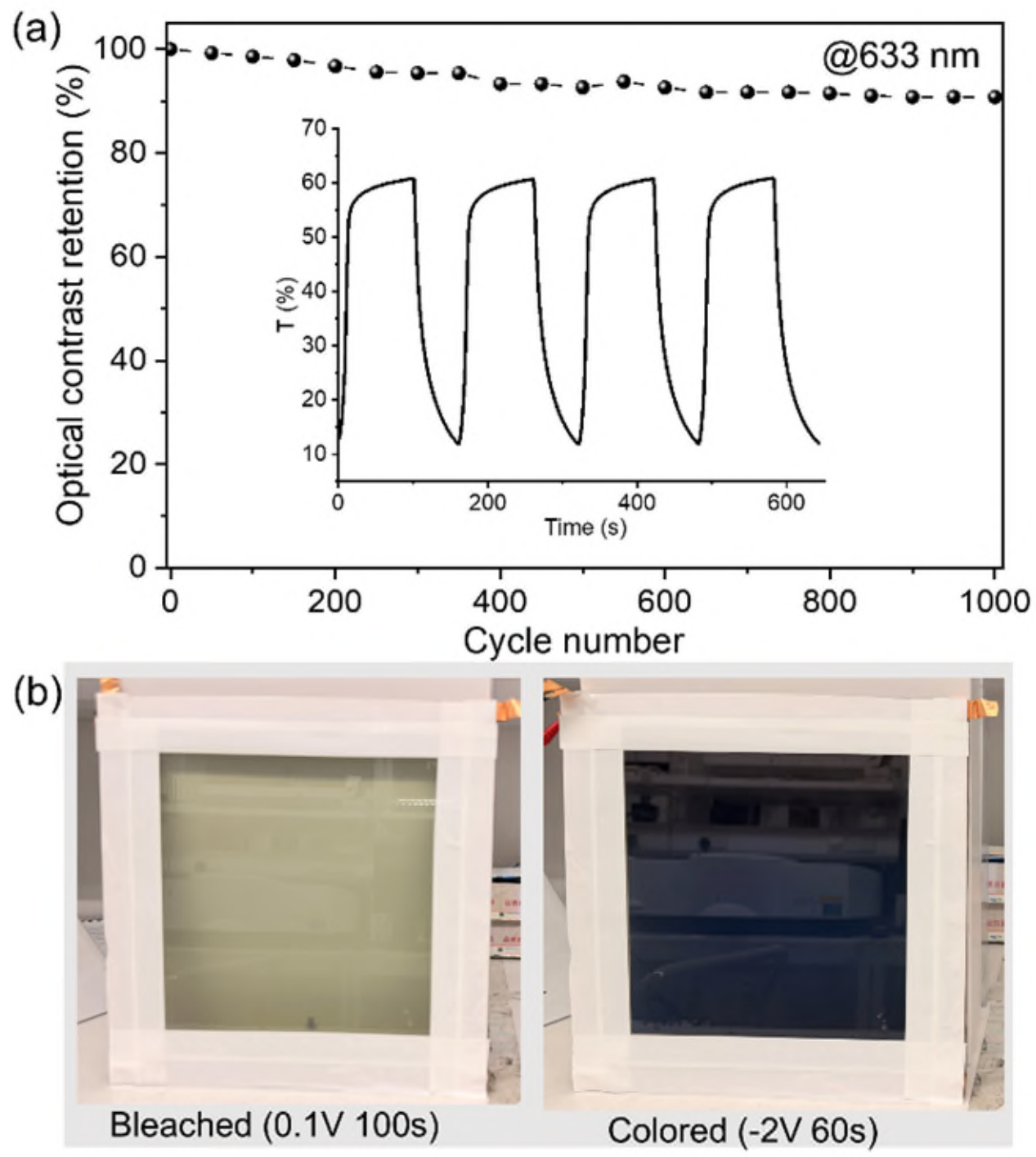

Fig. R2. Supporting control experiment using an added large-area ion-storage electrode. **a**, Optical-contrast retention at 633 nm over 1000 coloration/bleaching cycles for a 30 × 30 cm² ECD fabricated using the same printed WO_3 working electrode and the same electrolyte, with an added large-area PEDOT:PSS-based composite ion-storage electrode. The inset shows the corresponding transmittance–time response during initial representative cycles. **b**, Photographs of the device in the bleached state (0.1 V, 100 s) and colored state (−2.0 V, 60 s).

To address this comment, we have revised the manuscript to:

1. Add a comparison of the initial and post-1000-cycle T–t responses as Supplementary Fig. 4;

2. Revise the stability discussion to state that the reduced bleached-state transmittance after cycling is consistent with limited charge reversibility and irreversible ion intercalation at the bare FTO counter side:

Lines 165-170, Page 6

Comparison of the initial and after-cycling transmittance–time responses (Supplementary Fig. 4) further indicates that the degradation is mainly associated with a decrease in bleached-state transmittance, whereas the colored-state transmittance changes less

markedly. This behavior is consistent with limited charge reversibility and irreversible ion intercalation at the bare FTO counter side during repeated bleaching/coloration processes, as reported for bare FTO/ITO-based counter electrode configurations.[37,38]

3. Expand the **Discussion** part to acknowledge the architectural limitation and highlight the need for scalable ion-storage electrode materials:

Lines 392-396, Pages 15-16

Building on the above scope and limitation, several directions can further advance this technology. First, future device architectures can incorporate ion-storage electrode materials with electrochemical characteristics appropriately matched to WO_3, improving charge balance, reducing operating voltage, and further enhancing the long-term stability of large-area EC devices while remaining compatible with large-area and low-temperature fabrication.

References:

2.Wu, L., Fang, H., Li, J., Wu, L. & Wang, H. Catalyzing Viologen Electrochromic Reaction with CuOx Nanoparticles. *Advanced Materials Technologies* **8**, 2301028 (2023).

3. Jo, M.-H., Koo, B.-R., Kim, K.-H. & Ahn, H.-J. Tailored interface stabilization of FTO transparent conducting electrodes boosting electron and Li ion transport for electrochromic energy-storage devices. *Chemical Engineering Journal* **431**, 134036 (2022).

Comment #9. *Supplementary fig 3. It would be convenient to add the initial performance of the devices so it can be compared with the after two-month test.*

Response: We thank the Reviewer for this helpful suggestion. In response, we have revised Supplementary Fig. 3(**Revised Supplementary Fig. 5**) to include the initial device performance together with the performance after two months of outdoor aging, allowing direct comparison before and after exposure.

Specifically, the updated figure shows both the transmittance spectra in the colored and bleached states and the dynamic switching response (@633nm) of the ECD in the initial state and after two months of outdoor aging in Singapore. Based on this direct comparison, we further clarified in the revised manuscript that the reduction in optical contrast after aging mainly originates from the lowered bleached-state transmittance, while the switching kinetics remain comparable.

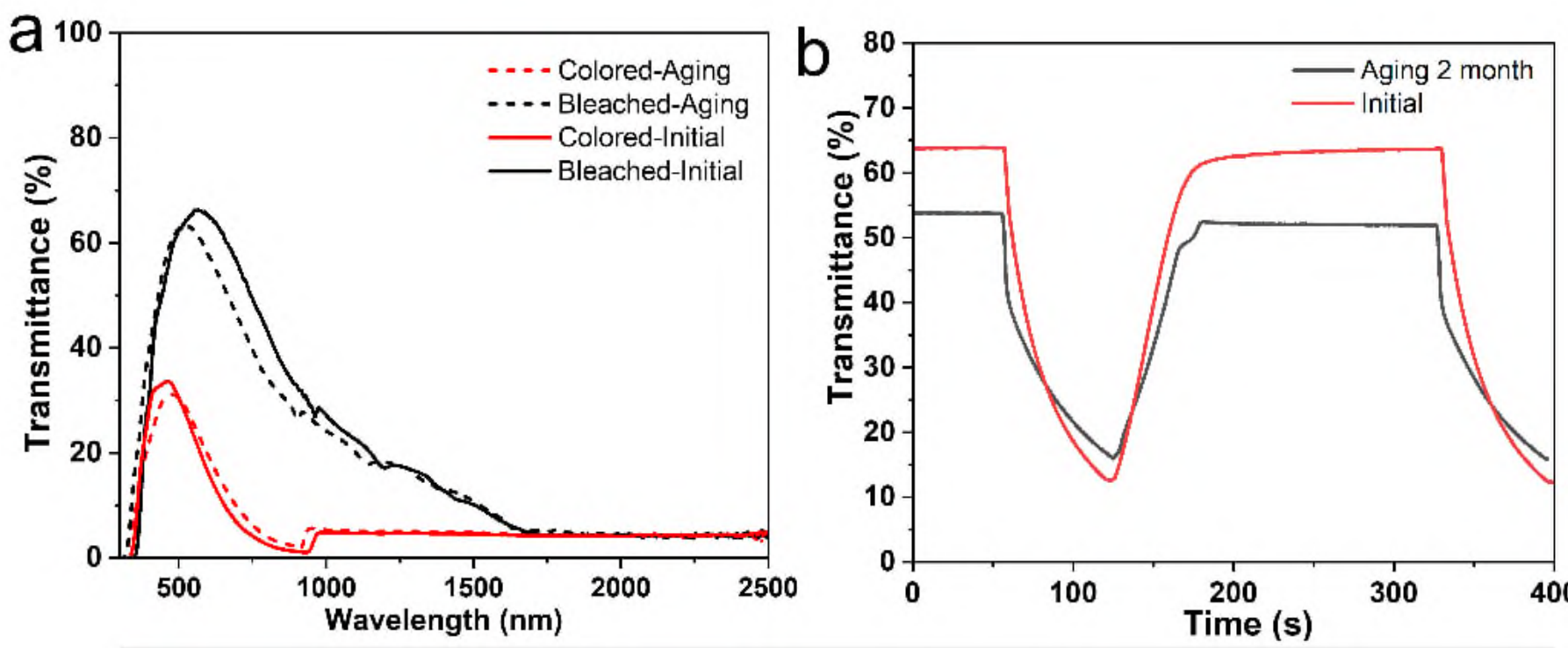

Fig. R7 (Revised Supplementary Fig. 5). Comparison of the initial and aged electrochromic device after two months of outdoor exposure in Singapore. **a**, Transmittance spectra in the colored and bleached states. **b**, In-situ transmittance response at 633nm of the initial device and the device after two months of outdoor aging.

To address this comment, we have revised the corresponding description in the manuscript:

Lines 174-182, Page 7

Additionally, outdoor durability was assessed by continuous exposure in Singapore over two months (October and November 2024) (Supplementary Fig. 5). Comparison of the initial and aged devices shows that the decrease in optical contrast mainly originates from a reduced bleached-state transmittance, while the colored-state transmittance remains largely unchanged. After outdoor aging, the ECD maintains a large optical contrast of 38.5% at 633 nm (under -3.5 V for 70 s and 2 V for 200 s), corresponding to 75.6% retention of initial optical contrast. The reduced contrast mainly arises from a lowered bleached-state transmittance, consistent with device aging under prolonged sunlight

exposure, while switching kinetics remain comparable (52.1 s for coloring and 41.5 s for bleaching), indicating retained electrochromic functionality after two months of tropical outdoor exposure.

Comment #10. *Supplementary fig 9. It is not clear what is meant with "across diverse weather conditions". What is exactly shown in the graphs? Please specify.*

Response: We thank the reviewer for this comment and agree that the phrase "across diverse weather conditions" was not sufficiently specific. In the revised Supplementary Information, we have clarified that Supplementary Fig. 9 (Revised Supplementary Fig. 11) presents three representative daily PAR time-series extracted from the full-year Singapore simulation to illustrate how the irradiance-triggered EC switching strategy performs under different sky/weather conditions.

Specifically, the top panel corresponds to an overcast day, where outdoor PAR remains relatively low (typically < ~1000 μmol m^{-2} s^{-1})[14], and the EC shading therefore stays in the bleached state throughout the day. The middle panel shows a clear-sky day, where outdoor PAR becomes high around midday, and the EC roof is triggered to the colored state during peak irradiance hours. The bottom panel represents a cloudy/variable day, where PAR fluctuates due to intermittent cloud cover and the EC roof switches states according to the dual-threshold control logic. In all cases, the EC shading reduces excessive PAR compared with the Ref roof and maintains transmitted PAR closer to the crop-optimal range (250-750 μmol m^{-2} s^{-1}).

To address this comment, we have

1. revised the caption and accompanying text to Supplementary Fig. 9 (Revised Supplementary Fig.11):

   Supplementary Fig. 11. Simulated daily PAR profiles under representative sky conditions and corresponding EC switching states. Three representative days were selected from the full-year Singapore simulation to illustrate the irradiance-adaptive control performance under different weather/sky conditions: overcast (top), clear-sky (middle), and cloudy/variable (bottom). Lines show the simulated PAR (PPFD) time series for the EC roof (blue) and reference roof (orange). Shaded bars indicate the EC roof optical state (grey: bleached; blue: colored) determined by the dual-threshold switching strategy.

   The overcast day exhibits low PAR and no coloration event, the clear-sky day triggers coloration during peak irradiance hours, and the cloudy/variable day shows intermittent switching in response to fluctuating irradiance.

2. updated the corresponding main-text description of Supplementary Fig. 11 in the manuscript: **Lines 303-306, Page 12**

> Owing to the irradiance-adaptive switching control, EC shading maintained transmitted PAR within the optimal range for lettuce growth under representative sky conditions (selected from the full-year simulation), including overcast, clear-sky, and cloudy/variable days (Supplementary Fig. 11), whereas the Ref shading frequently resulted in excessive PAR levels.

References:

14. Wang, Z., Zuo, M., Wang, Y., Liu, Y. & Li, D. Dynamics of chlorophyll fluorescence and eco-morphological properties of Microcystis bloom in Meiliang Bay of Lake Taihu, China. *Fresenius Environmental Bulletin* **20**, 2 (2011).

Comment #11. *Line 379. Please correct (-3.5 V/ 2.0 V).*

Response: We thank the Reviewer for pointing this out. We have corrected this expression in the revised manuscript:

Lines 439-440, Page 17

> This setup enables the automated coloring and bleaching of the ECD by applying the converted DC voltages (−3.5 V/2.0 V).

Comment #12. *Line 371. Please correct "is detailed introduced".*

Response: We thank the Reviewer for pointing this out. We have corrected the grammatical error in the revised manuscript:

Lines 441-443, Page 17

> a corresponding flowchart with operational parameters (Supplementary Fig. 15 and Supplementary Table 4) is introduced in detail in the Supplementary information.

Reviewer #3 (Remarks to the Author):

Manuscript ID: NCOMMS-26-004207-T

Title: Scalable all-weather electrochromic shading for adaptive photothermal regulation in urban agriculture

The manuscript presents a research study proposing the use of a self-powered electrochromic device to regulate temperature and incoming radiation in order to improve lettuce production and quality. A comprehensive characterization is provided, including structural and optical analyses, together with a full implementation of the proposed device. The manuscript also addresses applicability under different climatic conditions and scalability aspects through simulations, further supporting the practical relevance of the proposed approach. The results clearly demonstrate the suitability of the approach for the intended application and, in my view, represent outstanding outcomes, supported by very clear and well-designed figures, graphs, and photographs. Therefore, I recommend the manuscript for publication after minor revisions. Please find below some specific comments and queries:

We thank the referee for this positive and constructive feedback and appreciate encouraging assessment of the significance, clarity, and practical relevance of this work. Listed below are the reviewer's comments and our responses. We hope that we have addressed all of the reviewer's comments.

Comment #1. *Some acronyms are not defined or are inconsistently defined throughout the manuscript. Please verify and clarify, namely "EC", "DLI", and "Ref".*

Response: We thank the Reviewer for this helpful comment. We have carefully checked the definitions and usage of the acronyms throughout the manuscript and revised them for clarity and consistency.

"EC" has been used consistently for "electrochromic" after its definition, and related expressions in the title, section headings, and figure captions have been further standardized. To avoid ambiguity, the abbreviation EC previously used for **ethylene carbonate** in the Materials section has been removed, and the chemical name is written in full.

We also verified the use of "DLI", which is consistently defined as "daily light integral". For "Ref", we retained this abbreviation consistently to denote the "reference" system, such as "Ref testbed",

“Ref shading”, and “Ref tower”, as these refer to different structural components of the same reference setup. In addition, the capitalization of Ref has been standardized throughout the manuscript.

Comment #2. *The scientific name of lettuce appears twice using different formats. Please revise for consistency.*

Response: We thank the Reviewer for pointing this out. We agree that the lettuce cultivar name was presented inconsistently in the original manuscript. In response, we have revised the relevant text to use Green Span consistently throughout the manuscript:

Lines 474-475, Page 18

> Lettuce seeds (*Green Span*, Arianetech Pte Ltd) were germinated at 22 °C with an 18:6 h light/dark photoperiod and a constant photosynthetic photon flux density by LED lighting.

Comment #3. *In Figure 1, the caption could be improved for clarity. In particular, the description of Figure 1d should more clearly guide the reader, explicitly referring to the left panel and to the colour graphs (left, centre, and right). Moreover, in the left graph of Figure 1d, additional data corresponding to the use of PE film only should be included, similarly to what is presented in the right graph.*

Response: We thank the Reviewer for this helpful suggestion. We have revised Fig. 1d to improve both completeness and clarity. Specifically, we have added the PE film data to the left graph of Fig. 1d, so that the PAR level and the corresponding red/green/blue contributions can be directly compared among unshaded sunlight, PE film, and the EC shading in both the bleached and colored states.

We have also revised the figure caption to more clearly guide the reader through the left, centre, and right graphs of Fig. 1d. The updated caption describes the content of each panel, including the PAR photon flux comparison, the transmittance spectra, and the transmitted solar energy in the UV, VIS, and NIR regions.

In addition, we have reorganized and clarified the main-text description associated with Fig. 1d to improve the logical flow and better distinguish the different functions illustrated in the three graphs.

To address this comment, we have revised

1. Fig.1d and corresponding caption:

Lines 138-142, Page 6

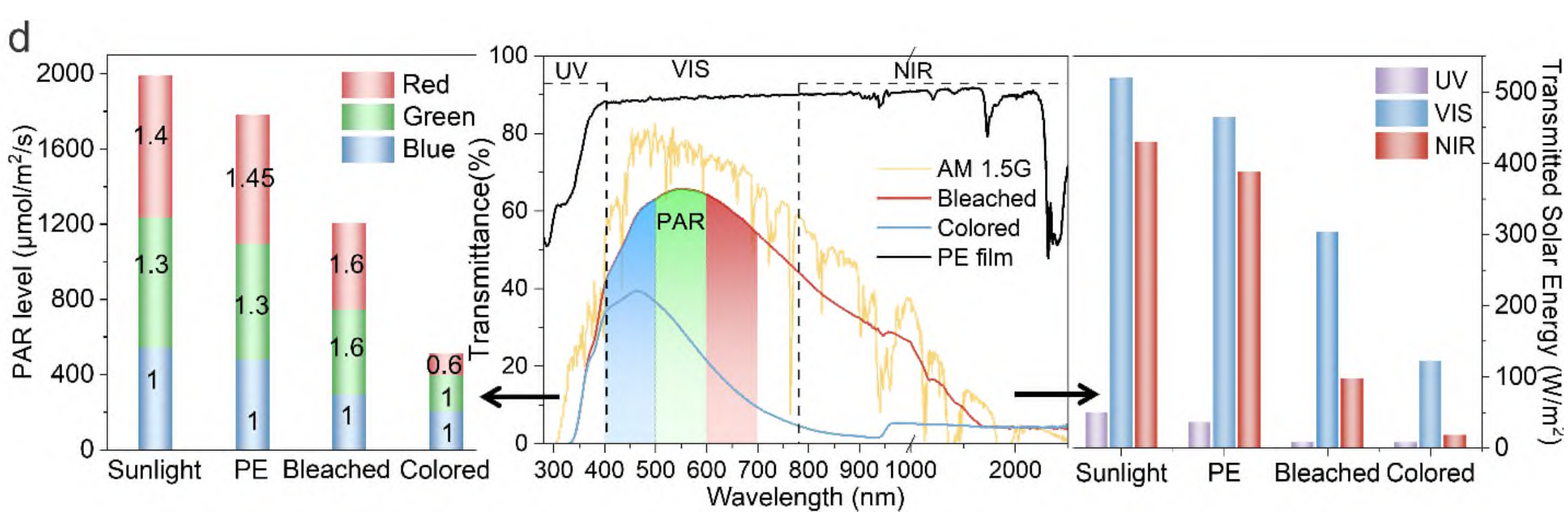

Fig. R8 (Revised Fig. 1d) Left, calculated PAR photon flux levels under unshaded sunlight, commercial PE film, and the EC shading in colored/bleached states, with the corresponding red, green, and blue photo-flux contributions indicated in each bar. **Centre,** transmittance spectra (0.28-2.5 μm) of the EC shading in colored/bleached states, together with commercial PE film, overlaid with the AM 1.5G solar spectrum; the

PAR region is highlighted. **Right,** calculated transmitted solar energy in the UV/VIS/NIR regions under unshaded sunlight, PE film, and EC shading in colored/bleached states. AM1.5G, air mass 1.5 global.

2. Main-text description associated with Fig. 1d:

Lines 100-131, Pages 4-5

For outdoor operation, the ECD state is autonomously controlled by a light-sensor-based module powered by an integrated solar panel, enabling irradiance-adaptive switching under changing outdoor conditions. This state transition provides coordinated photothermal regulation by reducing radiative heat gain through passive UV suppression and NIR attenuation, while tuning both the intensity and spectral composition of PAR (Fig. 1c, d). As shown in Fig.1d (centre and right), the ECD in both colored and bleached states strongly suppresses UV transmission and maintains low NIR transmittance, while providing switchable visible light transmission. Mechanistically, UV suppression is dominated by the passive filtering of the FTO-glass substrates (Supplementary Fig. 1), whereas NIR attenuation is also substantial in both states and primarily governed by the FTO-glass baseline, with additional moderate electrochromic modulation. These effects are quantified for the complete device using EN 410-type band-integrated metrics (Supplementary Table 1): the ECD blocks 83.7%/81.8% (colored/bleached) of UV radiation (280–400 nm), and attenuates 96.3%/77.6% (colored/bleached) of NIR radiation (780–2500 nm), respectively. This spectral attenuation is further reflected in the reduced transmitted solar energy across these regions, leading to a lower radiative heat load (Supplementary Table 2).

In addition, the switchable visible states enable dynamic regulation of PAR intensity toward the crop-optimal range (Fig. 1a) and of spectral quality. As summarized in Fig 1d (left) and Supplementary Table 3, the EC shading modulates both the total PAR photon flux and the relative red (600-700 nm), green (500-600 nm), and blue (400-500 nm) contributions. Under high solar irradiance, the EC shading switches to the colored state, increasing the relative blue light fraction (B/R=1:0.6) compared with unshaded sunlight (B/R=1:1.4). Such a blue-enriched spectrum is known to modulate stomatal behaviour and photoprotective signalling,[28,29,] and is therefore expected to support photosynthetic performance under fluctuating irradiance. Conversely, under low solar irradiance, the EC shading reverts to the bleached state with a higher relative red-light fraction (B/R=1:1.6), which can favour efficient light harvesting and support carbon assimilation under light-limited conditions,[30] thereby benefiting plant growth. This state-dependent shift in B/R ratio is consistent with established photobiological responses of plants, where blue- and red-light fractions differentially regulate stomatal behaviour and photosynthetic acclimation.

In contrast, commercial PE film transmits most NIR and VIS radiation with minimal spectral selectivity or dynamic modulation. As shown in Fig. 1d (centre and right), the PE film exhibits ~90% transmittance over 400-2250 nm and allows 888.2 W/m^2 of solar energy to enter the growing chamber under standard AM 1.5G solar irradiation (1000

W/m^2), providing minimal regulation of thermal load. Consistently, Fig. 1d (left) shows that the PE shading maintains a fixed B/R ratio (1:1.45), indicating limited adaptability of the light environment compared with the EC shading.

Comment #4. *In line 193, the authors state that the DLI under EC shading was 10 mol m⁻² day⁻¹; however, this value corresponds only some of the tested days. It should be clearly stated that the DLI under EC conditions fell within the recommended range only on a limited number of test days, although it remained closer to the optimal range than in the outdoor or Ref conditions.*

Response: We thank the Reviewer for this careful observation. We agree that the original wording overstated the DLI result under EC shading. In the revised manuscript, we have clarified that the DLI under EC shading reached the recommended range only on several test days, while remaining overall much closer to this range than the Ref and outside conditions, both of which consistently experienced excessive light exposure.

To address this comment, we have revised the corresponding content:

Lines 215-222, Page 8

> On the representative day shown in Fig. 3b (24 October 2024), the DLI under EC shading was 10.0 mol/(m^2·d), much closer to the recommended range of 10-15 mol/(m^2·d) for lettuce growth than that in the Ref testbed (22.1 mol/(m^2·d)) or the unshaded outside environment (31.1 mol/(m^2·d)).[41] During the growing period (October 15–28, 2024), the DLI under EC shading reached the recommended range on several test days, while remaining overall much closer to this range than the Ref testbed and the unshaded outside environment, both of which consistently experienced excessive light exposure. Overall, EC shading maintained light conditions that were substantially more favourable than those in the Ref and outside conditions, as reflected by both DLI and PAR values (Fig. 3c and Supplementary Fig. 6).

Comment #5. *In Figure 3, for improved readability, I recommend adding visual guides (e.g., dashed lines or shaded regions) indicating the optimal values or ranges for crop production, since the current description of this figure is somewhat dense and extensive.*

Response: We thank the Reviewer for this helpful suggestion. In response, we have revised Fig. 3 to improve readability by adding visual guides indicating the optimal PAR/DLI ranges for crop production. Specifically, in Fig. 3b, we added a marked region showing the optimal PAR range for lettuce growth (250–750 μmol/m²/s), and in Fig. 3c, we added dashed guide lines indicating the recommended DLI range for lettuce growth (10–15 mol/(m²·d)).

We have also revised the corresponding figure caption to explicitly explain these graphical guides. In addition, the related main-text description has been updated to align more clearly with the revised figure, so that the discussion of PAR regulation and DLI optimization has been more directly connected to the optimal ranges shown in Fig. 3b,c.

To address this comment, we have revised

1. Fig.3b, c, and corresponding caption:

Lines 233-242, Page 9

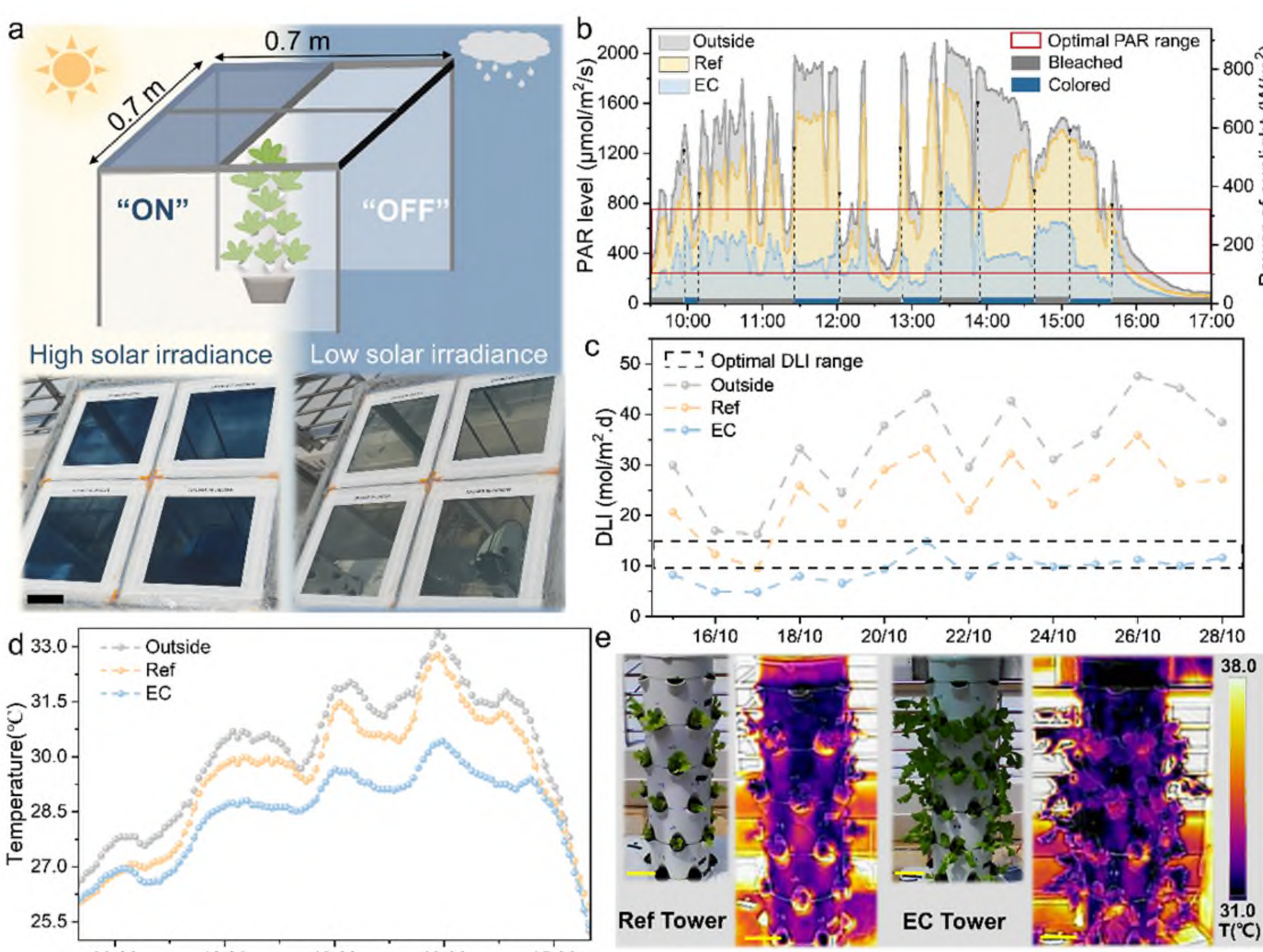

Fig.R9 (Revised Fig. 3)| Light and temperature performances of the testbed with EC and Ref shading. a, Photograph of the meter-scale EC/Ref testbed with ECDs and PE film as roof shading under high/low solar irradiance conditions. Scale bar:10 cm. **b,** Temporal variation of PAR in the EC/Ref testbeds and unshaded outside environment over a representative growing day (9:00-17:00, October 24, 2024), together with the corresponding outdoor solar power. Grey and blue bars at the bottom indicate the bleached and colored states of the EC shading. The red boxed region indicates the optimal PAR range for lettuce growth (250-750 μmol/m²/s). **c,** DLI values of the EC/Ref testbeds and unshaded outside environment during the plant cultivation period (October 15–28, 2024). The black dashed lines indicate the recommended DLI range for lettuce growth (10-15 mol/(m²·d)). **d,** Comparison of air temperature in EC/Ref testbeds and outside environment recorded every 5 minutes from 7:30 to 18:00 on October 20. **e,** The IR images of the EC/Ref towers with growing lettuce on October 23 at 11:30 am. Scale bar:10cm.

2. main-text description associated with Fig. 3b, c:

Lines 210-222, Page 8

Despite this hysteresis behavior, the control strategy effectively maintains PAR in the EC testbed largely within the optimal range for lettuce growth (250-750 μmol/m²/s). By contrast, PAR levels in the Ref testbed and the outdoor environment frequently exceeded the lettuce light-saturation threshold (750 μmol/m²/s). EC shading also significantly optimized the daily light integral (DLI), which represents the total amount of PAR received per day and is a key determinant of plant growth and productivity. On the representative day shown in Fig. 3b (24 October 2024), the DLI under EC shading was 10.0 mol/(m²·d), much closer to the recommended range of 10-15 mol/(m²·d) for lettuce growth than that in the Ref testbed (22.1 mol/(m²·d)) or the unshaded outside environment (31.1 mol/(m²·d)).[41] During the growing period (October 15–28, 2024), the DLI under EC shading reached the recommended range on several test days, while remaining overall much closer to this range than the Ref testbed and the unshaded outside environment, both of which consistently experienced excessive light exposure. Overall, EC shading maintained light conditions that were substantially more favourable than those in the Ref and outside conditions, as reflected by both DLI and PAR values (Fig. 3c and Supplementary Fig. 6).

Point-to-Point Response to Reviewers' Comments

The reviewers' comments are marked in **black**, and the author's responses are in **blue**.

Reviewer #1 (Remarks to the Author):

Comments:

Dear Authors

after this round of revisions, your manuscript has been improved and, in my opinion, can be considered suitable for publication in this journal.

Kind regards.

Response: We sincerely thank the reviewer for the positive evaluation of our revised manuscript and for the constructive comments provided throughout the review process.

Reviewer #2 (Remarks to the Author):

Comments:

I congratulate the authors for the great effort put towards addressing all reviewer comments. The manuscript has been considerably improved. From my side, I consider that all my comments have been adequately responded, and the manuscript is ready for publication.

Response: We sincerely thank the reviewer for the encouraging comments and high evaluation of our work.

Reviewer #3 (Remarks to the Author):

Comments:

The authors are acknowledged for the efforts on addressing my comments. From my viewpoint, the manuscript can now be accepted.

Response: We sincerely thank the reviewer for the positive assessment of our revised manuscript.
